# mTORC1 Transcriptional Regulation of Ribosome Subunits, Protein Synthesis, and Molecular Transport in Primary Human Trophoblast Cells

**DOI:** 10.3389/fcell.2020.583801

**Published:** 2020-11-26

**Authors:** Fredrick J. Rosario, Theresa L. Powell, Madhulika B. Gupta, Laura Cox, Thomas Jansson

**Affiliations:** ^1^Division of Reproductive Sciences, Department of Obstetrics and Gynecology, University of Colorado Anschutz Medical Campus, Aurora, CO, United States; ^2^Section of Neonatology, Department of Pediatrics, University of Colorado Anschutz Medical Campus, Aurora, CO, United States; ^3^Department of Biochemistry, University of Western Ontario, London, ON, Canada; ^4^Center for Precision Medicine, Department of Internal Medicine, Section of Molecular Medicine, Wake Forest School of Medicine, Winston-Salem, NC, United States

**Keywords:** placenta, maternal–fetal exchange, human, nutrient sensor, energy metabolism, gene array

## Abstract

Mechanistic Target of Rapamycin Complex 1 (mTORC1) serves as positive regulator of placental nutrient transport and mitochondrial respiration. The role of mTORC1 signaling in modulating other placental functions is largely unexplored. We used gene array following silencing of raptor to identify genes regulated by mTORC1 in primary human trophoblast (PHT) cells. Seven hundred and thirty-nine genes were differentially expressed; 487 genes were down-regulated and 252 up-regulated. Bioinformatic analyses demonstrated that inhibition of mTORC1 resulted in decreased expression of genes encoding ribosomal proteins in the 60S and 40S ribosome subunits. Furthermore, down-regulated genes were functionally enriched in genes involved in eIF2, sirtuin and mTOR signaling, mitochondrial function, and glutamine and zinc transport. Stress response genes were enriched among up-regulated genes following mTORC1 inhibition. The protein expression of ribosomal proteins RPL26 (RPL26) and Ribosomal Protein S10 (RPS10) was decreased and positively correlated to mTORC1 signaling and System A amino acid transport in human placentas collected from pregnancies complicated by intrauterine growth restriction (IUGR). In conclusion, mTORC1 signaling regulates the expression of trophoblast genes involved in ribosome and protein synthesis, mitochondrial function, lipid metabolism, nutrient transport, and angiogenesis, representing novel links between mTOR signaling and multiple placental functions critical for normal fetal growth and development.

## Introduction

Placental nutrient sensing is the process by which the syncytiotrophoblast cell integrates a multitude of maternal and fetal nutritional cues through cellular nutrient sensing signaling pathways to balance fetal nutrient demand with the ability of the mother to provide nutrients and oxygen required for fetal growth (Jansson et al., 2012a). This is accomplished by regulating maternal physiology and functions such as nutrient transport (Jansson and Powell, 2006, 2013; Jansson et al., 2012b; Larque et al., 2013) in the syncytiotrophoblast, the epithelium that separates maternal and fetal blood.

Mechanistic Target of Rapamycin (mTOR) is an evolutionarily conserved serine/threonine kinase that is activated by amino acids, folate, glucose, oxygen, and growth factor signaling and promotes cell growth and metabolism. mTOR exists in distinct two complexes, mTOR Complex 1 (mTORC1) and 2, with the protein raptor associated to mTORC1 and rictor associated to mTORC2. When activated, mTORC1 phosphorylates S6K1 and 4E-BP1 promoting protein translation, lipid biogenesis, and metabolism as well as suppressing autophagy (Peng et al., 2002; Jacinto and Hall, 2003; Hay and Soneneberg, 2004; Martin and Hall, 2005; Tee and Blenis, 2005; Laplante and Sabatini, 2012). mTORC2 phosphorylates Akt, PKCα, and Serum and Glucocorticoid-regulated Kinase 1 (SGK1) and regulates cytoskeletal organization and metabolism (Jacinto et al., 2004; Alessi et al., 2009). Placental mTOR activity is decreased in human intrauterine growth restriction (IUGR) (Roos et al., 2007; Yung et al., 2008; Chen et al., 2015a) as well as in rodent (Rosario et al., 2011) and non-human primate models of IUGR (Kavitha et al., 2014). On the other end of the fetal growth spectrum, placental mTOR activity is activated in obese women giving birth to larger babies (Jansson et al., 2013) as well as in a mouse model of maternal obesity associated with fetal overgrowth (Rosario et al., 2015b). We previously demonstrated that placental mTOR functions as a positive regulator of amino acid transporter systems A and L (Rosario et al., 2013), folate transporters (Rosario et al., 2016), and mitochondrial respiration (Rosario et al., 2019). However, mTORC1 regulation of other trophoblast functions remains largely unexplored.

Although well established as a master regulator of protein translation, mTORC1 signaling is also involved in transcriptional regulation. For example, mTORC1 modulates gene expression by impacting the activity of transcription factors, including STAT3, TFEB, NRF1, HIF1α, and YY1-PGC1α (Peng et al., 2002; Cunningham et al., 2007; Duvel et al., 2010; Jimenez et al., 2010; Wang et al., 2011; Laplante and Sabatini, 2013). Moreover, pharmacological and genetic studies have demonstrated that mTOR binds to the promoter and regulates the synthesis of Pol I-transcribed 45S rDNA and Pol III-transcribed genes, including genes critical for protein synthesis and cell growth such as tRNA and 5S rRNA (Tsang et al., 2010).

The human placenta expresses more than 12,000 genes, including most of the known imprinted genes (Dizon-Townson et al., 2000). Common pregnancy complications, such as preeclampsia, IUGR, preterm birth, and recurrent pregnancy loss, are associated with impaired placentation and/or altered placental function. Integrative transcriptome analysis revealed dysregulation of canonical cancer molecular pathways in the placenta in association with preeclampsia (Moslehi et al., 2013). Emerging evidence demonstrates that placental function and gene expression are altered in response to various stresses and perturbations in the maternal environment (Osei-Kumah et al., 2011). For example, feeding mice a high fat diet was reported to influence placental gene expression (Mao et al., 2010). In addition, supplementation with dietary n-3 long chain polyunsaturated fatty acids to pregnant women altered the placental transcriptome (Sedlmeier et al., 2014). To the best of our knowledge, the regulation of the trophoblast transcriptome by mTORC1 signaling has not been previously explored. In the present study, we employed an unbiased discovery approach to identify regulatory networks and novel regulators to attain a more comprehensive understanding of mTORC1 regulation of gene expression in primary human trophoblast (PHT) cells.

## Materials and Methods

### Ethical Approval and Study Participants

All experimental protocols were approved by the Institutional Review Board of the University of Texas Health Science Center San Antonio. Placentas of uncomplicated term pregnancies were collected with informed consent at the Labor and Delivery Unit at the University Hospital San Antonio.

### Isolation and Culture of Primary Human Trophoblast (PHT) Cells

Placentas were collected immediately following delivery by cesarean section at term without labor. PHT cells were isolated and cultured *in vitro* using well-established protocols (Kliman et al., 1986). Cells were plated in 60 mm culture dishes (∼7.5 × 10^6^ cells/dish) and cultured in 5% CO_2_, 95% atmosphere air at 37°C for 90 h. Cell culture media (DMEM/Hams F-12, supplemented with L-glutamine, penicillin, streptomycin, gentamycin, and 10% fetal bovine serum) was changed daily. Four independent biological replicates (*n* = 4 placentas) were studied. Selected clinical characteristics of the study subjects are provided in Table 1.

**TABLE 1:** Selected clinical characteristics of pregnancies used for isolation of trophoblast cells.

	Placenta 1	Placenta 2	Placenta 3	Placenta 4
Maternal age (years)	26.0	27.0	25.0	28.0
BMI (kg/m^2^)	24.5	23.5	22.5	23.0
Gestational age (weeks)	37.1	37.2	37.3	37.5
Birth weight (g)	2500	2645	2589	2679
Placental weight (g)	645	680	595	604
Fetal sex (M/F)	M	F	M	F
Mode of delivery (C/V)	C	C	C	C

*Abbreviations: F, female; M, male; C, cesarean section; V, vaginal delivery.*

### Assessment of Biochemical Differentiation and Viability

To confirm that trophoblast cells were undergoing biochemical differentiation, and to assess their viability with time in culture, the release of human chorion gonadotropin (hCG) by trophoblast cells into the culture medium 18, 42, 66, and 90 h after plating was measured using a commercial ELISA kit, which detects the β-subunit of hCG (Immuno Biological Labs). As a readout of apoptosis, protein expression of caspase-3 was determined using Western blot.

### RNA Interference-Mediated Silencing

Dharmafect 2 transfection reagent (Thermo Scientific, Rockford, IL, United States) and small interfering RNAs (siRNAs) (Sigma–Aldrich, St. Louis, MO, United States), targeting raptor (100 nM; sense, 5′CAGUUCACCGCCAUCUACA) was used. Control cells were transfected with a non-coding scrambled sequence (100 nM; sense: 5′GAUCAUACGUGCGAUCAGATT). siRNA was added to cultured primary trophoblast cells after 18 h in culture, incubated for 24 h, and removed when fresh medium was added to wells (Forbes et al., 2009).

### Western Blotting

Efficiency of target silencing was determined at the protein (expression of raptor) and functional levels (phosphorylation of mTORC1 down-stream target; S6-Serine-235/236) using Western blot. A 90 h culture PHT cells were rinsed once with ice-cold PBS and lyzed in ice-cold buffer (PBS containing protease and phosphatase inhibitors and 0.05% SDS). Subsequently, cells were scraped, collected, and sonicated. The soluble fraction of cell lysates was isolated by centrifugation at 13,000 *g* for 10 min at 4°C. Total protein concentration in the cell lysate was determined using Bradford’s reagent (Bio-Rad). Cell lysate proteins (10 μg) were separated on 4–20% precast linear gradient gels (Invitrogen). Membranes were incubated overnight at 4°C with primary antibody diluted in 1% non-fat milk (wt/vol) in TBST and detected using an appropriate peroxidase-conjugated secondary antibody. Products were visualized by ECL chemiluminescence (Millipore). Band intensities were measured using the G-box system (Syngene). Anti-raptor, S6-S-235/236, Akt-S-473 antibodies were obtained from Cell Signaling. Ribosomal Protein L26 (RPL26) and Ribosomal Protein S10 (RPS10) antibodies were obtained from AB clonal. Target band densities were normalized to total protein (Ponceau) stain as a loading control. For each protein target, the mean density of the control sample bands was assigned an arbitrary value of 1. All individual densitometry values were expressed relative to this mean.

### RNA Isolation From PHT Cells

RNA was isolated from cultured PHT cells at 90 h in culture using TRIzol Reagent (Invitrogen, Carlsbad, CA, United States) according to the manufacturer’s instructions. RNA was resuspended in 100 μl DEPC-treated water. RNA quality was determined using an Agilent 2100 Bioanalyzer (Agilent Technologies, Inc., Santa Clara, CA, United States) and RNA concentrations confirmed by quantitation using a NanoDrop^TM^ 8000 spectrophotometer (Thermo Fisher Scientific, Wilmington, DE, United States).

### Gene Expression Profiling in PHT Cells

Whole genome expression profiling was performed using gene arrays (HumanHT-12 v4 Expression BeadChips, Illumina Inc., San Diego, CA, United States). cRNA was synthesized and biotin labeled (cat. no. 1750, Ambion, Austin, TX, United States) according to manufacturer’s instructions. Total RNA was used for first and second strand cDNA synthesis followed by *in vitro* transcription to synthesize biotin-labeled cRNA. cRNA was quality checked and then hybridized to Human HT-12 v4 Expression BeadChips (Illumina Inc.). Individual cRNA samples were used to interrogate each BeadChip (Scramble siRNA, *n* = 4; Raptor siRNA, *n* = 4). Gene expression was detected and cleaned using GenomeStudio software (Illumina Inc.) and filtered using quality score > 0.95. Gene array data were all-median normalized and log_2_ transformed (GeneSifter), and differentially expressed genes were identified by *t*-test (*p* < 0.05).

### Pathway Analysis

Genes significantly different in expression between raptor silenced and scramble siRNA treated PHT were overlaid onto Kyoto Encyclopedia of Genes and Genomes (KEGG) pathways (Kanehisa and Goto, 2000; Kanehisa et al., 2016, 2017) using GeneSifter. Z-scores were calculated in GeneSifter. Statistical enrichment of differentially expressed genes in pathways was determined by calculating z-scores using the following formula: z-score = (*r*−*n*(*R*/*N*))/SQRT(*n*(*R*/*N*)(1−(*R*/*N*))(1−(*n*−1/*N*−1)), where *R* = total number of genes meeting selection criteria, *N* = total number of genes measured, *r* = number of genes meeting selection criteria with the specified GO term, and *n* = total number of genes measured with the specific GO term. Pathway enrichment analysis of differentially expressed genes has been shown to reveal pathways that are impacted in that biological system (Bayerlova et al., 2015). KEGG is a comprehensive knowledge base for assisting in the biological interpretation of large-scale molecular datasets. Using KEGG pathway enrichment analysis provides a means to statistically filter gene expression data within the framework of annotated biological systems (Kanehisa et al., 2017). Calculations were performed using Gene Sifter software default settings without user input. Pathways were considered significantly different between groups if the z-score for that pathway was greater than 2.0 or less than 2.0 (Doniger et al., 2003). Biological pathways were mapped by using online tool KEGG^1^ (Kyoto encyclopedia for genes and genomes) (Ogata et al., 1999; Kanehisa and Goto, 2000; Kanehisa et al., 2016, 2017).

### Network Analysis

The networks were generated through the use of IPA (QIAGEN Inc.^2^) (Kramer et al., 2014). Network analysis [Ingenuity Pathway Analysis (IPA), Ingenuity^®^ Systems, Redwood City, CA, United States] was performed using differentially expressed genes (*p* < 0.05) from each pairwise comparison. Networks were built using the IPA Knowledge Base, using expression profiles from this dataset and requiring direct connections between molecules based on experimental evidence (Kramer et al., 2014). Network significance was calculated in IPA using Fisher exact *t*-test (Ingenuity^®^ Systems). The *p*-value for a given network takes into account the number of eligible molecules (differentially expressed genes) in the selected reference set (defined by the Ingenuity Knowledge Base); the total number of molecules in the selected reference set known to be associated with that function; the total number of eligible molecules in the selected reference set; and the total number of molecules in the reference set (Ingenuity^®^ Systems). In this analysis, we considered networks containing > 25 differentially expressed genes and a *p*-value < 10^–20^ as significant.

### Placental mTORC1 Signaling and Expression of Ribosomal Proteins in IUGR

Placentas from pregnancies complicated by IUGR and women delivering appropriate-for-gestational age (AGA) infants were collected within 15 min of delivery as described (Chen et al., 2015a). Selected clinical data for the AGA and IUGR groups are provided in Table 2. There was no significant difference in maternal age, body mass index (BMI), or gestational age between the control and the IUGR groups. Birth weight was 28% lower (*P* < 0.01) and placental weight was reduced by 36% (*P* < 0.001) in the IUGR group compared with AGAs. The decidua basalis and chorionic plate were removed, and villous tissue was dissected and rinsed in cold physiological saline. The villous tissue was transferred to cold buffer D (250 mM sucrose, 10 mM HEPES, pH 7.4) containing 1:100 dilution of protease and phosphatase inhibitors (Sigma–Aldrich, St. Louis, MO, United States) and homogenized on ice with a Polytron (Kinematica, Luzern, Switzerland). Placental homogenates were frozen in liquid nitrogen and stored at −80°C until further processing. The phosphorylation of key proteins in the mTORC1 [as reported in Chen et al., 2015a] and ribosomal protein (RPS10 and RPL26) expression in placental homogenates of IUGR and AGA groups were determined using Western blots as described above for PHT cells.

**TABLE 2:** Selected clinical data.

	AGA (*n* = 19)	IUGR (*n* = 25)
Maternal age (years)	25.9 ± 1.29	28.7 ± 1.23
BMI (kg/m^2^)*	28.3 ± 2.6	26.8 ± 2.0
Gestational age (weeks)	33.9 ± 0.95	35.7 ± 0.61
Birth weight (g)	2493 ± 236	1804 ± 110^†^
Birth weight percentile^‡^	55.9 ± 4.6	2.4 ± 0.3^§^
Placental weight (g)	566 ± 42.0	394 ± 18.4^*parallel*^
Fetal sex (M/F)	7/12	8/17
Mode of delivery (C/V)	6/13	15/10

Data are presented as means ± SEM. Abbreviations: AGA, appropriate grown for gestational age; IUGR, intrauterine growth restriction; F, female; M, male; C, cesarean section; V, vaginal delivery. *Data from *n* = 10 AGA and 18 IUGR. ^‡^By corresponding gestational age. ^†^*P* < 0.05. ^∥^*P* < 0.01. ^§^*P* < 0.0001.

### Data Presentation and Statistics

The number of experiments (*n*) represents the number of individual placentas studied. Data are presented as means ± SEM. Array data from each sample were all-median normalized and log_2_ transformed. Box plots were inspected to ensure that the median for each group was 0 and variance among groups was similar. Statistical analyses of array data were performed by *t*-test using Gene Sifter software (Geospiza, Inc.) for pairwise comparisons (GeneSifter.Net, VizX Labs, Seattle, WA, United States; GEO accession number: GSE40878). Statistical significance of differences between control and experimental groups in studies of protein expression and ribosomal protein was assessed using Student’s *t*-test. A *P*-value < 0.05 was considered significant.

## Results

### Raptor Silencing in PHT Cells Inhibits mTORC1 Signaling

**FIGURE 1:**
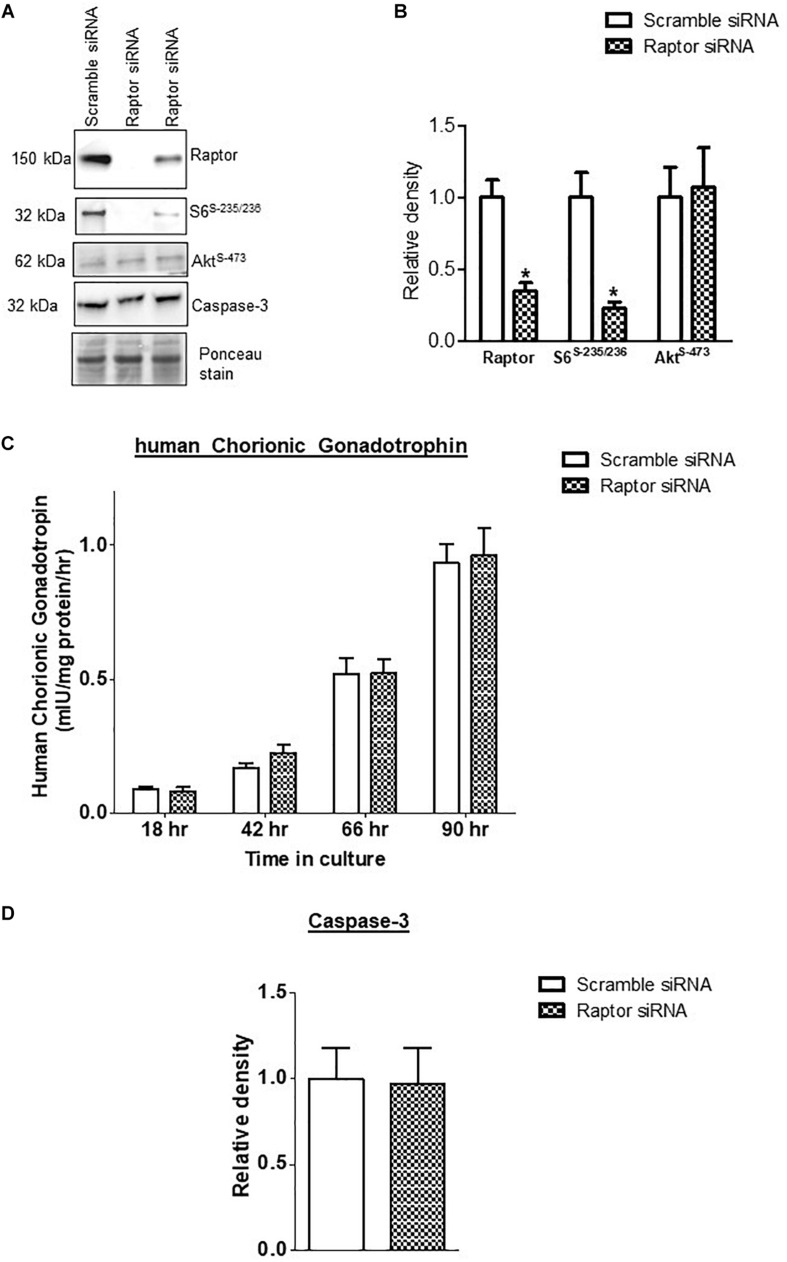
Effect of raptor silencing on raptor protein expression, mTORC1 signaling activity, and trophoblast differentiation and viability. **(A)** Representative western blots of raptor, phosphorylated S6 ribosomal protein (Ser-235/236) and total S6 ribosomal protein, and Caspase-3 expression in cell lysates of scramble siRNA and raptor siRNA silenced cells. Equal loading was performed. **(B)** Summary of the western blot data of raptor, S6 ribosomal protein (Ser-235/236), and total S6 ribosomal protein. **(C)** Secretion of human chorionic gonadotropin (hCG) from PHT cells transfected with scramble or raptor siRNA. **(D)** Summary of the western blot data of Caspase 3. Values are given as means + SEM. *P < 0.05 vs. scramble *siRNA*; unpaired Student’s *t-*test; *n* = 4/each group.

*Raptor* siRNA markedly decreased the protein expression of raptor (−75%, *p* = 0.001; *n* = 4/each group) and decreased the phosphorylation of S6 ribosomal protein-Ser-235/236 (−84%, *p* = 0.002; *n* = 4/each group), a functional readout for mTORC1 signaling (Figure 1). In contrast, raptor silencing did not influence mTORC2 signaling, using Phospho-Akt serine 473 as a functional readout (Figure 1) demonstrating that raptor silencing is specific for mTORC1 signaling and does not result in a secondary activation of mTORC2. The hCG secretion profiles (*n* = 4/each group) and caspase-3 (*n* = 4/each group) expression were comparable between PHT cells transfected with scrambled or raptor siRNA (Figure 1). These findings indicate that raptor silencing did not affect differentiation/syncytialization and viability of cultured PHT cells and suggest that the effects of raptor silencing on the trophoblast transcriptome were not caused by unspecific effects.

### Genes Regulated by mTORC1 Signaling in PHT Cells

**FIGURE 2:**
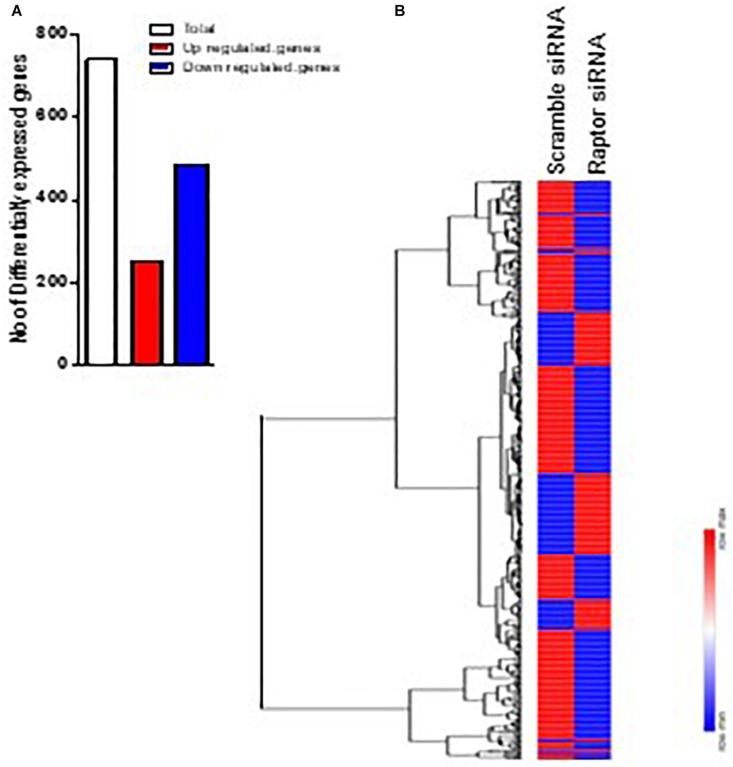
Differentially expressed genes in raptor siRNA vs scramble siRNA silenced PHT cells. **(A)** Heatmap of expression data for the differentially expressed genes in raptor siRNA vs. scramble siRNA silenced PHT cells. Clustering of gene expression patterns reveals genes with functional correlations. **(B)** The number of differentially expressed genes in raptor siRNA vs. scramble siRNA silenced PHT cells. The red represents up-regulated genes and blue represents down-regulated genes in raptor silenced PHT cells as compared to cells transfected with scramble siRNA.

The impact of mTORC1 inhibition (raptor silencing) on PHT gene expression was determined by analyzing gene array data comparing *raptor* siRNA with scramble siRNA. Inhibiting mTORC1 significantly altered the expression of 739 genes, 252 were up-regulated and 487 were down-regulated (Figure 2). Significantly impacted genes are listed in Supplementary Table 1.

### Hierarchical Clustering of Differentially Expressed Genes (DEGs)

**FIGURE 3:**
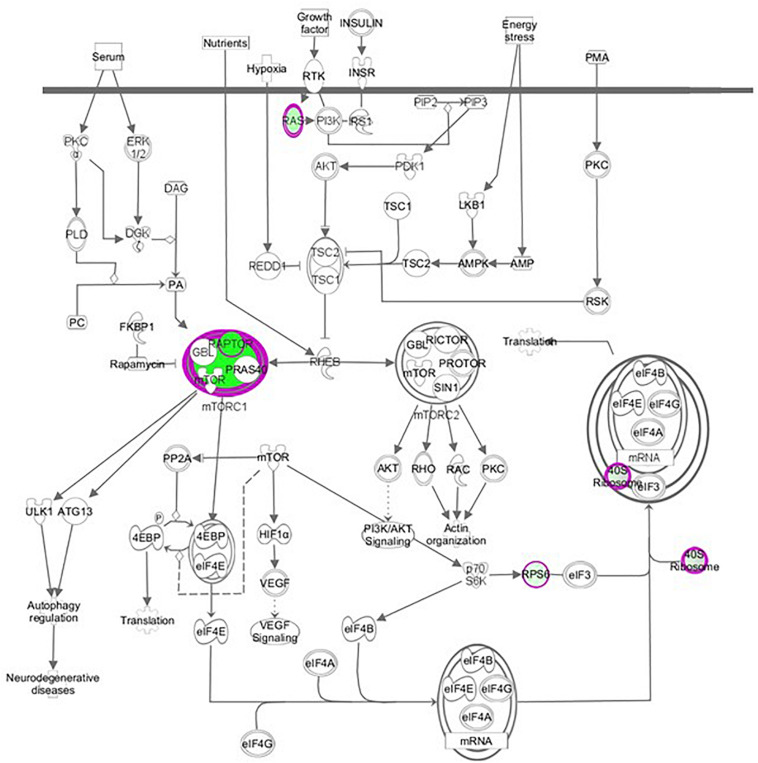
Raptor silencing down-regulated components of mTORC1 (RPTOR, RPS15 A, RPS28) but not mTORC2 signaling in PHT cells. Graphical representation of PHT cell mTOR signaling networks identified by Ingenuity Pathway Analysis (IPA) following raptor silencing. Molecular relationships between genes down-regulated (green) or up-regulated (red) or no change (white) in expression after raptor silencing are shown.

We performed a hierarchical clustering of differentially expressed genes using the heat map function. Rows correspond to genes and columns to samples. In the heat map of Figure 2B, 739 differential genes were analyzed. The genes with similar expression patterns are clustered together. Among genes significantly up-regulated in raptor silenced cells were those coding for proteins involved in vascular smooth muscle contractions (*PLA2G3*, *PPP1R12C*, *CYP4A11*, *ROCK2*, *ADM*), interleukin signaling pathways (*IL-6*), transcriptional regulators of EGF-dependent pathways (*ATF3*), and cellular response to stress (*HLADQA*, *KIR3DL1*, and *GPR150*). Among the genes significantly down-regulated in raptor silenced cells were those coding for proteins involved in glutathione metabolism (*GSTK1*, *MGST3*, *SMS*, *GGCT*) and metabolic processes (*AKR1A1*, *ACOT4*, *GALM*, *CMBL*, *COX6C*, *COX7B*, *COX7C*, *B3GALNT2*). Importantly, raptor silencing was confirmed in the gene array by decreased expression of RPTOR, whereas expression of *RICTOR* was unaffected (Figure 3).

### Functional Analysis of Differentially Expressed Genes in PHT Cells With mTORC1 Inhibition

Gene ontology (GO) analysis of differentially expressed genes using the ‘ShinyGOv06.1 bioinformatics tool provides insights into biological processes, molecular functions, and cellular components. Separate GO enrichment analysis for up-regulated and down-regulated genes was performed. GO analysis suggested that up-regulated genes were mainly involved in biological processes including response to stress and regulation of biological quality (Table 3), whereas down-regulated genes primarily played a role in catabolic process, cellular component biogenesis, and regulation of signaling (Table 4). In the cellular component category, many down-regulated transcripts were associated with vesicle, cytosolic ribosome, ribosomal subunit, extracellular exosome/organelle/vesicle, and endomembrane system (data not shown).

**TABLE 3:** Gene Ontology biological classification statistics of up-regulated genes in raptor silenced PHT cells.

Number of genes	High level Gene Ontology category	Genes
39	Response to stress	*USP28 DEPDC5 BDKRB1 REC8 MASP1 OASL FIGN APOBEC3C UBE2V1 EPB41L4B MCM8 MDM2 IL6 TRIM26 PNPT1 ALAS2 ATF3 RFWD3 SPN CR1 ADCYAP1R1 PSEN1 CADM4 EZH2 GLP1R COL4A3BP MORC1 NFKBIZ ADM ISG20 NUB1 KIR3DL1 ROCK2 LGALS3BP SH2B1 LCK PRTN3 LILRB3 HLA-DQA2*
32	Regulation of response to stimulus	*CC2D1A MIB2 DEPDC5 MASP1 FIGN LCK SPRY4 IL6 EZH2 GRM4 MDM2 PNPT1 LGR5 ADM NETO1 NF2 CR1 ADCYAP1R1 PSEN1 CADM4 ARHGEF17 NFKBIZ RASA1 ATF3 SH2B1 RASAL3 RFWD3 UBE2V1 ROCK2 ICOS KIR3DL1 HLA-DQA2*
32	Regulation of biological quality	*MDGA1 USP28 PSEN1 SCN1B GLP1R CPLX2 SCN3B KLK1 MDM2 IL6 RASA1 ADM ADCYAP1R1 BDKRB1 EZH2 COL4A3BP LNPEP SPTBN5 CIZ1 ALAS2 NETO1 NF2 RASL10B LCK CYP4A11 FA2H MCM8 ROCK2 ZNF16 GRM4 SH2B1 PRTN3*
30	Cellular localization	*COL4A3BP C16ORF70 RABL2A CPLX2 LCA5L TSNARE1 PACS2 TBC1D3C EXOC3L2 GGA1 PSRC1 MDM2 ADCYAP1R1 PSEN1 PLA2G3 BDKRB1 EZH2 CIZ1 NETO1 NF2 ROCK2 PAF1 MCM8 SCN3B LCK HAO2 GRM4 SPTBN5 RPL32 TCTN2*
30	Regulation of molecular function	*SCN1B OASL LGR5 SCN3B SPRY4 TBC1D3C PSEN1 EZH2 GRM4 IL6 TRIM26 NETO1 ZNF16 LCK CR1 ADCYAP1R1 DEPDC5 RASAL3 PPP1R12C PSRC1 MDM2 RASA1 ADM DCP1B NF2 SPINK9 ROCK2 PRTN3 UBE2V1 GLP1R*
29	Immune system process	*MASP1 OASL LCK APOBEC3C IL6 PAF1 HOXA7 TRIM26 PRTN3 SPN CR1 LILRB3 PSEN1 PLA2G3 BDKRB1 NFKBIZ CPLX2 ALAS2 ICOS ISG20 HLA-DQA2 NUB1 RASAL3 KIR3DL1 ZNF16 COL4A3BP LNPEP SLC7A10 UBE2V1*

**TABLE 4:** Gene Ontology biological classification statistics of down-regulated genes in raptor silenced PHT cells.

Number of genes	High level Gene Ontology category	Genes
76	Catabolic process	*PSMB1 SAMD4A ACAT1 ECHDC1 RBX1 ZC3H14 PSMA2 AKR1A1 MMP19 GALM QDPR HK3 SMPD1 ATG7 UBA52 APOC2 DECR1 NUDT1 SKP1 SPP1 RPL23 CYP19A1 AZIN1 PRDX3 HPRT1 ACOT4 BCAP31 APOBEC3G VPS41 SIRT2 SNX5 TREM2 TSPO PLA2G15 BLVRA APAF1 PLBD1 DDA1 NPL HINT2 ESD TMEM208 LSM3 VDAC1 PGAM4 PDXP YBX1 FEZ2 NRBP2 ATP6V0E1 RPS20 TYMP DYNLL1 RPL18A LSM5 RPS12 PCCB RPL22 RPL21 RPS10 CHMP2A RPS15A GLUL RPS6 SEC13 RPL26 PTTG1 FABP5 ALDH1A1 FABP4 RPS9 RPS27 RPL12 RPL23A RPS28 CEBPA*
74	Cellular component biogenesis	*VPS41 RPL26L1 GAR1 NDUFB3 NCKAP1L RPS10 ORMDL1 DYNC2LI1 SRP19 C10ORF90 VBP1 RPL26 MAPRE2 LSM3 RSL1D1 RPS27 NDUFAF3 ATG7 RPL12 RPL23A RPS28 PPM1A DECR1 UGDH RTN4 RAB32 KHDRBS1 PRMT1 TRA2B APIP GBP5 SEC13 H3F3A HPRT1 RXRA PDXP SIRT2 CNGB1 ACAT1 KCNC4 APAF1 GTF3A GLUL BIN1 POLR1E RPS6 GCHFR G3BP2 PTPRO PDLIM5 GPBAR1 TSPYL1 EIF3CL NDUFB2 RAP2A NDUFA1 CHMP2A NDUFB9 FCER1G FEZ2 HAUS7 FGD2 TCP1 HSPB11 DYNLL1 RBX1 TRIP11 SKP1 CENPL TUBA1A RPS9 NDUFV2 UBA52 APOC2*
60	Regulation of signaling	*ATP2C1 LGALS1 PPM1A LGALS9 LRP5L DUSP2 CCKAR TXN TREM2 EZH2 AAK1 SPP1 RAP2A CYP19A1 TBC1D16 S100A4 TYMP SNX5 CA2 RTN4 RPL22 KCNC4 NENF APAF1 KHDRBS1 CHSY1 SERINC3 HAVCR2 GLUL CTDSPL2 SOD1 FGD2 APIP PTPRO SEC13 FABP5 MAPRE2 IL16 ATP2A2 UFSP2 PFDN5 RPL23 DSTYK ARL6IP5 RPL26 SMPD1 BCAP31 LILRA5 PRMT1 PSMB1 GDI2 CNGB1 RBX1 PSMA2 ATP1B1 IFNAR2 BRD7 FPR1 ARHGAP30 UBA52*

### Ingenuity Pathway Analysis

**FIGURE 4:**
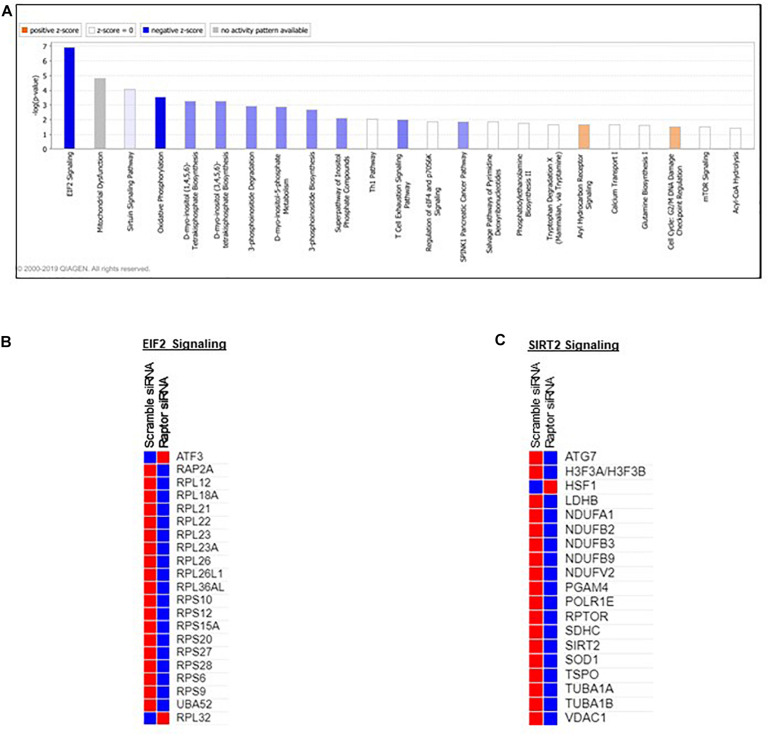
Overall functional analysis. Analysis of the top molecular and cellular functions determined by IPA for the raptor siRNA silenced PHT cells. **(A)** Ingenuity Pathway Analyses (IPA) of array data identified canonical pathways significantly affected by raptor silencing in PHT cells. **(B,C)** ER stress and SIRT2 signaling response genes are prominently affected in raptor silenced PHT cells. Color index represents gene expression changes, the red represents up-regulated genes, and the blue represent down-regulated genes in raptor silenced PHT cells as compared to cells transfected with scramble siRNA.

**FIGURE 5:**
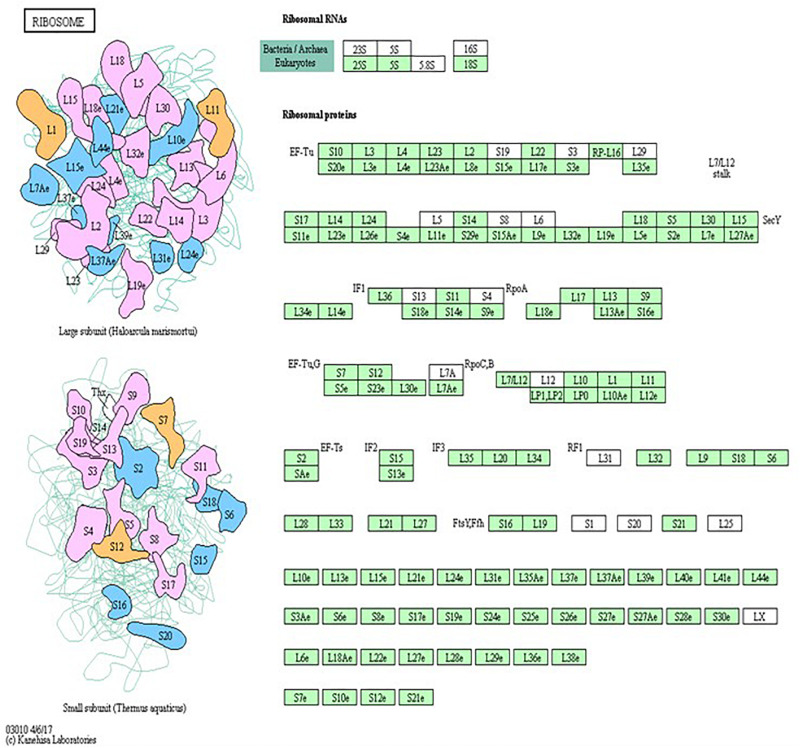
Raptor silencing induces expression changes in ribosome subunit genes. KEGG pathway database regulation of ribosome biogenesis is over-represented in raptor silenced PHT cells as compared to cells transfected with scramble siRNA. The green box indicates down-regulated genes in raptor silenced PHT cells as compared to cells transfected with scramble siRNA. The black box indicates no significant change in expression.

Pathway analysis is considered a valuable tool in estimating functions of genes in different systems (Allocco et al., 2004). IPA revealed distinct GO enrichment in *raptor* silenced cells. The top significantly enriched canonical signaling pathways inhibited in raptor-silenced PHT cells were eIF2 signaling, mitochondria dysfunction, sirtuin signaling, and mTOR signaling (Figure 4), consistent with mTORC1 being a key regulator of protein synthesis and oxidative phosphorylation (Thoreen et al., 2012; Rosario et al., 2019). We used KEGG analysis to study mTORC1 regulation of genes encoding for ribosomal proteins in greater detail and found that nineteen genes were identified as being significantly inhibited in raptor silenced PHT cells. Specifically, Ribosomal protein S6 (*RPS6*), L26 like (*RPL26L1*), L12 (*RPL12*) L18a (*RPL18A*), L21 (*RPL21*), L22 (*RPL22*), L23A (*RPL23A*), L26 (*RPL26*), L36a like (*RPL36AL*), S9 (*RPS9*), S10 (*RPS10*), S12 (*RPS12*), S15a (*RPS15A*), S20 (*RPS20*), S27 (*RPS27*), S28 (*RPS28*), and L23 (*RPL23*) as well as ubiquitin A-52 residue ribosomal protein fusion product 1 (*UBA52*) were down-regulated (Figure 5), suggesting that inhibition of mTORC1 results in a broad and coordinated down-regulation of genes encoding proteins involved in ribosomal function.

### mTORC1 Inhibition Regulated an Array of Molecular Signaling Pathways in PHT Cells

Up-regulated (252) and down-regulated genes (487) were mapped to the KEGG pathway database^3^. KEGG pathway analysis of cells with raptor silencing identified 183 down-regulated (Table 5) and 54 up-regulated pathways (Table 6). Down-regulated pathways in response to mTORC1 inhibition included amino sugar and nucleotide sugar metabolism, biosynthesis of unsaturated fatty acids, cysteine and methionine metabolism, glycolysis/gluconeogenesis, glyoxylate and dicarboxylate metabolism, oxidative phosphorylation, purine metabolism, pyrimidine metabolism, ribosome, RNA polymerase, vitamin B6 metabolism, and sphingolipid metabolism (Table 5). Furthermore, alpha-linoleic acid metabolism, arachidonic acid metabolism, cell adhesion molecule, retinol metabolism, RNA degradation, and vascular smooth muscle contraction were among the pathways up-regulated in response to mTORC1 inhibition (Table 6).

**TABLE 5:** Down-regulated KEGG pathways in PHT cells in response to *raptor* silencing.

KEGG pathways Down	Diff regulated genes	Up	Down	Gene set	z-score (Down)
Amino sugar and nucleotide sugar metabolism	4	0	4	47	2.65
Biosynthesis of unsaturated fatty acids	3	0	3	21	3.47
Butirosin and neomycin biosynthesis	1	0	1	5	2.51
Cardiac muscle contraction	5	0	5	73	2.4
Cysteine and methionine metabolism	3	0	3	35	2.31
Glutathione metabolism	4	0	4	50	2.5
Glycolysis/Gluconeogenesis	5	0	5	65	2.7
Glycosphingolipid biosynthesis – ganglio series	2	0	2	15	2.69
Glyoxylate and dicarboxylate metabolism	4	1	3	18	3.86
Metabolic pathways	54	5	49	1080	4.76
Oxidative phosphorylation	11	0	11	119	4.76
Propanoate metabolism	3	0	3	32	2.5
Purine metabolism	10	2	8	157	2.11
Pyrimidine metabolism	7	1	6	95	2.4
Ribosome	19	1	18	88	10.87
RNA polymerase	3	0	3	28	2.79
Vitamin B6 metabolism	1	0	1	5	2.51

*Table lists KEGG pathways that were significantly down-regulated in PHT cells with raptor silencing as compared to control cells. “List” denotes the total number of differentially expressed genes on the array. “Up” denotes the number of up-regulated genes. “Down” denotes the number of down-regulated genes. “Gene set” denotes the total number of genes in this pathway that are included on the array and give a signal with PHT cell RNA. “Z-score” denotes the z-score for the list pathway.*

**TABLE 6:** Up-regulated KEGG pathways in PHT cells in response to *raptor* silencing.

KEGG pathways Up	Diff regulated genes	Up	Down	Gene Set	z-score (Up)
alpha-Linolenic acid metabolism	1	1	0	17	2.38
Antigen processing and presentation	3	2	1	66	2.08
Arachidonic acid metabolism	2	2	0	55	2.4
Cell adhesion molecules (CAMs)	5	4	1	127	3.05
Complement and coagulation cascades	3	3	0	68	3.4
Graft-vs.-host disease	4	3	1	35	5.23
Intestinal immune network for IgA production	4	3	1	44	4.55
Neuroactive ligand–receptor interaction	9	6	3	312	2.34
Other glycan degradation	1	1	0	17	2.38
Primary immunodeficiency	2	2	0	35	3.31
Renin–angiotensin system	1	1	0	17	2.38
Retinol metabolism	3	2	1	51	2.55
RNA degradation	5	2	3	69	2
Staphylococcus aureus infection	5	2	3	51	2.55
Vascular smooth muscle contraction	5	5	0	123	4.16

*Table lists KEGG pathways that were significantly up regulated in PHT cells with raptor silencing as compared to control cells. “List” denotes the total number of differentially expressed genes on the array. “Up” denotes the number of up-regulated genes. “Down” denotes the number of down-regulated genes. “Gene set” denotes the total number of genes in this pathway that are included on the array and give a signal with PHT cell RNA. “Z-score” denotes the z-score for the list pathway.*

### Network Analysis

Network analysis of RNA expression in PHT cells with inhibition of mTORC1 (raptor silencing) compared with control cells (scramble) revealed three networks (Supplementary Figures 1–3). The top annotated functions of the genes composing the networks included: lipid metabolism, nutrient transport, small molecule biochemistry, connective tissue disorder, developmental disorder, hereditary disorder, post-translational modification, cell signaling, drug metabolism, cell morphology, cellular compromise, cellular function and maintenance, metabolic disease, carbohydrate metabolism, embryonic development, organismal development, tissue development, DNA replication, recombination, and repair, neurological disease, organismal injury, and abnormalities. Evaluation of these networks showed a coordinated response to raptor silencing with the majority of genes down-regulated (Supplementary Figures 1–3).

### Placental mTORC1 Signaling Is Associated With the Protein Expression of RPL26 and RPS10 in Human Pregnancy

**FIGURE 6:**
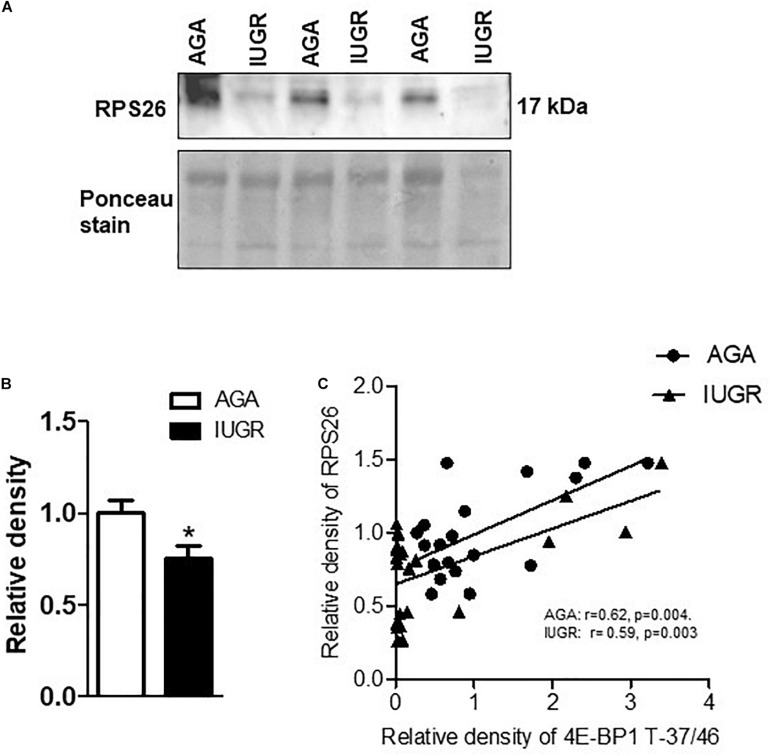
Expression of RPL26 in placental homogenates and correlation between placental mTORC1 functional readout 4EBP-T-37/46 and protein expression of RPL26. **(A)** Protein expression of RPL26 in placental homogenates of AGA and IUGR group. Representative Western blot is shown. **(B)** Relative expression of RPL26 in placental homogenates of AGA and IUGR. After normalization to β-actin, the mean density of AGA samples was assigned an arbitrary value of 1. Subsequently, individual IUGR density values were expressed relative to this mean. **(C)** Correlation between placental mTORC1 functional readout 4E-BP1 T-37/46 and RPL26 expression. *r* = Pearson correlation coefficient, *n* = AGA, 19; IUGR, 25. Values are given as means ± SEM; *P < 0.05 vs. AGA; unpaired Student’s *t*-test.

**FIGURE 7:**
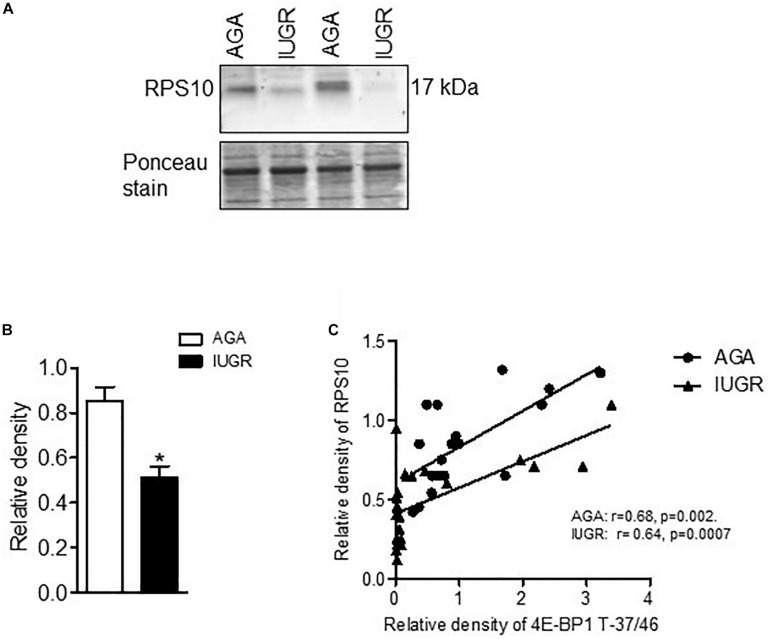
Expression of RPS10 and correlation of placental mTORC1 functional readout 4EBP-T-37/46 and protein expression of RPS10. **(A)** Protein expression of RPS10 in placental homogenates of AGA and IUGR group. Representative Western blot is shown. **(B)** Relative expression of RPS10 in placental homogenates of AGA and IUGR. After normalization to β-actin, the mean density of AGA samples was assigned an arbitrary value of 1. Subsequently, individual IUGR density values were expressed relative to this mean. **(C)** Correlation between placental mTORC1 functional readout 4E-BP1 T-37/46 and RPSL6 expression. *r* = Pearson correlation coefficient, *n* = AGA, 19; IUGR, 25. Values are given as means ± SEM; *P < 0.05 vs. AGA; unpaired Student’s *t*-test.

To explore the clinical relevance of our findings, we examined the relationship between placental mTORC1 signaling and protein expression of ribosomal proteins RPL26 and RPS10 in placentas collected from AGA and IUGR pregnancies. The protein expression of RPL26 (Figure 6) and RPS10 (Figure 7) was significantly reduced in IUGR placentas. Placental mTORC1 signaling in the same placentas has been reported elsewhere (Chen et al., 2015a). Phosphorylated 4E-BP1 (Thr-37/46) was used as a placental mTORC1 signaling functional readout and was positively correlated with RPL26 (Figure 6) and RPS10 (Figure 7) expression in AGA and IUGR placentas.

### Placental Protein Expression of RPL26 and RPS10 Is Associated With Microvillus Membrane (MVM) System A Amino Acid Transport in Human Pregnancy

**FIGURE 8:**
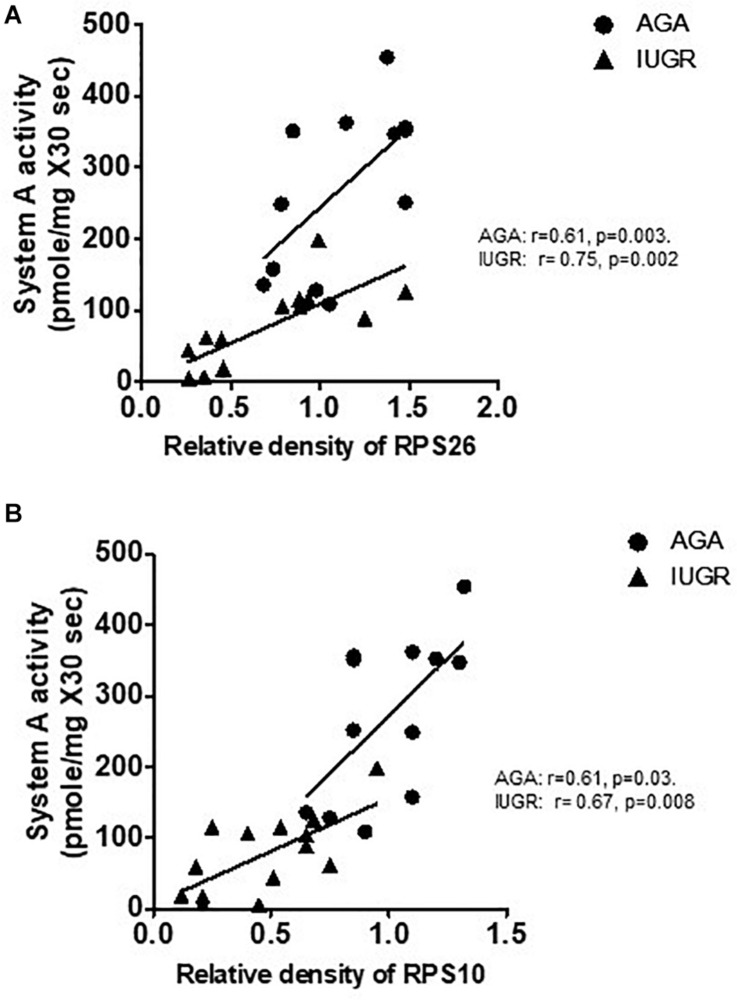
Correlation between placental protein expression of ribosomal protein RPL26/RPS10 and microvillus membrane system A amino acid transport in human pregnancy. **(A)** Correlation between placental protein expression of RPL26 and MVM system A amino acid transport in AGA and IUGR human pregnancy. **(B)** Correlation between placental protein expression of RPS10 and MVM system A amino acid transport in AGA and IUGR human pregnancy. *r* = Pearson correlation coefficient, *n* = AGA, 12; IUGR, 14.

System A is a ubiquitous Na^+^-dependent transporter that transports small, zwitterionic, neutral amino acids with short, unbranched side chains, such as alanine, serine, and glutamine (Johnson and Smith, 1988). The activity of system A has been consistently shown to be lower in syncytiotrophoblast microvillous plasma membranes (MVM) isolated from IUGR placentas (Mahendran et al., 1993; Glazier et al., 1997; Chen et al., 2015a). We examined the relationship between placental protein expression of ribosomal proteins RPL26 and RPS10 and MVM system A amino acid transport in placentas collected from AGA and IUGR pregnancies. Placental MVM system A amino acid transport in the same placentas has been reported elsewhere (Chen et al., 2015a). As shown in Figure 8, placental protein expression of RPL26 and RPS10 was positively correlated with MVM system A amino acid transport in AGA and IUGR placentas.

## Discussion

We report for the first time mTORC1 regulation of the trophoblast transcriptome in PHT cells, which contributes to the relevance of our studies to human pregnancy. mTORC1 inhibition predominantly resulted in down-regulation of genes (66% of the differentially expressed genes) and these genes encode primarily for ribosome subunits and proteins involved in protein synthesis, oxidative phosphorylation, and nutrient transport. These findings agree with the well-established role of mTOR signaling in modulating cell metabolism, growth, and proliferation. In contrast, genes involved in responses to stress were enriched among up-regulated genes following raptor silencing. In addition, we provide evidence that placental expression of ribosomal proteins RPL26 and RPS10 was decreased and positively correlated to mTORC1 signaling activity and system A amino acid transport in placentas from pregnancies complicated by IUGR. These findings suggest that our findings in cultured trophoblast cells have relevance to clinically important pregnancy complications.

The eIF2 alpha and eIF4-p70S6K signaling pathways were significantly down-regulated by mTORC1 inhibition in PHT cells. This finding has potential clinical relevance because of reports in the literature describing an association between inhibition of placental eIF2 alpha-eIF4-mTOR signaling and restricted fetal growth (Yung et al., 2008; Chen et al., 2015b). The eIF2, eIF4, and p70S6K signaling pathways overlap and play an essential role in the regulation of translation. We observed a significant reduction in the transcript levels of genes encoding a number of ribosomal proteins in the 60S (*RPL30*, *RPL10A*, *RPL38*, *RPL22*, *RPL26*, *RPL18A*, *RPL12*, *RPL31*) and 40S (*RPS12*, *RPS15A*, *RPS10*, *RPS14*, *RPS6*, *RPS27*) ribosome subunits in response to mTORC1 inhibition in PHT cells, which is consistent with previous reports demonstrating that mTORC1 controls the synthesis of ribosomal proteins in other cell types (Iadevaia et al., 2012).

Knocking down of *RPS10* and *RPL26* in HeLa cells led to decreased levels of 18S rRNA, indicating that they are necessary for the assembly of the small subunit (Doherty et al., 2010). Other studies demonstrated alterations of pre-RNA processing and decreased assembly of small or large ribosomal subunits in human cells with RPS10 and 26 deficiency (Collins et al., 2018; Liu et al., 2018). We demonstrated that protein expression of ribosomal proteins RPL26 and RPS10 was decreased in human IUGR placentas. Furthermore, global deficiency in RPS10 and RPL26 genes is associated with growth retardation and congenital malformations (Clinton and Gazda, 1993). It is possible that reduction of *RPS10* and *RPL26* in IUGR placenta may affect the function of the proteins in rRNA processing, ribosome biogenesis, and protein synthesis. Thus, mTORC1 signaling inhibition in IUGR placentas(Chen et al., 2015b; Rosario et al., 2015a) could potentially result in compromised function since these cellular subunits play a central role in protein synthesis and cellular energetics (Rosario et al., 2019). In general agreement with this suggestion, several studies have provided evidence for inhibition of protein synthesis in different tissues in IUGR (Du et al., 2005; Regnault et al., 2005).

Raptor silencing also resulted in decreased expression of genes encoding proteins involved in the initiation of protein synthesis. For example, the transcript levels of EIF3C/EIF3L complex, which activates protein synthesis, and the expression of *YBX1*, which regulates translation, were significantly decreased in PHT cells with mTORC1 inhibition. The protein synthetic capacity of cells depends on the abundance of ribosomes and transfer RNAs (tRNAs). Transcription of ribosomal RNAs (rRNAs) and tRNAs by RNA polymerases I and III (Pol I and Pol III) accounts for as much as 80% nuclear transcriptional activity and is under tight control by growth factors and nutrients through the mTOR pathway (Warner, 1999). The expression levels of *POLR2*, which encodes the seventh largest subunit of RNA polymerase II (*RNAPII*), were significantly reduced in response to raptor silencing. In addition, the expression of *GPN3*, a small GTPase required for proper localization of *RNAPII*, was reduced in PHT cells following mTORC1 inhibition. Collectively, these observations are in line with a well-established role of mTORC1 in regulating protein translation and demonstrate, for the first time, that inhibition of mTORC1 signaling results in down-regulation of multiple genes encoding for proteins in the translational machinery in PHT cells. Given the reports demonstrating placental mTOR signaling is reduced in pregnancies complicated by IUGR (Yung et al., 2008; Chen et al., 2015a; Rosario et al., 2015a; Dimasuay et al., 2017; Hung et al., 2017) and activated in placentas associated with fetal overgrowth (Jansson et al., 2013; Martino et al., 2016; Muralimanoharan et al., 2016; Sati et al., 2016; Shang and Wen, 2018), it is possible that the transcriptional regulation of placental protein translation contributes to these abnormal fetal growth phenotypes in pregnant women.

One interesting observation from our study was that mTORC1 inhibition decreased the abundance of the transcript for *BPGM* (2, 3-bisphosphoglycerate mutase), an enzyme generating 1,3-BPG, which promotes release of oxygen from maternal hemoglobin at the maternal-fetal interface. Decreased BPGM expression may impair oxygen transfer across the placental barrier (Pritlove et al., 2006) when mTORC1 signaling is inhibited.

Fatty acid transporter 1 (*FATP1*) is a member of the solute carrier family 27, which facilitates the cellular uptake of long-chain fatty acids and *FABP4* regulates intracellular lipid trafficking and placental lipid transport and accumulation (Makkar et al., 2014). Interestingly, the expression of *FATP1*, *FABP4*, and *SREBP1*, which is a transcriptional activator required for lipid homeostasis, was decreased in raptor silenced PHT cells. These findings are consistent with previous reports that mTORC1 promotes lipid synthesis by activating the transcription factor *SREBP-1* (Bakan and Laplante, 2012) and identifies mTORC1 as a key regulator of placental lipid transport and metabolism.

*VEGF* signaling controls vascular function and angiogenesis, which are critical processes for normal placental function and fetal development (Cheung, 1997). Our data show that mTORC1 inhibition decreases the expression of *VEGF* in PHT cells, which is in agreement with previous reports showing that rapamycin inhibits *VEGF* production and signaling in mice adenocarcinoma (Guba et al., 2002). These observations are consistent with the finding that IUGR induced by maternal dexamethasone treatment in the rat is associated with mTORC1 inhibition and decreased expression of angiogenic factors in the placenta (Ozmen et al., 2015). Furthermore, mTORC1 inhibition in PHT cells decreased the expression of *CRELD1*, a member of a subfamily of epidermal growth factor-related proteins. This may be relevant for placental angiogenesis because *CRELD1* knock out in mice caused placental abnormalities and global vascular insufficiency in the fetus (Natale et al., 2006). Collectively, these data implicate trophoblast mTORC1 in promoting placental angiogenesis.

Down-regulated transcripts belonging to plasma membrane cellular component category included *KCNC*, *SLC38A6*, *SLC30A5*, and *DSTYK*. *SLC38A6* [sodium-coupled amino acid transporter-6 (SNAT-6)] is involved in the regulation of the placental glutamate–glutamine cycle, which has been associated to fetal growth (Wu et al., 2015; Simner et al., 2017; McIntyre et al., 2019). SLC30A5 encodes a zinc transporter-5 (*ZnT5*) protein which is localized at the apical membrane of the placental syncytiotrophoblast and believed to play an essential role in the transfer of Zn to fetus. *ZnT5* has also been shown to regulate ribosome biogenesis (Ogo et al., 2015). We recently demonstrated that placental mTOR regulates trophoblast nutrient transporters at the post-translational level. Specifically, mTOR functions as a positive regulator of amino acid transporter systems A and L (Rosario et al., 2013) and folate transporters (Rosario et al., 2016) by modulating the plasma membrane trafficking of specific transporter isoforms. These findings together with the demonstration in the current study that mTORC1 also regulates specific trophoblast nutrient transporters at the transcriptional level suggest that trophoblast mTOR is a master regulator of a range of placental nutrient transporters mediated by distinct molecular mechanisms.

We also observed a decrease in the expression of genes involved in redox regulation, such as *GPX1*, following mTORC1 inhibition in PHT cells. The expression of *CLPP*, an ATP-dependent peptidase in the inner mitochondrial membrane, which is involved in the activation of the mitochondrial protein unfolding response (Haynes et al., 2007), was also decreased in raptor silenced cells. We observed a significant reduction in the transcript levels of a number of mitochondrial ribosomal proteins in the 39S (*MRL18*, *MRL16*) and 28S (*MRPS34*) ribosome subunits in PHT cells following mTORC1 inhibition, which is in line with previous reports that mTORC1 controls the synthesis of mitochondrial ribosomal proteins in MCF7 cells (Morita et al., 2015).

One potential limitation of our study is that we used a single siRNA to silence raptor, which could be associated with off-target effects. However, BlastN analysis demonstrated that our raptor siRNA was not complementary to any other gene sequence. In addition, we have demonstrated that raptor silencing (mTORC1 inhibition) in PHT cells did not affect mTORC2 signaling (Rosario et al., 2013), suggesting high specificity in targeting mTORC1. Moreover, in unpublished studies, we used co-transfection of rictor siRNA (to inhibit mTORC2) and siRNA targeting DEPTOR (an endogenous inhibitor of both mTORC1 and 2) to specifically activate mTORC1 in PHT cells. Importantly, stimulation of mTORC1 activated PHT functions, including amino acid transport, that are inhibited by our raptor siRNA (unpublished). These observations strongly suggest that the raptor siRNA used specifically inhibits mTORC1 signaling and that significant off-target effects are unlikely.

## Conclusion

Mechanistic Target of Rapamycin Complex 1 signaling regulates the expression of trophoblast genes involved in ribosome and protein synthesis, mitochondrial function, lipid metabolism, nutrient transport, and angiogenesis, representing novel links between mTOR signaling and placental functions critical for normal fetal growth and development. Because placental mTOR signaling is inhibited in IUGR and activated in fetal overgrowth, we propose that regulation of the placental transcriptome by mTOR signaling directly contributes to altered placental function and fetal growth in common pregnancy complications.

## Data Availability Statement

The datasets presented in this study can be found in online repositories. The names of the repository/repositories and accession number(s) can be found in the article/Supplementary Material.

## Ethics Statement

The studies involving human participants were reviewed and approved by the University of Colorado. The patients/participants provided their written informed consent to participate in this study.

## Author Contributions

FR, LC, MG, TJ, and TP made contribution to conception and design of the experiments and performed collection, analysis, and interpretation of data. FR, TJ, and TP wrote the manuscript. All authors approved the final version of the manuscript.

## Conflict of Interest

The authors declare that the research was conducted in the absence of any commercial or financial relationships that could be construed as a potential conflict of interest.

## Footnotes

1 http://www.genome.jp/kegg/

2 https://www.qiagenbioinformatics.com/products/ingenuity-pathway-analysis

3 http://www.genome.jp/kegg/
