# Supplementary material for: mTORC1 Transcriptional Regulation of Ribosome Subunits, Protein Synthesis, and Molecular Transport in Primary Human Trophoblast Cells
Source: Front Cell Dev Biol. 2020 Nov 26;8:583801. doi: 10.3389/fcell.2020.583801 (PMC7726231; doi:10.3389/fcell.2020.583801)
Supplement: Supplementary file 1 [file Table_1.pdf]

mTORC1 transcriptional regulation of ribosome subunits, protein synthesis and molecular transport in primary human trophoblast cells

[1]Fredrick J Rosario, [1,2]Theresa L Powell, [3]Madhulika B Gupta, [4]Laura Cox, [1]Thomas Jansson

[1]Division of Reproductive Sciences, Department of OB/GYN University of Colorado Anschutz Medical Campus, Aurora, CO, USA; [2]Section of Neonatology, Department of Pediatrics, University of Colorado Anschutz Medical Campus, Aurora, CO, USA; [3]University of Western Ontario, London, Ontario, Canada; [4]Center for Precision Medicine, Department of Internal Medicine, Section of Molecular Medicine, Wake Forest School of Medicine, Winston-Salem, NC, USA.

Short Title: mTORC1 and the trophoblast transcriptome

Key words: Placenta, maternal-fetal exchange, human, nutrient sensor, energy metabolism, gene array

Corresponding author:

Fredrick J Rosario

Department of Obstetrics & Gynecology, 12700 East 19th Avenue, University of Colorado Anschutz Medical Campus, Aurora, Colorado 80045.

Office: 303 724 8857, Fax: 303 724 3512.

Email: FREDRICK.JOSEPH@CUANSCHUTZ.EDU

Supplemental Figure 1. Top-ranking networks for differences in gene expression in PHT cells with mTORC1 inhibition (raptor silencing) as compared to control cells. Genes are denoted by gene IDs, green indicates down regulated, red indicates upregulated, gray indicates no difference in gene expression in cells with mTORC1 inhibition as compared to control cells and white denotes the lack of quality signal on the array.

ACAA1, acetyl-CoA acyltransferase 1; ACAT1, acetyl-CoA acetyltransferase 1; ALDOC, aldolase C, fructose-bisphosphate; APBA3, amyloid beta (A4) precursor protein-binding, family A, member 3; APOC1, apolipoprotein C-I; APOC2, apolipoprotein C-II; APOE, apolipoprotein E; ARFGAP1, ADP-ribosylation factor GTPase activating protein 1; ARL6IP5, ADP-ribosylation-like factor 6 interacting protein 5; ASAH1, N-acylsphingosine amidohydrolase (acid ceramidase) 1; ATP2A2, ATPase, Ca++ transporting, cardiac muscle, slow twitch 2; ATP5G1, ATP synthase, H+ transporting, mitochondrial Fo complex, subunit C1 (subunit 9); ATP5J2, ATP synthase, H+ transporting, mitochondrial Fo complex, subunit F2; ATP5S, ATP synthase, H+ transporting, mitochondrial Fo complex, subunit s (factor B); B3GALNT2, beta-1,3-N-acetylgalactosaminyltransferase 2; BPGM, 2,3-bisphosphoglycerate mutase; CHMP2A, charged multivesicular body protein 2A; COLGALT1, collagen beta(1-O)galactosyltransferase 1; CPT1A, carnitine palmitoyltransferase 1A (liver); CRELD1, cysteine-rich with EGF-like domains 1; EFHD2, EF-hand domain family, member D2; FBP1, fructose-1,6-bisphosphatase 1; FBXO6, F-box protein 6; FGL1, fibrinogen-like 1; G6PD, glucose-6-phosphate dehydrogenase; GLA, galactosidase, alpha; GLRX3, glutaredoxin 3; GRN, granulin; HDL; HELZ2, helicase with zinc finger 2, transcriptional coactivator; HIGD1A, HIG1 hypoxia inducible domain family, member 1A; HK3, hexokinase 3 (white cell); HSPA1A/HSPA1B, heat shock 70kDa protein 1A; IFITM3, interferon induced transmembrane protein 3; ING2, inhibitor of growth family, member 2; ISG15, ISG15 ubiquitin-like modifier; KLHDC2, kelch domain containing 2; LYZ lysozyme; NEUROG3 neurogenin 3; NR4A1 nuclear receptor subfamily 4, group A, member 1; PBRM1, polybromo 1; PCBD1, pterin-4 alpha-carbinolamine dehydratase/dimerization cofactor of hepatocyte nuclear factor 1 alpha; PCYOX1, prenylcysteine oxidase 1 PDCD4, programmed cell death 4 (neoplastic transformation inhibitor); PPM1B, protein phosphatase, Mg2+/Mn2+ dependent, 1B; PPT1, palmitoyl-protein thioesterase 1; PSAP, prosaposin; PSEN1, presenilin 1 ; PTS, 6-pyruvoyltetrahydropterin synthase; REEP3, receptor accessory protein 3; RGS19, regulator of G-protein signaling 19; SAT2, spermidine/spermine N1-acetyltransferase family member 2; SLC27A1, solute carrier family 27 (fatty acid transporter), member 1; SMARCC1, SWI/SNF related, matrix associated, actin dependent regulator of chromatin, subfamily c, member 1; TCEB1, transcription elongation factor B (SIII), polypeptide 1 (15kDa, elongin C); TFAP2A, transcription factor AP-2 alpha (activating enhancer binding protein 2 alpha); TGM2, transglutaminase 2 (C polypeptide, protein-glutamine-gamma-glutamyltransferase); TMEM230, transmembrane protein 230; TMEM256, transmembrane protein 256; TMLHE, trimethyllysine hydroxylase, epsilon; TOP2A, topoisomerase (DNA) II alpha 170kDa; USP28, ubiquitin specific peptidase 28; VKORC1, vitamin K epoxide reductase complex, subunit 1; VPS29, vacuolar protein sorting 29 homolog (S. cerevisiae); WDYHV1, WDYHV motif containing 1; ZC3HAV1, zinc finger CCCH-type, antiviral 1.

Supplemental Figure 2. Top-ranking networks for differences in gene expression in PHT cells with mTORC1 inhibition (raptor silencing) as compared to control cells. Genes are denoted by gene IDs, green indicates down regulated, red indicates upregulated, gray indicates no difference in gene expression in cells with mTORC1 inhibition as compared to control cells and white denotes the lack of quality signal on the array.

ABHD14A, abhydrolase domain containing 14A; AGPAT2, 1-acylglycerol-3-phosphate O-acyltransferase 2; ALG2, ALG2, alpha-1,3/1,6-mannosyltransferase; APAF1, apoptotic peptidase activating factor 1; APIP, APAF1 interacting protein; ASNS, asparagine synthetase (glutamine-hydrolyzing); ATF3, activating transcription factor 3; CCL5, chemokine (C-C motif) ligand 5; CGA, glycoprotein hormones, alpha polypeptide; COMMD10, COMM domain containing 10; CPNE1, copine I; DBT, dihydrolipoamide branched chain transacylase E2; EIF3C/EIF3CL, eukaryotic translation initiation factor 3, subunit C; FABP4, fatty acid binding protein 4, adipocyte; FOSB, FBJ murine osteosarcoma viral oncogene homolog B; GPN3, GPN-loop GTPase 3; GPX1, glutathione peroxidase 1; HCST, hematopoietic cell signal transducer; ID2, inhibitor of DNA binding 2, dominant negative helix-loop-helix protein; IFNAR2, interferon (alpha, beta and omega) receptor 2; IL24, interleukin 24; IL6, interleukin 6 (interferon, beta 2); JUNB, jun B proto-oncogene; MINOS1, mitochondrial inner membrane organizing system 1; MRPL16, mitochondrial ribosomal protein L16; MRPL18, mitochondrial ribosomal protein L18; MRPS34, mitochondrial ribosomal protein S34;OASL, 2'-5'- oligoadenylate synthetase-like; PAFAH1B1, platelet-activating factor acetylhydrolase 1b, regulatory subunit 1 (45kDa); PBX3, pre-B-cell leukemia homeobox 3; PDIA4, protein disulfide isomerase family A, member 4;MSN, moesin; NFATC3, nuclear factor of activated T-cells, cytoplasmic, calcineurin-dependent 3; NFKB2, nuclear factor of kappa light polypeptide gene enhancer in B-cells 2 (p49/p100); NUDCD2, NudC domain containing 2; POLR2G, polymerase (RNA) II (DNA directed) polypeptide G; PRKRA, protein kinase, interferon-inducible double stranded RNA dependent activator; RAP1GDS1, RAP1, GTP-GDP dissociation stimulator 1; RPL10A, ribosomal protein L10a;RPL12, ribosomal protein L12; RPL18A, ribosomal protein L18a; RPL22, ribosomal protein L22; RPL26, ribosomal protein L26; RPL30, ribosomal protein L30; RPL31, ribosomal protein L31; RPL38, ribosomal protein L38;RPS10, ribosomal protein S10; RPS12, ribosomal protein S12; RPS14, ribosomal protein S14; RPS15A, ribosomal protein S15a; RPS20, ribosomal protein S20; RPS27, ribosomal protein S27; RPS6, ribosomal protein S6;SCPEP1, serine carboxypeptidase 1; SERBP1, SERPINE1 mRNA binding protein 1;SERPINB2, serpin peptidase inhibitor, clade B (ovalbumin), member 2; TBCB, tubulin folding cofactor B;TREM2, triggering receptor expressed on myeloid cells 2; TYROBP, TYRO protein tyrosine kinase binding protein; UBA52, ubiquitin A-52 residue ribosomal protein fusion product 1; UBE2O, ubiquitin-conjugating enzyme E2O; YBX1, Y box binding protein 1; ZBTB18, zinc finger and BTB domain containing 18; Creb; Ribosomal 40s subunit; RNA polymerase II; IFN Beta; Vegf; TH2 Cytokine.

Supplemental Figure 3. Top-ranking networks for differences in gene expression in PHT cells with mTORC1 inhibition (raptor silencing) as compared to control cells. Genes are denoted by gene IDs, green indicates down regulated, red indicates upregulated, gray indicates no difference in gene expression in cells with mTORC1 inhibition as compared to control cells and white denotes the lack of quality signal on the array.

ADVL, acyl-CoA dehydrogenase, very long chain; ACO1, aconitase 1, soluble; ALAS2, aminolevulinate, delta-, synthase 2; C1QB, complement component 1, q subcomponent, B chain; C1QC, complement component 1, q subcomponent, C chain; C1S, complement component 1, s subcomponent; C20orf24, chromosome 20 open reading frame 24; CBX5, chromobox homolog 5; CD52, CD52 molecule; CLPP, ClpP caseinolytic peptidase, ATP-dependent, proteolytic subunit homolog (E. coli); COX17, COX17 cytochrome c oxidase copper chaperone; COX6C, cytochrome c oxidase subunit Vic; COX7C, cytochrome c oxidase subunit VIIc; CREG1, cellular repressor of E1A-stimulated genes1; CYB5A, cytochrome b5 type A (microsomal); DECR1, 2,4-dienoyl CoA reductase 1, mitochondrial; DNASE2, deoxyribonuclease II, lysosomal; E4F1, E4F transcription factor 1; EZH2, enhancer of zeste homolog 2 (Drosophila); FAM173A, family with sequence similarity 173, member A; HDAC9, histone deacetylase 9; HIST1H2AB/HIST1H2AE, histone cluster 1, H2ae; HIST1H2AM (includes others), histone cluster 1, H2ag; HIST1H2BJ/HIST1H2BK, histone cluster 1, H2bk; HOPX, HOP homeobox; IK, IK cytokine, down-regulator of HLA II; ING3, inhibitor of growth family, member 3; IVNS1ABP, influenza virus NS1A binding protein; LAMP2, lysosomal-associated membrane protein 2; LGALS1, lectin, galactoside-binding, soluble, 1; MAX, MYC associated factor X; MNDA, myeloid cell nuclear differentiation antigen; MORF4L1, mortality factor 4 like 1; NDUFA1, NADH dehydrogenase (ubiquinone) 1 alpha subcomplex, 1, 7.5kDa; NDUFB6, NADH dehydrogenase (ubiquinone) 1 beta subcomplex, 6, 17kDa; NDUFB8, NADH dehydrogenase (ubiquinone) 1 beta subcomplex, 8, 19kDa; NDUFS7, NADH dehydrogenase (ubiquinone) Fe-S protein 7, 20kDa (NADH-coenzyme Q reductase);NUSAP1, nucleolar and spindle associated protein 1; PAF1, Paf1, RNA polymerase II associated factor, homolog (S. cerevisiae); PCNT, pericentrin; PDRG1, p53 and DNA-damage regulated 1; PFN2, profilin 2; PSMG1, proteasome (prosome, macropain) assembly chaperone 1; PTPLAD1, protein tyrosine phosphatase-like A domain containing 1; RPN1, ribophorin I; RQCD1, RCD1 required for cell differentiation1 homolog (S. pombe); RUNX3, runt-related transcription factor 3; RUVBL2, RuvB-like 2 (E. coli); SERINC3, serine incorporator 3; SMAD6, SMAD family member 6; SPTLC1, serine palmitoyltransferase, long chain base subunit 1; ST3GAL5, ST3 beta-galactoside alpha-2,3-sialyltransferase 5;SUSD2, sushi domain containing 2;TLE6, transducin-like enhancer of split 6 (E(sp1) homolog, Drosophila); TSPAN17, tetraspanin 17; WDR92, WD repeat domain 92; C1q; Calmodulin; CD3, cytochrome-c oxidase; E2f, Hdac; Mitochondrial complex 1; Rb; Secretase gamma; TIP60.

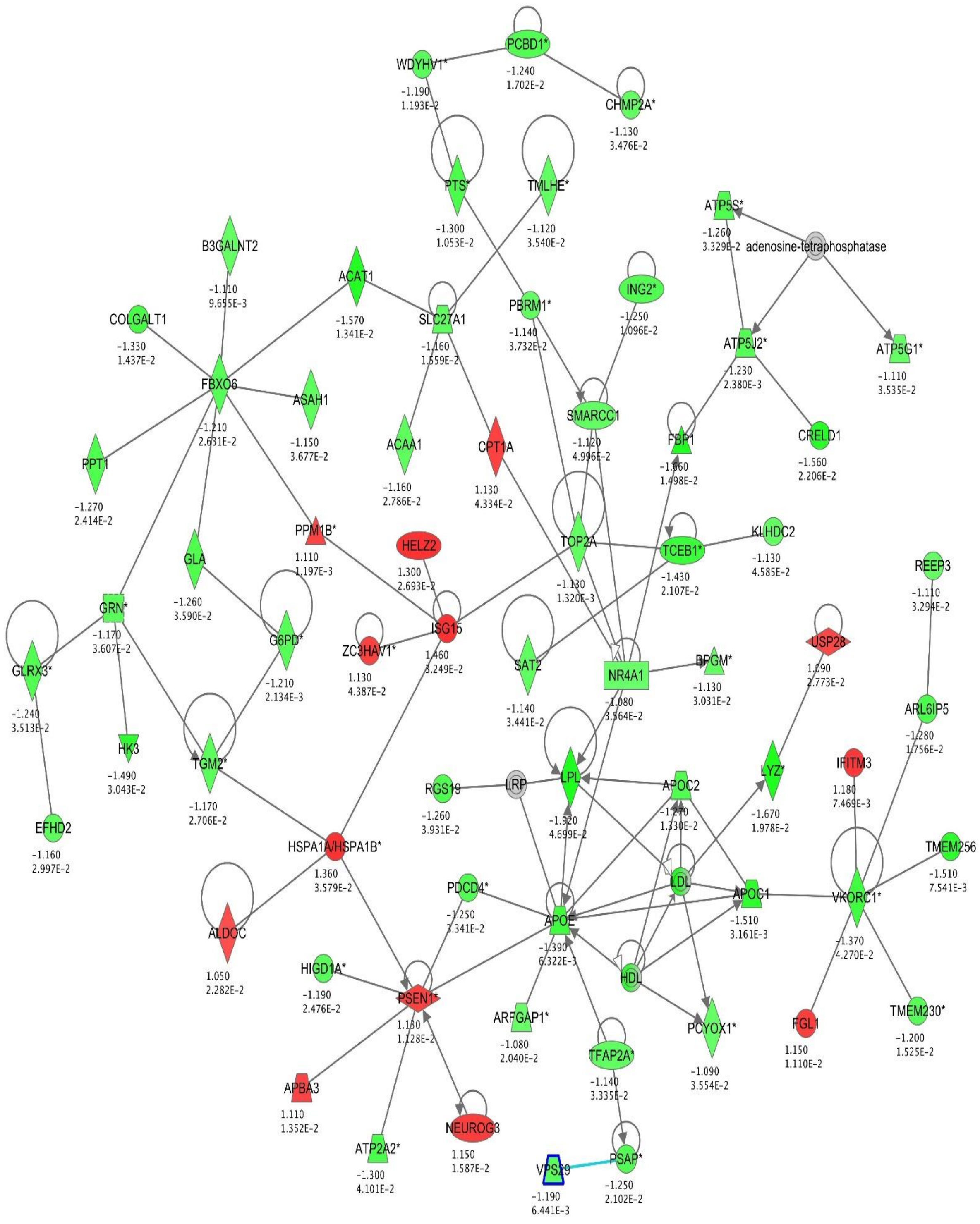

© 2000-2013 Ingenuity Systems, Inc. All rights reserved.

Supplemental Figure 1

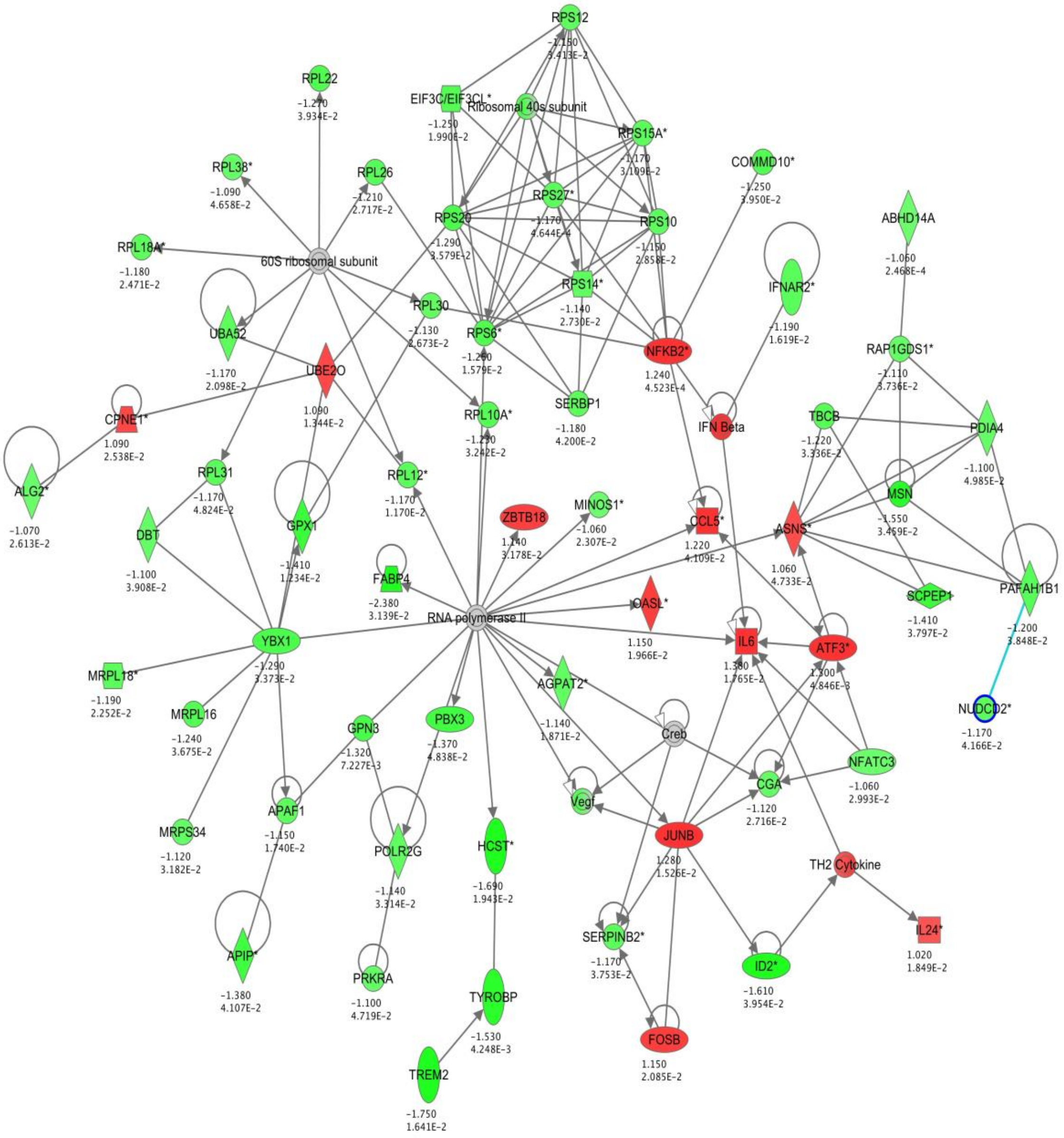

© 2000-2013 Ingenuity Systems, Inc. All rights reserved.

Supplemental Figure 2

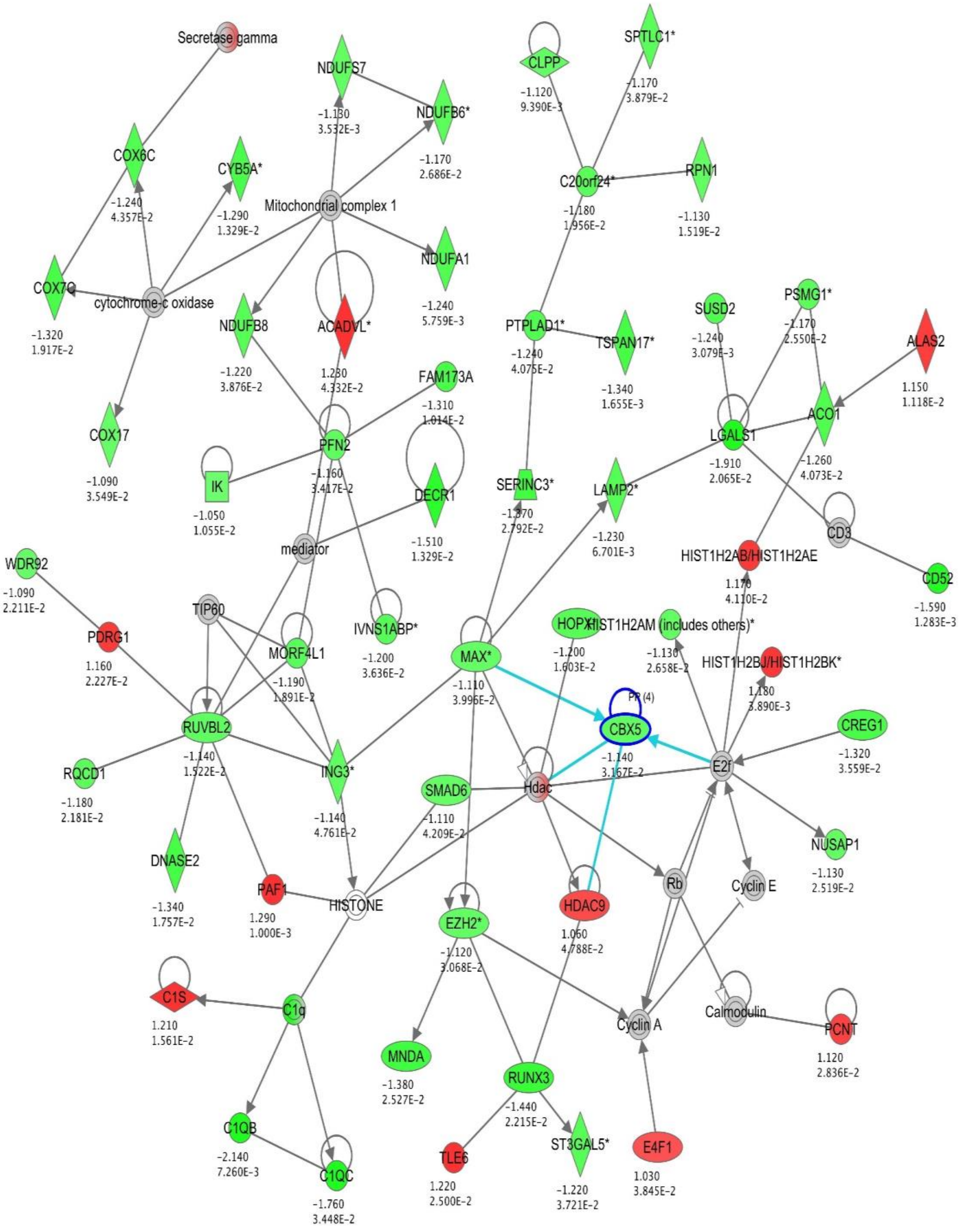

© 2000-2013 Ingenuity Systems, Inc. All rights reserved.

Supplemental Figure 3

Supplemental Table 1:List of Differentially Expressed Genes in mTORC1 inhbited PHT cells:.

Gene Name	Gene ID	Scramble siRNA (Expression)	Scramble siRNA (S.E.M)	Raptor siRNA (Expression)	Raptor siRNA (S.E.M)	Ratio	Direction	p-value	Gene Identifier	Other ID	UG Cluster	Locus Link	Chromosome
(clone 1NIB-4) normalized cDNA library sequence	-	0.619874	0.052473	0.331981	0.08068	1.22	Down	0.02428	AK090694	ILMN_1911721	Hs.224794	-	5
2-5-oligoadenylate synthetase-like	OASL	0.728669	0.028258	0.934509	0.07142	1.15	Up	0.03654	NM_003733	ILMN_1681721	Hs.118633	8638	12
2,4-dienoyl CoA reductase 1, mitochondrial	DECR1	2.804618	0.199161	2.214459	0.1114	1.51	Down	0.04142	NM_001359	ILMN_1720838	Hs.492212	1666	8
5-nucleotidase domain containing 1	NT5DC1	1.062444	0.058564	0.807615	0.04802	1.19	Down	0.01514	NM_152729	ILMN_1680673	Hs.719198	2E+05	6
absent in melanoma 1-like	-	0.126218	0.016436	0.284428	0.0141	1.12	Up	0.00034	NM_017977	ILMN_1776916	-	55057	-
Acetyl-Coenzyme A acetyltransferase 1	ACAT1	2.069091	0.138126	1.41884	0.03819	1.57	Down	0.00394	NM_000019	ILMN_1800008	Hs.232375	38	11
Aconitase 1, soluble	ACO1	3.266887	0.098274	2.934761	0.04841	1.26	Down	0.02304	NM_002197	ILMN_1750800	Hs.567229	48	9
Activating transcription factor 3	ATF3	0.007596	0.044397	0.388633	0.07049	1.3	Up	0.00379	NM_001030287	ILMN_1661109	Hs.460	467	1
Acyl-CoA thioesterase 4	ACOT4	1.94035	0.061867	1.639959	0.09249	1.23	Down	0.0356	NM_152331	ILMN_1764321	Hs.49433	1E+05	14
Acyl-CoA thioesterase 9	ACOT9	1.555825	0.087987	1.253257	0.08579	1.23	Down	0.04898	NM_001033583	ILMN_1658995	Hs.298885	23597	X
Acyl-CoA thioesterase 9	ACOT9	0.930864	0.062376	0.750588	0.01372	1.13	Down	0.03025	NM_001037171	ILMN_2367070	Hs.298885	23597	X
Adaptor-related protein complex 1, sigma 2 subunit	AP1S2	2.202085	0.258445	1.183908	0.07448	2.03	Down	0.00912	NM_003916	ILMN_2120273	Hs.656471	8905	X
Adaptor-related protein complex 1, sigma 2 subunit	AP1S2	2.342556	0.239336	1.470579	0.11621	1.83	Down	0.01688	NM_003916	ILMN_1766411	Hs.656471	8905	X
Adenine phosphoribosyltransferase	APRT	1.55184	0.055493	1.299498	0.05074	1.19	Down	0.01531	NM_000485	ILMN_1726410	Hs.28914	353	16
Adenylate cyclase activating polypeptide 1 (pituitary) receptor type I	ADCYAP1R1	0.265506	0.046645	0.398716	0.02746	1.1	Up	0.04905	NM_001118	ILMN_1711736	Hs.377783	117	7
ADP-ribosylation-like factor 6 interacting protein 5	ARL6IP5	4.76261	0.073091	4.401288	0.06716	1.28	Down	0.01083	NM_006407	ILMN_1769810	Hs.716493	10550	3
Adrenomedullin	ADM	7.2784	0.036138	7.446476	0.04917	1.12	Up	0.0331	NM_001124	ILMN_1708934	Hs.441047	133	11
Aggrecan	ACAN	0.348399	0.028482	0.436015	0.01171	1.06	Up	0.02936	NM_001135	ILMN_1777182	Hs.2159	176	15
Alanyl-tRNA synthetase domain containing 1	AARSD1	0.835637	0.071206	0.553584	0.07737	1.22	Down	0.03642	NM_025267	ILMN_1700461	Hs.317403	80755	17
Aldehyde dehydrogenase 1 family, member A1	ALDH1A1	3.180163	0.250506	1.910224	0.14424	2.41	Down	0.0046	NM_000689	ILMN_1709348	Hs.76392	216	9
Aldehyde dehydrogenase 1 family, member A1	ALDH1A1	2.650712	0.272071	1.458366	0.08446	2.29	Down	0.00578	NM_000689	ILMN_2096372	Hs.76392	216	9
Aldo-keto reductase family 1, member A1 (aldehyde reductase)	AKR1A1	4.070223	0.123556	3.621093	0.08917	1.37	Down	0.02569	NM_153326	ILMN_2380771	Hs.654435	10327	1
Aldo-keto reductase family 1, member A1 (aldehyde reductase)	AKR1A1	3.673091	0.071814	3.263528	0.06824	1.33	Down	0.00612	NM_006066	ILMN_1728047	Hs.654435	10327	1
Aminolevulinate, delta-, synthase 2	ALAS2	0.436491	0.009287	0.63331	0.0436	1.15	Up	0.00449	NM_001037967	ILMN_1708323	Hs.522666	212	X
Annexin A4	ANXA4	1.01835	0.111508	0.703555	0.0246	1.24	Down	0.03299	NM_001153	ILMN_1711408	Hs.422986	307	2
Anoctamin 2	ANO2	1.142471	0.065724	1.356416	0.03253	1.16	Up	0.02672	NM_020373	ILMN_3248094	Hs.148970	57101	12
Antizyme inhibitor 1	AZIN1	5.227028	0.10269	4.883257	0.06311	1.27	Down	0.0291	NM_015878	ILMN_1656682	Hs.459106	51582	8
AP2 associated kinase 1	AAK1	1.16272	0.09795	0.837667	0.06537	1.25	Down	0.03284	NM_014911	ILMN_1688755	Hs.468878	22848	2
APAF1 interacting protein	APIP	1.910929	0.073256	1.445727	0.13074	1.38	Down	0.02101	NM_015957	ILMN_1793598	Hs.447794	51074	11
Apolipoprotein B mRNA editing enzyme, catalytic	APOBEC3C	0.743577	0.017776	0.871864	0.03038	1.09	Up	0.01077	NM_014508	ILMN_1675684	Hs.441124	27350	22
Apolipoprotein B mRNA editing enzyme, catalytic	APOBEC3G	0.284239	0.073125	0.062476	0.02196	1.17	Down	0.02717	NM_021822	ILMN_1802106	Hs.660143	60489	22
Apolipoprotein C-II	APOC2	0.810274	0.088965	0.4601	0.08041	1.27	Down	0.02663	NM_000483	ILMN_1802923	Hs.75615	344	19
Apoptosis-associated tyrosine kinase	AATK	0.245628	0.038615	0.097053	0.02969	1.11	Down	0.0225	NM_001080395	ILMN_1687609	Hs.514575	9625	17
Apoptotic peptidase activating factor 1	APAF1	0.582244	0.033949	0.381555	0.03227	1.15	Down	0.00518	NM_013229	ILMN_1659463	Hs.552567	317	12
Asparagine-linked glycosylation 8, alpha-1,3-glucosyltransferase homolog (S. cerevisiae)	ALG8	2.650191	0.081048	2.336501	0.08038	1.24	Down	0.03337	NM_001007027	ILMN_2365686	Hs.503368	79053	11
Asparagine-linked glycosylation 8, alpha-1,3-glucosyltransferase homolog (S. cerevisiae)	ALG8	3.118967	0.047238	2.841695	0.05953	1.21	Down	0.01072	NM_024079	ILMN_1685413	Hs.503368	79053	11
Asparaginyl-tRNA synthetase	NARS	4.17704	0.053775	3.888216	0.09622	1.22	Down	0.03957	NM_004539	ILMN_1732216	Hs.465224	4677	18
ATG7 autophagy related 7 homolog (S. cerevisiae)	ATG7	2.228636	0.094359	1.938577	0.05362	1.22	Down	0.0369	NM_006395	ILMN_1790978	Hs.716466	10533	3
ATP synthase, H+ transporting, mitochondrial F0 complex, subunit C2 (subunit 9)	ATP5G2	3.918491	0.077102	3.655544	0.0587	1.2	Down	0.03494	NM_005176	ILMN_1660577	Hs.524464	517	12
complex, epsilon subunit pseudogene 2 (ATP5EP2), transcript variant 6, non-coding RNA.	-	5.568734	0.130468	5.138707	0.10237	1.35	Down	0.04104	NR_002162	ILMN_2225887	-	-	-
ATPase, Ca++ transporting, cardiac muscle, slow	ATP2A2	2.829469	0.125139	2.454797	0.04697	1.3	Down	0.03104	NM_170665	ILMN_1815666	Hs.506759	488	12
ATPase, Ca++ transporting, type 2C, member 1	ATP2C1	0.459059	0.063096	0.181838	0.09046	1.21	Down	0.04568	NM_001001485	ILMN_1758784	Hs.584884	27032	3
ATPase, H+ transporting, lysosomal 9kDa, V0	ATP6V0E1	6.043373	0.097016	5.78543	0.03545	1.2	Down	0.0467	NM_003945	ILMN_2071937	Hs.484188	8992	5
ATPase, H+ transporting, lysosomal 9kDa, V0	ATP6V0E1	5.000798	0.062427	4.795512	0.04602	1.15	Down	0.03818	NM_003945	ILMN_1715635	Hs.484188	8992	5
ATPase, Na+/K+ transporting, beta 1 polypeptide	ATP1B1	3.029619	0.201089	2.322735	0.19538	1.63	Down	0.04521	NM_001001787	ILMN_2407824	Hs.291196	481	1
B-cell receptor-associated protein 31	BCAP31	5.389066	0.078387	5.143	0.02243	1.19	Down	0.02346	NM_005745	ILMN_1812403	Hs.522817	10134	X
Basic leucine zipper transcription factor, ATF-like	BATF	0.294186	0.060052	0.099549	0.02364	1.14	Down	0.02352	NM_006399	ILMN_1668822	Hs.509964	10538	14

Beta-1,3-N-acetylgalactosaminyltransferase 2	B3GALNT2	0.317541	0.025856	0.162837	0.02757	1.11	Down	0.00641	NM_152490	ILMN_1780188	Hs.716571	1E+05	1
Biliverdin reductase A	BLVRA	2.392729	0.176836	1.696031	0.07331	1.62	Down	0.01084	NM_000712	ILMN_1691436	Hs.488143	644	7
Bradykinin receptor B1	BDKRB1	0.379628	0.034984	0.542613	0.05434	1.12	Up	0.04516	NM_000710	ILMN_1734611	Hs.525572	623	14
BRI3 binding protein	BRI3BP	0.750512	0.066144	0.513271	0.05434	1.18	Down	0.03236	NM_080626	ILMN_1803570	Hs.596464	1E+05	12
Bridging integrator 1	BIN1	0.411428	0.039511	0.183966	0.07356	1.17	Down	0.03445	NM_139343	ILMN_2309245	Hs.193163	274	2
Bromodomain containing 7	BRD7	3.275267	0.01223	3.039279	0.08811	1.18	Down	0.03788	NM_013263	ILMN_2082810	Hs.437894	29117	16
C1GALT1-specific chaperone 1	C1GALT1C1	2.672254	0.033395	2.426629	0.08902	1.19	Down	0.04158	NM_152692	ILMN_1751234	Hs.643920	29071	X
C1GALT1-specific chaperone 1	C1GALT1C1	1.244736	0.044408	1.093123	0.0411	1.11	Down	0.04618	NM_001011551	ILMN_2401730	Hs.643920	29071	X
Cadherin 15, type 1, M-cadherin (myotubule)	CDH15	0.531057	0.047741	0.718019	0.04828	1.14	Up	0.03314	NM_004933	ILMN_1784036	Hs.148090	1013	16
Cancer/testis antigen family 47, member A1	CT47A1	0.501093	0.026204	0.680818	0.0597	1.13	Up	0.033	NM_001080146	ILMN_1673271	Hs.673190	7E+05	X
Canopy 4 homolog (zebrafish)	CNPY4	1.284163	0.097579	0.944999	0.02072	1.27	Down	0.0145	NM_152755	ILMN_2252408	Hs.632293	2E+05	7
CAP, adenylate cyclase-associated protein 1	CAP1	5.751871	0.07633	5.522933	0.04051	1.17	Down	0.03806	NM_006367	ILMN_1797604	Hs.370581	10487	1
Carbonic anhydrase II	CA2	1.499619	0.200583	0.849407	0.12774	1.57	Down	0.034	NM_000067	ILMN_2199439	Hs.155097	760	8
Carboxymethylenebutenolidase homolog	CMBL	0.387543	0.061084	0.179778	0.05603	1.15	Down	0.04611	NM_138809	ILMN_1709634	Hs.192586	1E+05	5
Carboxypeptidase M	CPM	0.556101	0.040527	0.393175	0.00924	1.12	Down	0.00781	NM_001874	ILMN_1698313	Hs.654387	1368	12
Carboxypeptidase Z	CPZ	1.378554	0.060193	1.66886	0.03264	1.22	Up	0.00544	NM_001014447	ILMN_1705258	Hs.78068	8532	4
casein kinase 2, alpha 1 polypeptide pseudogene	-	0.875158	0.04151	1.012046	0.01267	1.1	Up	0.01971	NR_002207	ILMN_1746375	-	3E+05	-
CCAAT/enhancer binding protein (C/EBP), alpha	CEBPA	4.689297	0.084264	4.321644	0.10765	1.29	Down	0.03608	NM_004364	ILMN_1715715	Hs.699463	1050	19
CD68 molecule	CD68	2.249059	0.154538	1.688668	0.14809	1.47	Down	0.03969	NM_001251	ILMN_2267914	Hs.647419	968	17
CDKN1A interacting zinc finger protein 1	CIZ1	0.228827	0.020427	0.315003	0.02355	1.06	Up	0.03266	NM_012127	ILMN_1724659	Hs.212395	25792	9
CDNA FLJ39749 fis, clone SMINT2017599	-	0.539223	0.0812	0.798726	0.05998	1.2	Up	0.0423	CR603272	ILMN_1847965	Hs.250648	-	3
Cell adhesion molecule 4	CADM4	0.43541	0.019535	0.586101	0.02991	1.11	Up	0.00557	NM_145296	ILMN_1812096	Hs.370984	2E+05	19
Cell cycle exit and neuronal differentiation 1	CEND1	0.616316	0.01574	0.772998	0.05727	1.11	Up	0.03865	NM_016564	ILMN_1720482	Hs.22140	51286	11
Cell division cycle associated 7-like	CDCA7L	0.434036	0.016917	0.193903	0.03089	1.18	Down	0.00049	NM_018719	ILMN_2230683	Hs.520245	55536	7
Centromere protein L	CENPL	0.414365	0.018257	0.351062	0.01167	1.04	Down	0.02658	NM_033319	ILMN_1742779	Hs.531856	91687	1
Chemokine (C-C motif) receptor-like 1	CCRL1	0.12601	0.063023	0.337263	0.05104	1.16	Up	0.04039	NM_016557	ILMN_1773992	Hs.719388	51554	3
Cholecystokinin A receptor	CCKAR	0.262089	0.050843	0.119537	0.00702	1.1	Down	0.03211	NM_000730	ILMN_1656330	Hs.129	886	4
Chondroitin sulfate synthase 1	CHSY1	5.374733	0.093018	5.134547	0.02118	1.18	Down	0.04543	NM_014918	ILMN_1791576	Hs.110488	22856	15
Chromatin modifying protein 2A	CHMP2A	1.186383	0.025867	1.009048	0.0288	1.13	Down	0.00377	NM_198426	ILMN_1762932	Hs.12107	27243	19
chromosome 1 open reading frame 194	-	0.654645	0.022817	0.878022	0.02046	1.17	Up	0.00034	XM_376965	ILMN_1773175	-	1E+05	-
Chromosome 10 open reading frame 57	C10orf57	2.174058	0.062033	1.866301	0.06206	1.24	Down	0.01271	NM_025125	ILMN_1672717	Hs.169982	80195	10
Chromosome 10 open reading frame 90	C10orf90	0.726003	0.061893	0.519308	0.04496	1.15	Down	0.03549	NM_001004298	ILMN_1680367	Hs.587663	1E+05	10
Chromosome 11 open reading frame 51	C11orf51	1.108161	0.079487	0.856842	0.05583	1.19	Down	0.04136	NM_014042	ILMN_1726104	Hs.38044	25906	11
Chromosome 11 open reading frame 59	C11orf59	4.434934	0.058557	4.255203	0.02706	1.13	Down	0.03173	NM_017907	ILMN_1815878	Hs.530753	55004	11
Chromosome 11 open reading frame 67	C11orf67	1.280214	0.085132	0.986092	0.0425	1.23	Down	0.02136	NM_024684	ILMN_1779163	Hs.503357	28971	11
Chromosome 12 open reading frame 48	C12orf48	0.381372	0.02286	0.165077	0.04126	1.16	Down	0.00375	NM_017915	ILMN_1727055	Hs.330663	55010	12
Chromosome 12 open reading frame 73	C12orf73	0.346718	0.034811	0.502689	0.03528	1.11	Up	0.01989	NM_001135570	ILMN_3241041	Hs.42547	7E+05	12
Chromosome 13 open reading frame 34	C13orf34	0.477913	0.037348	0.357656	0.02934	1.09	Down	0.04455	NM_024808	ILMN_1761486	Hs.714340	79866	13
Chromosome 15 open reading frame 52	C15orf52	0.460348	0.054894	0.178806	0.06757	1.22	Down	0.01782	NM_207380	ILMN_1775330	Hs.32433	4E+05	15
Chromosome 16 open reading frame 46	C16orf46	0.279737	0.020977	0.349057	0.01886	1.05	Up	0.04929	NM_152337	ILMN_1667665	Hs.658684	1E+05	16
Chromosome 16 open reading frame 70	C16orf70	1.066365	0.036785	1.260827	0.04723	1.14	Up	0.0175	NM_025187	ILMN_1711703	Hs.513666	80262	16
Chromosome 17 open reading frame 61	C17orf61	2.260476	0.188321	1.666599	0.12865	1.51	Down	0.04045	NM_152766	ILMN_2201533	Hs.534591	3E+05	17
Chromosome 17 open reading frame 95	C17orf95	1.668396	0.039513	1.427072	0.06303	1.18	Down	0.0176	NM_001080510	ILMN_2382724	Hs.74655	1E+05	17
Chromosome 18 open reading frame 21	C18orf21	2.010544	0.111763	1.580007	0.06425	1.35	Down	0.01562	NM_031446	ILMN_1805998	Hs.37883	83608	18
chromosome 19 open reading frame 23	-	0.752962	0.041365	0.90076	0.03567	1.11	Up	0.0353	NM_152480	ILMN_1657148	-	1E+05	-
chromosome 19 open reading frame 55	-	0.45423	0.043724	0.693986	0.03257	1.18	Up	0.00458	NM_144692	ILMN_1692947	-	1E+05	-
Chromosome 2 open reading frame 30	C2orf30	4.022036	0.092167	3.708729	0.04682	1.24	Down	0.02308	NM_015701	ILMN_1724376	Hs.438336	27248	2
Chromosome 2 open reading frame 54	C2orf54	0.154607	0.060431	0.390454	0.06971	1.18	Up	0.04311	NM_001085437	ILMN_1665471	Hs.193745	79919	2
Chromosome 20 open reading frame 24	C20orf24	5.882157	0.083878	5.63816	0.03657	1.18	Down	0.0372	NM_018840	ILMN_2387599	Hs.584985	55969	20
Chromosome 21 open reading frame 33	C21orf33	0.98477	0.058424	0.684087	0.09959	1.23	Down	0.04043	NM_004649	ILMN_1682812	Hs.413482	8209	21
Chromosome 5 open reading frame 13	C5orf13	0.946688	0.157091	0.499599	0.05777	1.36	Down	0.03697	NM_004772	ILMN_1680738	Hs.36053	9315	5
Chromosome 6 open reading frame 173	C6orf173	0.63663	0.144193	0.20062	0.04713	1.35	Down	0.02827	NM_001012507	ILMN_1763907	Hs.486401	4E+05	6
Chromosome 6 open reading frame 227	C6orf227	0.238015	0.022779	0.35282	0.03381	1.08	Up	0.03051	NM_207497	ILMN_1717726	Hs.520075	4E+05	6
Chromosome 9 open reading frame 24	C9orf24	0.173229	0.016783	0.309437	0.04352	1.1	Up	0.02662	NM_147169	ILMN_1763695	Hs.50334	84688	9
Chromosome 9 open reading frame 80	C9orf80	1.331989	0.068146	1.1149	0.05404	1.16	Down	0.04678	NM_021218	ILMN_1688621	Hs.658575	58493	9
Chymotrypsin C (caldecrin)	CTRC	0.100668	0.054022	0.300401	0.04804	1.15	Up	0.03273	NM_007272	ILMN_1748730	Hs.631869	11330	1
CKLF-like MARVEL transmembrane domain	CMTM4	2.336165	0.053207	1.94232	0.09162	1.31	Down	0.00988	NM_181521	ILMN_1815319	Hs.643961	1E+05	16
CKLF-like MARVEL transmembrane domain	CMTM4	0.887835	0.075512	0.666109	0.01395	1.17	Down	0.02779	NM_181521	ILMN_1762718	Hs.643961	1E+05	16
Clone TESTIS-609 mRNA sequence	-	0.439138	0.034136	0.576971	0.02357	1.1	Up	0.01595	AY726563	ILMN_1886515	Hs.660574	-	11
Coiled-coil and C2 domain containing 1A	CC2D1A	1.19704	0.062222	1.404664	0.02875	1.15	Up	0.02312	NM_017721	ILMN_1701127	Hs.269592	54862	19
Coiled-coil domain containing 104	CCDC104	0.396083	0.070217	0.186364	0.04451	1.16	Down	0.04513	NM_080667	ILMN_1792456	Hs.264208	1E+05	2
Coiled-coil domain containing 106	CCDC106	1.107515	0.040524	0.922829	0.04464	1.14	Down	0.02213	NM_013301	ILMN_1682567	Hs.82482	29903	19

Coiled-coil domain containing 130	CCDC130	3.053226	0.06892	3.298912	0.05858	1.19	Up	0.03482	NM_030818	ILMN_1758633	Hs.24998	81576	19
Coiled-coil domain containing 52	CCDC52	0.413769	0.028599	0.591785	0.03279	1.13	Up	0.00642	NM_144718	ILMN_1742380	Hs.477144	2E+05	3
Coiled-coil domain containing 90B	CCDC90B	3.762425	0.108229	3.409436	0.03766	1.28	Down	0.02165	NM_021825	ILMN_2232166	Hs.368866	60492	11
Coiled-coil-helix-coiled-coil-helix domain containing	CHCHD5	1.708253	0.097164	1.445339	0.01311	1.2	Down	0.03646	NM_032309	ILMN_1797530	Hs.375707	84269	2
Collagen, type IV, alpha 3 (Goodpasture antigen)	COL4A3BP	0.625068	0.038157	0.802732	0.01805	1.13	Up	0.00563	NM_005713	ILMN_2292123	Hs.270437	10087	5
COMM domain containing 9	COMMD9	2.072375	0.080459	1.789814	0.05863	1.22	Down	0.02963	NM_014186	ILMN_1808821	Hs.279836	29099	11
Complement component (3b/4b) receptor 1	CR1	0.390908	0.015116	0.648786	0.09275	1.2	Up	0.03354	NM_000573	ILMN_2388112	Hs.334019	1378	1
Complexin 2	CPLX2	0.435229	0.04499	0.572445	0.03122	1.1	Up	0.04618	NM_001008220	ILMN_1669382	Hs.193235	10814	5
CSRP2 binding protein	CSRP2BP	2.402603	0.079739	2.08939	0.04353	1.24	Down	0.01367	NM_020536	ILMN_1690386	Hs.488051	57325	20
CTD (carboxy-terminal domain, RNA polymerase II, polypeptide A) small phosphatase like 2	CTDSPL2	0.793718	0.035505	0.588891	0.07555	1.15	Down	0.04954	NM_016396	ILMN_2077758	Hs.497967	51496	15
Cyclic nucleotide gated channel beta 1	CNGB1	0.23848	0.013743	0.112287	0.03889	1.09	Down	0.02223	NM_001297	ILMN_1702383	Hs.147062	1258	16
Cysteine-rich with EGF-like domains 1	CRELD1	2.132754	0.096437	1.487612	0.09201	1.56	Down	0.00288	NM_001031717	ILMN_1739558	Hs.9383	78987	3
Cytochrome b5 reductase 3	CYB5R3	2.277382	0.024741	2.163591	0.0339	1.08	Down	0.03504	NM_000398	ILMN_1740441	Hs.700572	1727	22
Cytochrome c oxidase subunit VIc	COX6C	5.620381	0.051188	5.305934	0.0497	1.24	Down	0.00453	NM_004374	ILMN_1654151	Hs.351875	1345	8
Cytochrome c oxidase subunit VIIb	COX7B	3.741089	0.148256	3.220164	0.08761	1.43	Down	0.02325	NM_001866	ILMN_2184049	Hs.522699	1349	X
Cytochrome c oxidase subunit VIIc	COX7C	6.043491	0.086526	5.647577	0.08418	1.32	Down	0.01683	NM_001867	ILMN_1798189	Hs.430075	1350	5
Cytochrome P450, family 19, subfamily A,	CYP19A1	6.007631	0.11405	5.584039	0.12118	1.34	Down	0.04375	NM_031226	ILMN_1699139	Hs.260074	1588	15
Cytochrome P450, family 4, subfamily A,	CYP4A11	0.713849	0.028258	0.868143	0.05427	1.11	Up	0.04517	NM_000778	ILMN_1735816	Hs.714775	1579	1
DAZ interacting protein 3, zinc finger	DZIP3	0.898254	0.037267	1.144907	0.02604	1.19	Up	0.00162	NM_014648	ILMN_1763200	Hs.409210	9666	3
DCP1 decapping enzyme homolog B (S.	DCP1B	0.558098	0.031608	0.726383	0.03135	1.12	Up	0.00918	NM_152640	ILMN_1743992	Hs.130934	2E+05	12
Degenerative spermatocyte homolog 1, lipid	DEGS1	3.606804	0.157322	3.168569	0.06161	1.35	Down	0.041	NM_003676	ILMN_1667430	Hs.299878	8560	1
Dehydrogenase/reductase (SDR family) member 7	DHRS7	3.536276	0.148867	3.120721	0.07681	1.33	Down	0.04776	NM_016029	ILMN_1807455	Hs.59719	51635	14
DEP domain containing 5	DEPDC5	0.32008	0.021899	0.454348	0.03531	1.1	Up	0.01787	NM_001007188	ILMN_1800711	Hs.435022	9681	22
DET1 and DDB1 associated 1	DDA1	4.828932	0.059902	4.648717	0.04098	1.13	Down	0.04761	NM_024050	ILMN_1694530	Hs.706814	79016	19
Diazepam binding inhibitor (GABA receptor modulator, acyl-Coenzyme A binding protein)	DBI	2.464991	0.100952	1.694857	0.1224	1.71	Down	0.00284	NM_020548	ILMN_1755926	Hs.78888	1622	2
Diazepam binding inhibitor (GABA receptor modulator, acyl-Coenzyme A binding protein)	DBI	4.74159	0.125031	4.066818	0.13276	1.6	Down	0.01009	NM_001079863	ILMN_2305544	Hs.78888	1622	2
DiGeorge syndrome critical region gene 6	DGCR6	2.177568	0.060741	1.869998	0.10977	1.24	Down	0.04968	NM_005675	ILMN_1663685	Hs.474185	8214	22
DIP2 disco-interacting protein 2 homolog A	DIP2A	0.583998	0.025284	0.730824	0.03867	1.11	Up	0.01912	NM_206889	ILMN_1729272	Hs.189585	23181	21
DIS3 mitotic control homolog (S. cerevisiae)-like 2	DIS3L2	0.428916	0.04657	0.547211	0.00951	1.09	Up	0.04724	NM_152383	ILMN_1745737	Hs.471637	1E+05	2
DnaJ (Hsp40) homolog, subfamily C, member 19	DNAJC19	0.636456	0.047318	0.435492	0.04371	1.15	Down	0.02059	NM_145261	ILMN_1742109	Hs.230601	1E+05	3
Dopey family member 2	DOPEY2	0.541351	0.035423	0.302091	0.01888	1.18	Down	0.001	NM_005128	ILMN_1741711	Hs.204575	9980	21
Dual serine/threonine and tyrosine protein kinase	DSTYK	0.460002	0.094919	0.204371	0.02227	1.19	Down	0.03948	NM_015375	ILMN_3180989	Hs.6874	25778	1
Dual specificity phosphatase 2	DUSP2	0.279977	0.042027	0.101341	0.041	1.13	Down	0.02273	NM_004418	ILMN_1712959	Hs.1183	1844	2
Dual specificity phosphatase 23	DUSP23	2.902996	0.120635	2.43548	0.06614	1.38	Down	0.01453	NM_017823	ILMN_1659462	Hs.425801	54935	1
Dual-specificity tyrosine-(Y)-phosphorylation	DYRK4	1.601027	0.123497	1.072617	0.06046	1.44	Down	0.00853	NM_003845	ILMN_1681269	Hs.439530	8798	12
Dynein heavy chain domain 1	DNHD1	0.186005	0.039887	0.37878	0.05362	1.14	Up	0.02789	NM_144666	ILMN_1810267	Hs.377188	1E+05	11
Dynein, cytoplasmic 2, light intermediate chain 1	DYNC2LI1	0.519098	0.056061	0.271779	0.03789	1.19	Down	0.01064	NM_016008	ILMN_1811836	Hs.371597	51626	2
Dynein, light chain, LC8-type 1	DYNLL1	3.235636	0.084521	2.705829	0.12253	1.44	Down	0.01194	NM_001037494	ILMN_2300186	Hs.5120	8655	12
Dynein, light chain, LC8-type 1	DYNLL1	4.715415	0.088832	4.268858	0.13309	1.36	Down	0.03154	NM_003746	ILMN_1795227	Hs.5120	8655	12
endo-beta-N-acetylglucosaminidase	-	0.283246	0.0409	0.482189	0.06006	1.15	Up	0.03383	NM_022759	ILMN_1815366	-	64772	-
Enhancer of zeste homolog 2 (Drosophila)	EZH2	0.499515	0.031574	0.342365	0.02408	1.12	Down	0.00747	NM_152998	ILMN_1708105	Hs.444082	2146	7
Enhancer of zeste homolog 2 (Drosophila)	EZH2	0.15705	0.032187	0.266364	0.02028	1.08	Up	0.0283	NM_152998	ILMN_2364529	Hs.444082	2146	7
Enoyl Coenzyme A hydratase domain containing 1	ECHDC1	0.990703	0.074762	0.646735	0.05637	1.27	Down	0.01041	NM_018479	ILMN_2167011	Hs.486410	55862	6
EPH receptor A10	EPHA10	0.347016	0.032345	0.136689	0.01356	1.16	Down	0.00097	NM_173641	ILMN_1663263	Hs.129435	3E+05	1
Erythrocyte membrane protein band 4.1 like 4A	EPB41L4A	0.516538	0.051885	0.672627	0.02773	1.11	Up	0.03787	NM_022140	ILMN_1791867	Hs.584954	64097	5
Erythrocyte membrane protein band 4.1 like 4B	EPB41L4B	0.329651	0.023539	0.465992	0.01901	1.1	Up	0.00408	NM_019114	ILMN_1810051	Hs.591901	54566	9
Esterase D/formylglutathione hydrolase	ESD	3.759387	0.067202	3.422244	0.0536	1.26	Down	0.00778	NM_001984	ILMN_1720285	Hs.432491	2098	13
Eukaryotic translation elongation factor 1 beta 2	EEF1B2	5.343313	0.084316	5.055316	0.05564	1.22	Down	0.02914	NM_001037663	ILMN_2318725	Hs.421608	1933	2
Eukaryotic translation initiation factor 3, subunit C-	EIF3CL	1.150267	0.097823	0.833401	0.05635	1.25	Down	0.03089	NM_001099661	ILMN_3238570	Hs.535464	7E+05	16
Ewing sarcoma breakpoint region 1	EWSR1	3.278619	0.056729	2.962428	0.11543	1.25	Down	0.04923	NM_005243	ILMN_1727041	Hs.374477	2130	22
Exocyst complex component 3-like 2	EXOC3L2	0.334236	0.05177	0.559895	0.04754	1.17	Up	0.01835	NM_138568	ILMN_1670038	Hs.337557	90332	19
Family with sequence similarity 134, member C	FAM134C	0.683442	0.093421	0.423204	0.02749	1.2	Down	0.03691	NM_178126	ILMN_1666449	Hs.632262	2E+05	17
Family with sequence similarity 172, member A	FAM172A	2.295942	0.097896	1.968078	0.0776	1.26	Down	0.03935	NM_032042	ILMN_1654542	Hs.600086	83989	5
family with sequence similarity 39, member D pseudogene (FAM39DP) on chromosome 15.	-	3.404899	0.031812	3.609524	0.05423	1.15	Up	0.01736	NR_003659	ILMN_1713406	-	-	-
Family with sequence similarity 40, member B	FAM40B	1.832128	0.038513	1.976763	0.04245	1.11	Up	0.04508	NM_020704	ILMN_2161286	Hs.489988	57464	7
Family with sequence similarity 49, member B	FAM49B	1.159622	0.129646	0.780946	0.0152	1.3	Down	0.0273	NM_016623	ILMN_2122374	Hs.126941	51571	8
Far upstream element (FUSE) binding protein 1	FUBP1	0.359186	0.02109	0.492719	0.04899	1.1	Up	0.04631	NM_003902	ILMN_1776552	Hs.567380	8880	1
Fasciculation and elongation protein zeta 2 (zygin	FEZ2	4.988151	0.093152	4.646116	0.08749	1.27	Down	0.03671	NM_005102	ILMN_1739586	Hs.258563	9637	2

Fatty acid 2-hydroxylase	FA2H	0.758598	0.023397	0.968031	0.03851	1.16	Up	0.00351	NM_024306	ILMN_1791531	Hs.461329	79152	16
Fatty acid amide hydrolase 2	FAAH2	0.150527	0.033401	0.260757	0.02053	1.08	Up	0.0307	NM_174912	ILMN_1679158	Hs.496205	2E+05	X
Fatty acid binding protein 4, adipocyte	FABP4	2.821334	0.430339	1.569624	0.16147	2.38	Down	0.03449	NM_001442	ILMN_1773006	Hs.391561	2167	8
Fatty acid binding protein 5 (psoriasis-associated)	FABP5	3.489454	0.302276	2.552662	0.1381	1.91	Down	0.0304	NM_001444	ILMN_2146761	Hs.408061	2171	8
Fatty acid binding protein 5 (psoriasis-associated)	FABP5	1.092491	0.241711	0.430468	0.10036	1.58	Down	0.04471	NM_001444	ILMN_1696302	Hs.408061	2171	8
Fc fragment of IgE, high affinity I, receptor for;	FCER1G	4.247933	0.244386	3.438205	0.12005	1.75	Down	0.02483	NM_004106	ILMN_2123743	Hs.433300	2207	1
Fc fragment of IgG, high affinity Ia, receptor	FCGR1A	0.816963	0.154779	0.366625	0.06556	1.37	Down	0.03658	NM_000566	ILMN_2176063	Hs.77424	2209	1
Fc fragment of IgG, high affinity Ib, receptor	FCGR1B	1.470055	0.23507	0.805219	0.08721	1.59	Down	0.03795	NM_001017986	ILMN_2261600	Hs.534956	2210	1
Fc fragment of IgG, high affinity Ic, receptor (CD64)	FCGR1C	0.892443	0.07016	0.594742	0.04975	1.23	Down	0.01344	NM_001128589	ILMN_3247506	Hs.635062	1E+08	1
Ferredoxin-fold anticodon binding domain	FDXACB1	0.289461	0.052775	0.461064	0.01542	1.13	Up	0.02056	NM_138378	ILMN_3243529	Hs.697132	91893	11
Fidgetin	FIGN	0.562285	0.019694	0.68098	0.04168	1.09	Up	0.04206	NM_018086	ILMN_1676307	Hs.593650	55137	2
Formyl peptide receptor 1	FPR1	0.790899	0.102835	0.397086	0.01821	1.31	Down	0.00928	NM_002029	ILMN_2092118	Hs.753	2357	19
fragile site, folic acid type, rare, fra(10)(q23.3) or	-	0.142917	0.031576	0.277019	0.04066	1.1	Up	0.04039	NM_203441	ILMN_2375141	-	1E+05	-
FUS interacting protein (serine/arginine-rich) 1	FUSIP1	0.697344	0.009665	0.552881	0.02891	1.11	Down	0.0032	NM_054016	ILMN_2354649	Hs.3530	10772	1
FYVE, RhoGEF and PH domain containing 2	FGD2	1.286733	0.145709	0.696726	0.05073	1.51	Down	0.00872	NM_173558	ILMN_2115005	Hs.509664	2E+05	6
G protein-coupled bile acid receptor 1	GPBAR1	0.271865	0.036483	0.135258	0.03008	1.1	Down	0.02773	NM_170699	ILMN_1727709	Hs.160954	2E+05	2
G protein-coupled receptor 150	GPR150	0.20555	0.030238	0.323369	0.01283	1.09	Up	0.01155	NM_199243	ILMN_1708428	Hs.143315	3E+05	5
Galactose mutarotase (aldose 1-epimerase)	GALM	1.177501	0.223016	0.525321	0.04555	1.57	Down	0.02861	NM_138801	ILMN_1671482	Hs.435012	1E+05	2
Galactose-3-O-sulfotransferase 3	GAL3ST3	0.479277	0.023453	0.61745	0.02048	1.1	Up	0.00439	NM_033036	ILMN_1697081	Hs.208343	89792	11
Gamma-glutamyl cyclotransferase	GGCT	1.847989	0.044948	1.428737	0.059	1.34	Down	0.00132	NM_024051	ILMN_2101526	Hs.530024	79017	7
Gamma-glutamyl cyclotransferase	GGCT	1.263834	0.106355	0.908652	0.09005	1.28	Down	0.04357	NM_024051	ILMN_1745005	Hs.530024	79017	7
GAR1 ribonucleoprotein homolog (yeast)	GAR1	1.005853	0.024729	0.906724	0.01781	1.07	Down	0.0174	NM_032993	ILMN_1749752	Hs.69851	54433	4
GDP dissociation inhibitor 2	GDI2	3.919705	0.082527	3.678804	0.04889	1.18	Down	0.04581	NM_001494	ILMN_1754178	Hs.299055	2665	10
General transcription factor IIIA	GTF3A	3.132972	0.152871	2.682723	0.02818	1.37	Down	0.02746	NM_002097	ILMN_1658464	Hs.445977	2971	13
GLI pathogenesis-related 1	GLIPR1	2.073611	0.213463	1.441598	0.14204	1.55	Down	0.04879	NM_006851	ILMN_1769245	Hs.205558	11010	12
Glioblastoma amplified sequence	GBAS	1.46795	0.123454	1.041128	0.08517	1.34	Down	0.02934	NM_001483	ILMN_1778611	Hs.591069	2631	7
Glucagon-like peptide 1 receptor	GLP1R	0.457789	0.054188	0.695892	0.07128	1.18	Up	0.03756	NM_002062	ILMN_1787257	Hs.389103	2740	6
Glucose 6 phosphatase, catalytic, 3	G6PC3	2.340318	0.110119	2.000868	0.07417	1.27	Down	0.0431	NM_138387	ILMN_2127477	Hs.294005	92579	17
Glutamate receptor, metabotropic 4	GRM4	0.38814	0.037314	0.484167	0.01095	1.07	Up	0.0485	NM_000841	ILMN_1752843	Hs.654847	2914	6
Glutamate-ammonia ligase (glutamine synthetase)	GLUL	0.738431	0.009473	0.499903	0.08893	1.18	Down	0.03718	NM_001033056	ILMN_1653496	Hs.518525	2752	1
Glutathione S-transferase kappa 1	GSTK1	4.549522	0.112551	4.104431	0.06217	1.36	Down	0.01344	NM_015917	ILMN_1725241	Hs.390667	4E+05	7
Glycophorin C (Gerbich blood group)	GYPC	0.621707	0.112505	0.321777	0.03195	1.23	Down	0.04265	NM_016815	ILMN_1682332	Hs.59138	2995	2
Glycosyltransferase 25 domain containing 1	GLT25D1	2.851508	0.095907	2.438157	0.02041	1.33	Down	0.00559	NM_024656	ILMN_1727043	Hs.418795	79709	19
Glycosyltransferase 8 domain containing 1	GLT8D1	1.857207	0.07089	1.624847	0.04779	1.17	Down	0.03474	NM_018446	ILMN_1713290	Hs.297304	55830	3
Golgi associated, gamma adaptin ear containing,	GGA1	0.091195	0.039343	0.265917	0.05414	1.13	Up	0.04008	NM_013365	ILMN_1658175	Hs.499158	26088	22
GPN-loop GTPase 3	GPN3	2.151088	0.10445	1.75443	0.08971	1.32	Down	0.02803	NM_016301	ILMN_3239426	Hs.634680	51184	12
GTP cyclohydrolase I feedback regulator	GCHFR	3.046038	0.133954	2.638663	0.04583	1.33	Down	0.02815	NM_005258	ILMN_1694780	Hs.631717	2644	15
GTPase activating protein (SH3 domain) binding	G3BP2	2.333234	0.135039	1.860597	0.10202	1.39	Down	0.03147	NM_203504	ILMN_2381758	Hs.303676	9908	4
GTPase, very large interferon inducible 1	-	0.421901	0.050622	0.269813	0.03341	1.11	Down	0.04606	XM_495863	ILMN_1668526	-	4E+05	-
Guanosine monophosphate reductase	GMPR	0.559316	0.034436	0.384916	0.04182	1.13	Down	0.01816	NM_006877	ILMN_1729487	Hs.484741	2766	6
Guanylate binding protein 5	GBP5	0.717148	0.15588	0.211025	0.06154	1.42	Down	0.0234	NM_052942	ILMN_2114568	Hs.513726	1E+05	1
H3 histone, family 3A	H3F3A	6.149855	0.096182	5.879272	0.04266	1.21	Down	0.04224	NM_002107	ILMN_1656082	Hs.546259	3020	1
H3 histone, family 3A pseudogene	-	1.428729	0.101103	1.134146	0.04398	1.23	Down	0.03694	NR_002315	ILMN_2402936	-	4E+05	-
Haloacid dehalogenase-like hydrolase domain	HDHD2	1.737818	0.081352	1.471413	0.06648	1.2	Down	0.04433	NM_032124	ILMN_1702265	Hs.465041	84064	18
HAUS augmin-like complex, subunit 7	HAUS7	0.257926	0.036505	0.090561	0.03933	1.12	Down	0.02061	NM_207107	ILMN_1694890	Hs.170835	55559	X
hCG39912 (LOC642250), mRNA.	-	6.209085	0.054614	5.986441	0.04127	1.17	Down	0.01741	NM_001089592	ILMN_1663416	-	-	-
HEAT repeat containing 3	-	1.204022	0.036034	0.84429	0.10886	1.28	Down	0.02014	NM_017939	ILMN_1763663	-	55027	-
Heat shock protein family B (small), member 11	HSPB11	1.236829	0.067038	0.975778	0.0396	1.2	Down	0.01537	NM_016126	ILMN_1681340	Hs.525462	51668	1
Heat-responsive protein 12	HRSP12	0.653939	0.061857	0.417494	0.05232	1.18	Down	0.02668	NM_005836	ILMN_1807633	Hs.18426	10247	8
Hepatitis A virus cellular receptor 2	HAVCR2	4.259293	0.32137	3.38786	0.1464	1.83	Down	0.04861	NM_032782	ILMN_1693826	Hs.710500	84868	5
Hexokinase 3 (white cell)	HK3	1.010913	0.207251	0.43972	0.07123	1.49	Down	0.04032	NM_002115	ILMN_1670302	Hs.411695	3101	5
Histidine triad nucleotide binding protein 2	HINT2	3.192414	0.092838	2.906159	0.05305	1.22	Down	0.03667	NM_032593	ILMN_1697820	Hs.70573	84681	9
Histone cluster 1, H2ag	HIST1H2AG	0.167236	0.070432	0.355008	0.02868	1.14	Up	0.04851	NM_021064	ILMN_2184602	Hs.51011	8969	6
Homeobox A7	HOXA7	0.389573	0.050231	0.572402	0.04421	1.14	Up	0.03409	NM_006896	ILMN_1706478	Hs.660918	3204	7
HSPB (heat shock 27kDa) associated protein 1	HSPBAP1	2.605258	0.089404	2.961526	0.10007	1.28	Up	0.03777	NM_024610	ILMN_1797031	Hs.29169	79663	3
Hydroxyacid oxidase 2 (long chain)	HAO2	0.206667	0.047525	0.370039	0.04449	1.12	Up	0.04593	NM_016527	ILMN_1767474	Hs.659767	51179	1
Hydroxysteroid (17-beta) dehydrogenase 12	HSD17B12	2.820193	0.127466	2.176254	0.11886	1.56	Down	0.01015	NM_016142	ILMN_2094106	Hs.132513	51144	11
hypothetical gene supported by AK093158	-	0.935684	0.016952	1.074768	0.03428	1.1	Up	0.01087	NM_001013669	ILMN_1811227	-	4E+05	-
hypothetical gene supported by AK093729;	-	0.129289	0.04055	0.2844	0.01412	1.11	Up	0.0112	XM_499022	ILMN_1778111	-	4E+05	-
hypothetical gene supported by AK093729;	-	0.252866	0.043354	0.397109	0.01853	1.11	Up	0.02225	XM_499022	ILMN_1682912	-	4E+05	-
hypothetical LOC150051	-	0.746959	0.074332	0.961105	0.02014	1.16	Up	0.03197	XM_097792	ILMN_1696038	-	2E+05	-

hypothetical LOC255031 (FLJ35390), transcript variant 1, non-coding RNA.	-	3.849108	0.028728	4.054191	0.04203	1.15	Up	0.00689	NR_024416	ILMN_3269655	-	-	-
hypothetical LOC388282	-	0.21139	0.0335	0.30319	0.01639	1.07	Up	0.04902	XM_373690	ILMN_1741188	-	4E+05	-
hypothetical LOC440786	-	0.254508	0.033503	0.099037	0.02426	1.11	Down	0.00941	XM_496488	ILMN_1722913	-	4E+05	-
hypothetical LOC550643 (LOC550643), non-	-	4.793052	0.070498	4.536783	0.06583	1.19	Down	0.03768	NR_015367	ILMN_3247645	-	-	-
Hypothetical LOC643100	LOC643100	0.302542	0.035134	0.399276	0.01165	1.07	Up	0.03994	XM_931316	ILMN_1692045	Hs.136333	6E+05	4
Hypothetical LOC90768	MGC45800	0.247531	0.032257	0.422007	0.04431	1.13	Up	0.01899	AK094166	ILMN_1881514	Hs.175465	90768	4
hypothetical LOC91948	-	0.096738	0.027276	0.333496	0.07905	1.18	Up	0.02991	XM_378549	ILMN_1815218	-	91948	-
Hypothetical protein LOC100128440	LOC1001284	0.385922	0.073126	0.048442	0.07686	1.26	Down	0.01905	XM_001726725	ILMN_3191655	Hs.718548	1E+08	17
hypothetical protein LOC642947 (LOC642947),	-	6.114708	0.032906	6.243253	0.03038	1.09	Up	0.02842	NM_001039895	ILMN_2049343	-	-	-
Hypoxanthine phosphoribosyltransferase 1	HPRT1	1.656761	0.115317	1.259864	0.07027	1.32	Down	0.02598	NM_000194	ILMN_2056975	Hs.412707	3251	X
Hypoxanthine phosphoribosyltransferase 1	HPRT1	0.684324	0.109189	0.321073	0.07263	1.29	Down	0.03243	NM_000194	ILMN_1736940	Hs.412707	3251	X
IKAROS family zinc finger 3 (Aiolos)	IKZF3	0.326448	0.03554	0.17068	0.02726	1.11	Down	0.01318	NM_183228	ILMN_1669692	Hs.444388	22806	17
Immunoglobulin superfamily containing leucine-rich	ISLR	0.243286	0.051036	0.413893	0.03503	1.13	Up	0.03302	NM_201526	ILMN_1711009	Hs.710506	3671	15
Inducible T-cell co-stimulator	ICOS	0.122866	0.055812	0.313633	0.04077	1.14	Up	0.03285	NM_012092	ILMN_1669927	Hs.56247	29851	2
Inner centromere protein antigens 135/155kDa	INCENP	0.721909	0.02444	0.892087	0.045	1.13	Up	0.01594	NM_020238	ILMN_1698171	Hs.142179	3619	11
Interferon (alpha, beta and omega) receptor 2	IFNAR2	1.14137	0.076887	0.885454	0.02592	1.19	Down	0.01971	NM_207584	ILMN_1765146	Hs.708195	3455	21
Interferon stimulated exonuclease gene 20kDa	ISG20	2.183882	0.02472	2.5265	0.10126	1.27	Up	0.01668	NM_002201	ILMN_1659913	Hs.459265	3669	15
Interferon, alpha-inducible protein 27-like 2	IFI27L2	1.003458	0.053526	0.765964	0.05459	1.18	Down	0.02095	NM_032036	ILMN_3238560	Hs.94695	83982	14
Interleukin 16 (lymphocyte chemoattractant factor)	IL16	4.380029	0.106841	4.080629	0.03672	1.23	Down	0.03802	NM_172217	ILMN_1813572	Hs.459095	3603	15
Interleukin 17 receptor E-like	IL17REL	0.271933	0.038032	0.483622	0.01189	1.16	Up	0.00181	NM_001001694	ILMN_1722282	Hs.526712	4E+05	22
Interleukin 6 (interferon, beta 2)	IL6	0.055663	0.063744	0.515563	0.15174	1.38	Up	0.0314	NM_000600	ILMN_1699651	Hs.654458	3569	7
Kallikrein 1	KLK1	0.917414	0.047614	1.138467	0.02158	1.17	Up	0.00551	NM_002257	ILMN_1696450	Hs.123107	3816	19
Kallikrein-related peptidase 15	KLK15	0.189781	0.03703	0.397499	0.07546	1.15	Up	0.04838	NM_017509	ILMN_2347097	Hs.567535	55554	19
Karyopherin alpha 6 (importin alpha 7)	KPNA6	3.798768	0.075373	3.550169	0.04586	1.19	Down	0.03045	NM_012316	ILMN_1696021	Hs.470588	23633	1
Kelch domain containing 2	KLHDC2	2.905256	0.034718	2.734192	0.04703	1.13	Down	0.02641	NM_014315	ILMN_1741204	Hs.719215	23588	14
Kelch-like 5 (Drosophila)	KLHL5	0.478916	0.01954	0.582877	0.03038	1.07	Up	0.02814	NM_001007075	ILMN_2258234	Hs.272251	51088	4
Keratin 36	KRT36	0.140363	0.036535	0.258825	0.01129	1.09	Up	0.02117	NM_003771	ILMN_1790252	Hs.248189	8689	17
Keratin 73	KRT73	0.813594	0.038044	0.649176	0.05452	1.12	Down	0.04825	NM_175068	ILMN_1672120	Hs.55410	3E+05	12
Keratin 81	KRT81	0.203076	0.028152	0.435093	0.04257	1.17	Up	0.00391	NM_002282	ILMN_1677586	Hs.658118	3887	12
Keratin associated protein 4-5	KRTAP4-5	0.094068	0.042321	0.271373	0.03504	1.13	Up	0.01798	NM_033188	ILMN_1659475	Hs.514863	85289	17
KH domain containing, RNA binding, signal	KHDRBS1	4.654566	0.049974	4.416801	0.0544	1.18	Down	0.01817	NM_006559	ILMN_2076640	Hs.709204	10657	1
KIAA0090	KIAA0090	2.053516	0.103072	1.752089	0.05763	1.23	Down	0.04335	NM_015047	ILMN_2153280	Hs.439200	23065	1
KIAA1274	KIAA1274	0.455123	0.013266	0.196205	0.08049	1.2	Down	0.01922	NM_014431	ILMN_1796751	Hs.202351	27143	10
Killer cell immunoglobulin-like receptor, three domains, long cytoplasmic tail, 1	KIR3DL1	0.492019	0.084918	0.752167	0.03196	1.2	Up	0.02853	NM_013289	ILMN_2131828	Hs.645228	3811	19
Kinesin family member 22	KIF22	1.996208	0.06032	1.803483	0.04949	1.14	Down	0.04845	NM_007317	ILMN_3234884	Hs.613351	3835	16
Lactate dehydrogenase B	LDHB	3.472777	0.145675	2.892126	0.1632	1.5	Down	0.03781	NM_002300	ILMN_1728132	Hs.446149	3945	12
Leber congenital amaurosis 5-like	LCA5L	0.307269	0.045224	0.446849	0.02813	1.1	Up	0.03954	NM_152505	ILMN_1665657	Hs.517284	2E+05	21
Lectin, galactoside-binding, soluble, 1	LGALS1	4.38019	0.281726	3.44413	0.10836	1.91	Down	0.02109	NM_002305	ILMN_1723978	Hs.445351	3956	22
Lectin, galactoside-binding, soluble, 3 binding	LGALS3BP	2.223252	0.064573	2.628997	0.09097	1.32	Up	0.01087	NM_005567	ILMN_1659688	Hs.514535	3959	17
Lectin, galactoside-binding, soluble, 9	LGALS9	0.434897	0.113851	0.070836	0.04247	1.29	Down	0.02413	NM_009587	ILMN_1715760	Hs.81337	3965	17
Leucine rich repeat containing 16B	LRRC16B	0.543283	0.025088	0.381193	0.02745	1.12	Down	0.00478	NM_138360	ILMN_1777708	Hs.26135	90668	14
Leucine rich repeat containing 16B	LRRC16B	0.660185	0.010244	0.550073	0.0273	1.08	Down	0.00923	NM_138360	ILMN_3244860	Hs.26135	90668	14
Leucine-rich repeat-containing G protein-coupled	LGR5	0.438422	0.022757	0.604218	0.05121	1.12	Up	0.02532	NM_003667	ILMN_1702310	Hs.658889	8549	12
Leucyl/cystinyl aminopeptidase	LNPEP	0.426157	0.021854	0.55606	0.01559	1.09	Up	0.00288	NM_005575	ILMN_1798433	Hs.656905	4012	5
Leukocyte immunoglobulin-like receptor, subfamily A (with TM domain), member 5	LILRA5	0.550631	0.106406	0.247248	0.05298	1.23	Down	0.04336	NM_021250	ILMN_2266595	Hs.710986	4E+05	19
Leukocyte immunoglobulin-like receptor, subfamily B (with TM and ITIM domains), member 3	LILRB3	6.362737	0.046024	6.572775	0.07115	1.16	Up	0.04788	NM_006864	ILMN_2406132	Hs.631592	11025	19
Leukocyte specific transcript 1	LST1	0.725374	0.130506	0.27321	0.06052	1.37	Down	0.01999	NM_205840	ILMN_2345353	Hs.436066	7940	6
Leukocyte specific transcript 1	LST1	0.751621	0.075128	0.418266	0.09629	1.26	Down	0.03421	NM_205839	ILMN_1688373	Hs.436066	7940	6
Ligase I, DNA, ATP-dependent	LIG1	0.52131	0.021829	0.395408	0.04644	1.09	Down	0.04955	NM_000234	ILMN_1706779	Hs.1770	3978	19
Low density lipoprotein receptor-related protein 5-	LRP5L	0.276475	0.02076	0.162834	0.01796	1.08	Down	0.00608	NM_182492	ILMN_1718633	Hs.634058	91355	22
LSM3 homolog, U6 small nuclear RNA associated	LSM3	4.28173	0.107404	3.9502	0.05149	1.26	Down	0.03185	NM_014463	ILMN_1719032	Hs.111632	27258	3
LSM5 homolog, U6 small nuclear RNA associated	LSM5	3.055189	0.149916	2.521611	0.10097	1.45	Down	0.02555	NM_012322	ILMN_1737947	Hs.424908	23658	7
Lymphoblastic leukemia derived sequence 1	LYL1	1.160038	0.174619	0.689683	0.05296	1.39	Down	0.0419	NM_005583	ILMN_2216582	Hs.46446	4066	19
Lymphocyte-specific protein tyrosine kinase	LCK	0.197604	0.028718	0.34291	0.04946	1.11	Up	0.04405	NM_005356	ILMN_1699991	Hs.470627	3932	1
Lysine-rich coiled-coil 1	KRCC1	0.949467	0.052602	0.685088	0.07114	1.2	Down	0.02438	NM_016618	ILMN_2091375	Hs.469254	51315	2
Major histocompatibility complex, class II, DP beta	HLA-DPB1	0.540253	0.130351	0.183526	0.02986	1.28	Down	0.03715	NM_002121	ILMN_1749070	Hs.485130	3115	6
Major histocompatibility complex, class II, DQ	HLA-DQA2	0.218262	0.0459	0.437188	0.02538	1.16	Up	0.00585	NM_020056	ILMN_1680144	Hs.591798	3118	6
major histocompatibility complex, class II, DQ beta	-	0.500186	0.011187	0.616085	0.03997	1.08	Up	0.03147	NM_182549	ILMN_1741648	-	3120	-

Malectin	MLEC	2.847092	0.074796	2.58287	0.06963	1.2	Down	0.04145	NM_014730	ILMN_1657495	Hs.714322	9761	12
MAM domain containing 4	MAMDC4	0.259988	0.025745	0.343746	0.01967	1.06	Up	0.04147	NM_206920	ILMN_1740759	Hs.376780	2E+05	9
MAM domain containing	MDGA1	0.819345	0.058036	1.033075	0.03269	1.16	Up	0.0184	NM_153487	ILMN_1720595	Hs.437993	3E+05	6
Mannan-binding lectin serine peptidase 1 (C4/C2 activating component of Ra-reactive factor)	MASP1	0.274506	0.040624	0.529281	0.06219	1.19	Up	0.01397	NM_001031849	ILMN_1801996	Hs.89983	5648	3
mannose-binding lectin (protein A) 1, pseudogene 1 (MBL1P1) on chromosome 10.	-	0.874021	0.069548	1.116435	0.06374	1.18	Up	0.04236	NR_002724	ILMN_1745141	-	-	-
Matrix metallopeptidase 19	MMP19	0.341903	0.022913	0.21799	0.03714	1.09	Down	0.02959	NM_002429	ILMN_1711062	Hs.591033	4327	12
mature T-cell proliferation 1	-	1.575913	0.062007	1.084798	0.06341	1.41	Down	0.00146	NM_014221	ILMN_1814230	-	4515	-
Mdm2 p53 binding protein homolog (mouse)	MDM2	0.645741	0.037857	0.846768	0.07261	1.15	Up	0.04945	NM_002392	ILMN_1736829	Hs.484551	4193	12
Melanoregulin	MREG	0.748005	0.024496	0.556262	0.02048	1.14	Down	0.00096	NM_018000	ILMN_1713679	Hs.707104	55686	2
Membrane bound O-acyltransferase domain	MBOAT1	0.651607	0.056003	0.372181	0.03364	1.21	Down	0.00522	NM_001080480	ILMN_1764082	Hs.377830	2E+05	6
Mesoderm development candidate 2	MESDC2	0.265059	0.009894	0.04087	0.08059	1.17	Down	0.03281	NM_015154	ILMN_1719797	Hs.578450	23184	15
microRNA 346 (MIR346), microRNA.	-	0.19599	0.017308	0.303111	0.02342	1.08	Up	0.01035	NR_029907	ILMN_3308748	-	-	-
microRNA 591 (MIR591), microRNA.	-	0.212455	0.036258	0.345108	0.03893	1.1	Up	0.04695	NR_030322	ILMN_3309069	-	-	-
Microsomal glutathione S-transferase 3	MGST3	5.48221	0.052534	5.145601	0.1159	1.26	Down	0.03827	NM_004528	ILMN_1751956	Hs.191734	4259	1
Microtubule-associated protein, RP/EB family,	MAPRE2	1.589535	0.047098	1.386556	0.01857	1.15	Down	0.00704	NM_014268	ILMN_1695276	Hs.532824	10982	18
Mindbomb homolog 2 (Drosophila)	MIB2	0.745048	0.046258	1.010078	0.0971	1.2	Up	0.04885	NM_080875	ILMN_2282077	Hs.135805	1E+05	1
Minichromosome maintenance complex	MCM8	5.42913	0.058879	5.63526	0.04938	1.15	Up	0.03642	NM_032485	ILMN_1798581	Hs.631506	84515	20
Mitochondrial ribosomal protein L23	MRPL23	2.520372	0.100208	2.212097	0.07388	1.24	Down	0.04805	NM_021134	ILMN_1806123	Hs.3254	6150	11
Mitochondrial ribosomal protein S36	MRPS36	1.010581	0.064737	0.736467	0.07545	1.21	Down	0.03297	NM_033281	ILMN_1807095	Hs.631971	92259	5
Mitochondrial ribosomal protein S5	MRPS5	1.719867	0.051424	1.514679	0.04074	1.15	Down	0.02039	NM_031902	ILMN_1760441	Hs.655259	64969	2
MORC family CW-type zinc finger 1	MORC1	0.206092	0.033562	0.408691	0.06786	1.15	Up	0.03673	NM_014429	ILMN_1808970	Hs.591296	27136	3
Mortality factor 4 like 1	MORF4L1	4.773257	0.061147	4.517395	0.0654	1.19	Down	0.02889	NM_206839	ILMN_1760676	Hs.374503	10933	15
MU-2/AP1M2 domain containing, death-inducing	MUDENG	1.218268	0.103639	0.85852	0.05492	1.28	Down	0.02202	NM_018229	ILMN_2204297	Hs.597349	55745	14
Multiple C2 domains, transmembrane 2	MCTP2	0.117033	0.046472	0.259914	0.0249	1.1	Up	0.0351	NM_018349	ILMN_1792682	Hs.592017	55784	15
Musashi homolog 1 (Drosophila)	MSI1	0.392229	0.057225	0.602997	0.0328	1.16	Up	0.01871	NM_002442	ILMN_1739601	Hs.158311	4440	12
MYC induced nuclear antigen	MINA	1.36763	0.074258	1.149342	0.0476	1.16	Down	0.04815	NM_032778	ILMN_1689624	Hs.607776	84864	3
Myeloid cell nuclear differentiation antigen	MNDA	0.858132	0.133375	0.391296	0.06148	1.38	Down	0.01911	NM_002432	ILMN_1738992	Hs.153837	4332	1
Myosin, light chain 12B, regulatory	MYL12B	6.48683	0.028377	6.35599	0.02117	1.09	Down	0.01014	NM_033546	ILMN_1654016	Hs.464472	1E+05	18
Myosin, light chain 5, regulatory	MYL5	0.259484	0.040406	0.082348	0.03787	1.13	Down	0.01864	NM_002477	ILMN_2203588	Hs.410970	4636	4
N-acetylneuraminate pyruvate lyase	NPL	2.342267	0.259152	1.530622	0.05527	1.76	Down	0.02214	NM_030769	ILMN_1782070	Hs.496969	80896	1
N-acetylneuraminate pyruvate lyase	NPL	2.098429	0.252444	1.341257	0.07588	1.69	Down	0.02834	NM_030769	ILMN_2149494	Hs.496969	80896	1
NADH dehydrogenase (ubiquinone) 1 alpha	NDUFA1	5.66233	0.047519	5.354655	0.08887	1.24	Down	0.02242	NM_004541	ILMN_1784286	Hs.534168	4694	X
NADH dehydrogenase (ubiquinone) 1 alpha subcomplex, assembly factor 3	NDUFAF3	4.408865	0.076271	4.104225	0.04531	1.24	Down	0.0139	NM_199069	ILMN_2354515	Hs.31387	25915	3
NADH dehydrogenase (ubiquinone) 1 beta	NDUFB2	4.999379	0.160888	4.584396	0.05256	1.33	Down	0.04967	NM_004546	ILMN_2117330	Hs.655788	4708	7
NADH dehydrogenase (ubiquinone) 1 beta	NDUFB3	4.678893	0.106848	4.312159	0.09184	1.29	Down	0.0405	NM_002491	ILMN_2119945	Hs.109760	4709	2
NADH dehydrogenase (ubiquinone) 1 beta	NDUFB9	3.276655	0.079467	2.990918	0.05954	1.22	Down	0.02815	NM_005005	ILMN_3243859	Hs.15977	4715	8
NADH dehydrogenase (ubiquinone) flavoprotein 2,	NDUFV2	4.612431	0.117438	4.205897	0.03596	1.33	Down	0.0162	NM_021074	ILMN_2086417	Hs.464572	4729	18
natriuretic peptide receptor B/guanylate cyclase B (atrionatriuretic peptide receptor B)	-	0.47761	0.042573	0.656307	0.05362	1.13	Up	0.04013	NM_000907	ILMN_1681994	-	4882	-
NCK-associated protein 1-like	NCKAP1L	1.950642	0.190475	1.345025	0.1468	1.52	Down	0.04539	NM_005337	ILMN_1674250	Hs.182014	3071	12
NDRG family member 3	NDRG3	0.563407	0.036192	0.325598	0.03302	1.18	Down	0.00284	NM_022477	ILMN_2385097	Hs.437338	57446	20
Negative regulator of ubiquitin-like proteins 1	NUB1	0.293521	0.053194	0.461055	0.02241	1.12	Up	0.02725	NM_016118	ILMN_2150661	Hs.647082	51667	7
Nescient helix loop helix 1	NHLH1	0.61522	0.024401	0.809328	0.05997	1.14	Up	0.02407	NM_005598	ILMN_1805457	Hs.30956	4807	1
Neurofibromin 2 (merlin)	NF2	0.220884	0.026396	0.356816	0.02245	1.1	Up	0.00777	NM_181825	ILMN_1747580	Hs.187898	4771	22
Neuron derived neurotrophic factor	NENF	3.77686	0.013384	3.558016	0.07244	1.16	Down	0.02493	NM_013349	ILMN_2142554	Hs.461787	29937	1
Neuropeptides B/W receptor 2	NPBWR2	0.288351	0.064616	0.491953	0.04825	1.15	Up	0.045	NM_005286	ILMN_1769358	Hs.248118	2832	20
Neuropilin (NRP) and tolloid (TLL)-like 1	NETO1	0.620744	0.023667	0.740847	0.00712	1.09	Up	0.00282	NM_138966	ILMN_1684081	Hs.465407	81832	18
Niemann-Pick disease, type C2	NPC2	6.145792	0.172717	5.608492	0.12156	1.45	Down	0.04385	NM_006432	ILMN_1716678	Hs.433222	10577	14
Non-metastatic cells 1, protein (NM23A) expressed	NME1	3.047816	0.134668	2.271769	0.26064	1.71	Down	0.03827	NM_000269	ILMN_1741133	Hs.463456	4830	17
Non-metastatic cells 1, protein (NM23A) expressed	NME1	3.971808	0.154553	3.511862	0.05047	1.38	Down	0.03	NM_001018136	ILMN_1662905	Hs.463456	4830	17
NPC-A-7	LOC1001296	1.09974	0.03449	0.699827	0.03287	1.32	Down	0.00016	XM_001726834	ILMN_3259146	Hs.718648	1E+08	19
Nuclear factor of kappa light polypeptide gene enhancer in B-cells inhibitor, zeta	NFKBIZ	2.392196	0.129196	2.873504	0.14004	1.4	Up	0.04491	NM_001005474	ILMN_1719695	Hs.319171	64332	3
Nuclear factor of kappa light polypeptide gene enhancer in B-cells inhibitor, zeta	NFKBIZ	0.903474	0.035732	1.070421	0.04465	1.12	Up	0.02666	NM_031419	ILMN_1770260	Hs.319171	64332	3
Nuclear receptor binding protein 2	NRBP2	0.252904	0.057139	0.001472	0.06249	1.19	Down	0.02497	NM_178564	ILMN_1733248	Hs.521926	3E+05	8
Nuclear receptor subfamily 4, group A, member 1	NR4A1	0.549187	0.021981	0.441811	0.01148	1.08	Down	0.00493	NM_002135	ILMN_1661178	Hs.524430	3164	12
Nucleolar and spindle associated protein 1	NUSAP1	0.243756	0.015482	0.069571	0.03862	1.13	Down	0.00578	NM_018454	ILMN_1726720	Hs.615092	51203	15
NudC domain containing 2	NUDCD2	1.296061	0.142776	0.811579	0.06858	1.4	Down	0.02226	NM_145266	ILMN_1751589	Hs.140443	1E+05	5

NudC domain containing 2	NUDCD2	0.521429	0.076702	0.292738	0.01971	1.17	Down	0.02777	NM_145266	ILMN_2048822	Hs.140443	1E+05	5
Nudix (nucleoside diphosphate linked moiety X)-	NUDT1	0.906373	0.080512	0.610189	0.08236	1.23	Down	0.04224	NM_198948	ILMN_2330243	Hs.534331	4521	7
Olfactory receptor, family 10, subfamily G, member	OR10G3	0.29096	0.018958	0.401493	0.02963	1.08	Up	0.02001	NM_001005465	ILMN_1726523	Hs.554581	26533	14
Olfactory receptor, family 11, subfamily H, member	OR11H1	0.222412	0.060555	0.407094	0.04242	1.14	Up	0.04665	NM_001005239	ILMN_2072401	Hs.554706	81061	22
Olfactory receptor, family 2, subfamily D, member	OR2D3	0.573171	0.036583	0.756963	0.02329	1.14	Up	0.00545	NM_001004684	ILMN_1707351	Hs.446766	1E+05	11
Olfactory receptor, family 4, subfamily L, member 1	OR4L1	0.180726	0.027712	0.415375	0.02465	1.18	Up	0.00073	NM_001004717	ILMN_1722496	Hs.553574	1E+05	14
Olfactory receptor, family 6, subfamily Q, member	OR6Q1	0.18999	0.009566	0.331364	0.0101	1.1	Up	5.28E-05	NM_001005186	ILMN_1686245	Hs.690432	2E+05	11
Oligosaccharyltransferase complex subunit	OSTC	2.895626	0.157622	2.444988	0.08541	1.37	Down	0.04568	NM_021227	ILMN_2056167	Hs.445803	58505	4
Oligosaccharyltransferase complex subunit	OSTC	4.705633	0.041729	4.387072	0.09419	1.25	Down	0.02133	NM_021227	ILMN_3236156	Hs.445803	58505	4
ORM1-like 1 (S. cerevisiae)	ORMDL1	3.664135	0.047059	3.319803	0.06896	1.27	Down	0.00619	NM_016467	ILMN_1698406	Hs.709387	94101	2
Outer dense fiber of sperm tails 4	ODF4	0.823093	0.025686	0.9897	0.04251	1.12	Up	0.01534	NM_153007	ILMN_1783656	Hs.186045	1E+05	17
Oxidoreductase NAD-binding domain containing 1	OXNAD1	0.289892	0.046674	0.136487	0.00423	1.11	Down	0.01696	NM_138381	ILMN_1764770	Hs.655449	92106	3
Paf1, RNA polymerase II associated factor,	PAF1	2.489682	0.094152	2.853528	0.11382	1.29	Up	0.0489	NM_019088	ILMN_1669508	Hs.466714	54623	19
Parvalbumin	PVALB	0.293151	0.042798	0.134836	0.03535	1.12	Down	0.0291	NM_002854	ILMN_2069224	Hs.295449	5816	22
PCTAIRE protein kinase 3	PCTK3	0.392697	0.032774	0.250493	0.0284	1.1	Down	0.01684	NM_212503	ILMN_1784110	Hs.445402	5129	1
PDZ and LIM domain 5	PDLIM5	1.264969	0.013166	1.155793	0.04178	1.08	Down	0.04703	NM_006457	ILMN_1692864	Hs.480311	10611	4
Peptidylprolyl isomerase A (cyclophilin A)-like 4A	PPIAL4A	5.832671	0.111072	5.428017	0.08981	1.32	Down	0.02984	NM_178230	ILMN_3236259	Hs.573713	2E+05	1
peptidylprolyl isomerase A processed pseudogene	-	4.423218	0.119676	3.847899	0.12411	1.49	Down	0.01567	NR_000030	ILMN_2205211	-	1E+05	-
Peroxiredoxin 3	PRDX3	3.885337	0.117367	3.497329	0.08572	1.31	Down	0.03704	NM_006793	ILMN_2395969	Hs.523302	10935	10
Phosphate cytidylyltransferase 2, ethanolamine	PCYT2	0.929361	0.073635	0.661515	0.06531	1.2	Down	0.03458	NM_002861	ILMN_1652846	Hs.645248	5833	17
Phosphatidylinositol glycan anchor biosynthesis,	PIGF	2.173347	0.02133	1.840706	0.10976	1.26	Down	0.02479	NM_002643	ILMN_1808938	Hs.468415	5281	2
Phosphofurin acidic cluster sorting protein 1	PACS1	0.642249	0.056938	0.467974	0.03796	1.13	Down	0.04368	NM_018026	ILMN_1716488	Hs.644326	55690	11
Phosphofurin acidic cluster sorting protein 2	PACS2	0.476485	0.029169	0.626674	0.02827	1.11	Up	0.01012	NM_015197	ILMN_1719864	Hs.525626	23241	14
Phosphoglycerate mutase family member 4	PGAM4	2.744969	0.055186	2.460899	0.0661	1.22	Down	0.01643	NM_001029891	ILMN_1691104	Hs.632822	4E+05	X
Phospholipase A2, group III	PLA2G3	0.399754	0.051753	0.593247	0.02673	1.14	Up	0.01597	NM_015715	ILMN_1746769	Hs.149623	50487	22
Phospholipase A2, group XV	PLA2G15	1.543174	0.059408	1.243725	0.06572	1.23	Down	0.01485	NM_012320	ILMN_1756910	Hs.632199	23659	16
Phospholipase B domain containing 1	PLBD1	4.139023	0.119432	3.679586	0.09172	1.38	Down	0.02249	NM_024829	ILMN_1707286	Hs.131933	79887	12
Phosphoserine phosphatase	PSPH	0.486925	0.015186	0.388869	0.03568	1.07	Down	0.04477	NM_004577	ILMN_1776105	Hs.512656	5723	7
Pituitary tumor-transforming 1	PTTG1	0.722181	0.074889	0.400161	0.09178	1.25	Down	0.03471	NM_004219	ILMN_2042771	Hs.350966	9232	5
Polybromo 1	PBRM1	0.263146	0.027516	0.073167	0.04948	1.14	Down	0.01531	NM_181042	ILMN_1723822	Hs.189920	55193	3
Polymerase (DNA directed), delta 2, regulatory	POLD2	1.235203	0.057301	1.007895	0.04801	1.17	Down	0.02278	NM_001127218	ILMN_3305304	Hs.306791	5425	7
Polymerase (RNA) I polypeptide E, 53kDa	POLR1E	1.196364	0.049655	0.987967	0.05404	1.16	Down	0.02958	NM_022490	ILMN_2110167	Hs.591087	64425	9
Polymerase (RNA) III (DNA directed) polypeptide	POLR3GL	1.361278	0.156265	0.903197	0.08618	1.37	Down	0.04251	NM_032305	ILMN_1760667	Hs.591456	84265	1
Polyribonucleotide nucleotidyltransferase 1	PNPT1	5.54293	0.078465	5.814125	0.0525	1.21	Up	0.02833	NM_033109	ILMN_2051408	Hs.388733	87178	2
Potassium channel modulatory factor 1	KCMF1	0.25854	0.03283	0.05518	0.04167	1.15	Down	0.00862	NM_020122	ILMN_1839052	Hs.654968	56888	2
Potassium channel, subfamily K, member 3	KCNK3	0.276806	0.038338	0.121247	0.04171	1.11	Down	0.03347	NM_002246	ILMN_1768483	Hs.645288	3777	2
Potassium voltage-gated channel, Shaw-related	KCNC4	0.385437	0.039884	0.241081	0.02393	1.11	Down	0.02102	NM_004978	ILMN_1792382	Hs.153521	3749	1
POTE ankyrin domain family, member K,	-	3.769528	0.112499	3.321365	0.14241	1.36	Down	0.0485	NM_001017421	ILMN_1814998	-	4E+05	-
PQ loop repeat containing 3	PQLC3	2.286107	0.172353	1.685991	0.16226	1.52	Down	0.04437	NM_152391	ILMN_1814213	Hs.274415	1E+05	2
Praja ring finger 1	PJA1	0.679156	0.045782	0.818378	0.027	1.1	Up	0.03962	NM_145119	ILMN_1734810	Hs.522679	64219	X
PREDICTED: alveolar soft part sarcoma chromosome region, candidate 1 (ASPSCR1),	-	0.891722	0.068988	0.580156	0.05985	1.24	Down	0.01429	XM_001132706	ILMN_1688180	-	-	-
PREDICTED: ATPase, H+/K+ exchanging, beta polypeptide (ATP4B), mRNA.	-	0.503883	0.035004	0.66892	0.05484	1.12	Up	0.04428	XM_939372	ILMN_1702257	-	-	-
PREDICTED: Beta-defensin 108B-like	-	0.536807	0.017724	0.698453	0.05898	1.12	Up	0.03933	XM_001720802	ILMN_3237529	-	-	-
PREDICTED: chromosome 16 open reading frame 50, transcript variant 3 (C16orf50), mRNA.	-	0.100151	0.032369	0.253632	0.03646	1.11	Up	0.01986	XM_945357	ILMN_1689710	-	-	-
PREDICTED: fatty acid binding protein 5-like 2	-	4.616779	0.284759	3.527001	0.14554	2.13	Down	0.01436	XM_001134012	ILMN_3266606	-	-	-
PREDICTED: fatty acid binding protein 5-like 2	-	2.286229	0.32478	1.376852	0.14078	1.88	Down	0.04239	XM_001721172	ILMN_3178258	-	-	-
PREDICTED: fibrinogen-like 1, transcript variant 5	-	0.739687	0.046179	0.935606	0.03892	1.15	Up	0.01759	XM_937886	ILMN_1803597	-	-	-
PREDICTED: germ and embryonic stem cell enriched protein STELLA (STELLAR), misc RNA.	-	0.231381	0.029128	0.388862	0.03276	1.12	Up	0.01147	XR_017914	ILMN_1683474	-	-	-
PREDICTED: heat shock 90kDa protein 1, alpha-like 3, transcript variant 3 (HSPCAL3), mRNA.	-	0.588858	0.037417	0.371033	0.05267	1.16	Down	0.01501	XM_934529	ILMN_1700810	-	-	-
PREDICTED: hypothetical LOC100131271	-	0.096322	0.068003	0.267486	0.01462	1.13	Up	0.04907	XM_001723153	ILMN_3199628	-	-	-
PREDICTED: hypothetical LOC387686	-	0.262461	0.039391	0.394124	0.01401	1.1	Up	0.01983	XM_373463	ILMN_1762189	-	4E+05	-
PREDICTED: hypothetical LOC642956	-	2.7056	0.262843	1.817496	0.09899	1.85	Down	0.01951	XM_938166	ILMN_3210741	-	-	-
PREDICTED: hypothetical LOC646823	-	0.709369	0.040073	0.546912	0.01953	1.12	Down	0.01078	XM_933959	ILMN_1753600	-	-	-
PREDICTED: hypothetical LOC650683	-	1.043872	0.026693	1.191559	0.01157	1.11	Up	0.00227	XM_939770	ILMN_1677363	-	-	-
PREDICTED: hypothetical protein LOC100129034 (LOC100129034), mRNA.	-	0.828193	0.096031	0.581375	0.02314	1.19	Down	0.04661	XM_001720357	ILMN_3256478	-	-	-

PREDICTED: hypothetical protein LOC100129445 (LOC100129445), mRNA.	-	0.08865	0.031758	0.246458	0.02056	1.12	Up	0.00587	XM_001720255	ILMN_3240773	-	-	-
PREDICTED: hypothetical protein LOC100130598 (LOC100130598), mRNA.	-	1.308507	0.026992	1.480523	0.0224	1.13	Up	0.0027	XM_001721180	ILMN_3251944	-	-	-
PREDICTED: hypothetical protein LOC100131835	-	0.529492	0.075277	0.760125	0.042	1.17	Up	0.03676	XM_001713758	ILMN_3243568	-	-	-
PREDICTED: hypothetical protein LOC100132767 (LOC100132767), mRNA.	-	0.250416	0.010153	0.345737	0.00879	1.07	Up	0.00039	XM_001725561	ILMN_3293159	-	-	-
PREDICTED: hypothetical protein LOC100133876, transcript variant 2 (LOC100133876), mRNA.	-	7.108349	0.040199	7.318047	0.04427	1.16	Up	0.01272	XM_001713723	ILMN_3202483	-	-	-
PREDICTED: hypothetical protein LOC100134598 (LOC100134598), mRNA.	-	0.413812	0.075116	0.681314	0.06493	1.2	Up	0.03586	XM_001724239	ILMN_3239014	-	-	-
PREDICTED: hypothetical protein LOC340094	-	0.206328	0.024877	0.300662	0.01951	1.07	Up	0.02451	XM_944590	ILMN_1722916	-	-	-
PREDICTED: hypothetical protein LOC641996	-	0.591061	0.035677	0.769694	0.06001	1.13	Up	0.04298	XM_936043	ILMN_1773390	-	-	-
PREDICTED: hypothetical protein LOC642031	-	1.385205	0.034293	1.533279	0.04588	1.11	Up	0.04148	XM_936101	ILMN_1655694	-	-	-
PREDICTED: hypothetical protein LOC642621	-	0.359765	0.029363	0.474745	0.02918	1.08	Up	0.03209	XM_926093	ILMN_1805747	-	-	-
PREDICTED: hypothetical protein LOC643402	-	0.965925	0.034397	1.259788	0.03967	1.23	Up	0.00139	XM_926737	ILMN_1705749	-	-	-
PREDICTED: hypothetical protein LOC644898	-	0.329013	0.019118	0.472751	0.01615	1.1	Up	0.00121	XM_932506	ILMN_1674853	-	-	-
PREDICTED: hypothetical protein LOC648138	-	0.251081	0.022231	0.358606	0.03476	1.08	Up	0.04033	XM_943208	ILMN_1665733	-	-	-
PREDICTED: hypothetical protein LOC649823	-	0.714939	0.034296	0.931847	0.05564	1.16	Up	0.01604	XM_943936	ILMN_1680936	-	-	-
PREDICTED: hypothetical protein LOC649956	-	0.5306	0.028594	0.702327	0.01221	1.13	Up	0.00148	XM_939037	ILMN_1725407	-	-	-
PREDICTED: hypothetical protein LOC651287	-	0.217232	0.023648	0.378883	0.03133	1.12	Up	0.00623	XM_940415	ILMN_1713128	-	-	-
PREDICTED: hypothetical protein LOC652529	-	0.354973	0.004801	0.279404	0.01545	1.05	Down	0.00343	XM_942013	ILMN_1755526	-	-	-
PREDICTED: KIAA1641, transcript variant 4	-	0.268308	0.027162	0.376256	0.02246	1.08	Up	0.02215	XM_944049	ILMN_1754975	-	-	-
PREDICTED: misc_RNA (LOC100128410),	-	4.704391	0.079885	4.436531	0.04209	1.2	Down	0.02507	XR_037117	ILMN_3268914	-	-	-
PREDICTED: misc_RNA (LOC100128460),	-	1.287432	0.052296	1.515318	0.05719	1.17	Up	0.02593	XR_037866	ILMN_3187283	-	-	-
PREDICTED: misc_RNA (LOC100129424),	-	5.688514	0.146662	5.264919	0.07099	1.34	Down	0.04068	XR_037795	ILMN_3194217	-	-	-
PREDICTED: misc_RNA (LOC100130746),	-	0.278824	0.059155	0.087852	0.02976	1.14	Down	0.02791	XR_037442	ILMN_3256540	-	-	-
PREDICTED: misc_RNA (LOC100131940),	-	2.361129	0.077069	2.080354	0.07746	1.21	Down	0.04236	XR_037582	ILMN_3275696	-	-	-
PREDICTED: misc_RNA (LOC100132199),	-	0.495883	0.081086	0.237283	0.03077	1.2	Down	0.02458	XR_039723	ILMN_3289730	-	-	-
PREDICTED: misc_RNA (LOC100132547),	-	1.057151	0.070708	0.792198	0.07123	1.2	Down	0.03854	XR_039468	ILMN_3236130	-	-	-
PREDICTED: misc_RNA (LOC100132795),	-	4.350428	0.103179	3.932911	0.03482	1.34	Down	0.00862	XR_037619	ILMN_3280019	-	-	-
PREDICTED: misc_RNA (LOC100132795),	-	3.782873	0.050986	3.567759	0.06524	1.16	Down	0.04077	XR_037785	ILMN_3201986	-	-	-
PREDICTED: misc_RNA (LOC100132863),	-	2.177813	0.096339	1.891073	0.06343	1.22	Down	0.04742	XR_036905	ILMN_3205271	-	-	-
PREDICTED: misc_RNA (LOC100133273),	-	6.113483	0.064467	5.774731	0.0328	1.26	Down	0.00338	XR_039238	ILMN_3292678	-	-	-
PREDICTED: misc_RNA (LOC100134609),	-	0.106999	0.036802	0.257225	0.04741	1.11	Up	0.04634	XR_039698	ILMN_3243797	-	-	-
PREDICTED: misc_RNA (LOC338870), miscRNA.	-	4.75852	0.083538	4.361408	0.09397	1.32	Down	0.01961	XR_038789	ILMN_3294074	-	-	-
PREDICTED: misc_RNA (LOC341965), miscRNA.	-	1.273441	0.104241	0.92267	0.08906	1.28	Down	0.043	XR_039164	ILMN_3284063	-	-	-
PREDICTED: misc_RNA (LOC390183), miscRNA.	-	0.647141	0.084917	0.280741	0.0831	1.29	Down	0.02156	XR_018242	ILMN_3199929	-	-	-
PREDICTED: misc_RNA (LOC390578), miscRNA.	-	0.769704	0.080468	0.551387	0.01805	1.16	Down	0.03817	XR_016734	ILMN_3287309	-	-	-
PREDICTED: misc_RNA (LOC390834), miscRNA.	-	0.179271	0.02648	0.317471	0.04911	1.1	Up	0.048	XR_037389	ILMN_3286875	-	-	-
PREDICTED: misc_RNA (LOC439953), miscRNA.	-	2.764247	0.106914	2.311305	0.11893	1.37	Down	0.02987	XR_017375	ILMN_3290298	-	-	-
PREDICTED: misc_RNA (LOC439953), miscRNA.	-	5.791729	0.097828	5.390077	0.08163	1.32	Down	0.01975	XR_017375	ILMN_3213792	-	-	-
PREDICTED: misc_RNA (LOC440063), miscRNA.	-	2.740825	0.13954	2.179696	0.15511	1.48	Down	0.03608	XR_018394	ILMN_3208715	-	-	-
PREDICTED: misc_RNA (LOC441073), miscRNA.	-	4.75637	0.093536	4.482866	0.03618	1.21	Down	0.03432	XR_018937	ILMN_3290199	-	-	-
PREDICTED: misc_RNA (LOC441481), miscRNA.	-	1.829551	0.173952	1.313491	0.06968	1.43	Down	0.03312	XR_038228	ILMN_3213925	-	-	-
PREDICTED: misc_RNA (LOC641727), miscRNA.	-	0.347853	0.009515	0.182581	0.0305	1.12	Down	0.00207	XR_018010	ILMN_3241118	-	-	-
PREDICTED: misc_RNA (LOC642357), miscRNA.	-	5.58161	0.051101	5.409116	0.02924	1.13	Down	0.02629	XR_018361	ILMN_3283155	-	-	-
PREDICTED: misc_RNA (LOC642975), miscRNA.	-	4.1236	0.095648	3.838209	0.02748	1.22	Down	0.02851	XR_036988	ILMN_3293146	-	-	-
PREDICTED: misc_RNA (LOC643358), miscRNA.	-	6.304713	0.088152	6.038777	0.03344	1.2	Down	0.03033	XR_038953	ILMN_3201480	-	-	-
PREDICTED: misc_RNA (LOC644563), miscRNA.	-	2.857482	0.148661	2.368513	0.08991	1.4	Down	0.03058	XR_018203	ILMN_3207122	-	-	-
PREDICTED: misc_RNA (LOC645173), miscRNA.	-	3.367049	0.115028	2.94205	0.08583	1.34	Down	0.02524	XR_017590	ILMN_3211132	-	-	-
PREDICTED: misc_RNA (LOC645630), miscRNA.	-	0.984572	0.077876	0.777264	0.02558	1.15	Down	0.04473	XR_039656	ILMN_3288529	-	-	-
PREDICTED: misc_RNA (LOC646527), miscRNA.	-	1.506223	0.077543	1.227378	0.07882	1.21	Down	0.04517	XR_016632	ILMN_3207738	-	-	-
PREDICTED: misc_RNA (LOC728263), miscRNA.	-	0.542457	0.080819	0.318198	0.02532	1.17	Down	0.03814	XR_037204	ILMN_3213176	-	-	-
PREDICTED: misc_RNA (LOC728368), miscRNA.	-	6.405642	0.093314	6.108643	0.07239	1.23	Down	0.04561	XR_015295	ILMN_3225941	-	-	-
PREDICTED: misc_RNA (LOC728408), miscRNA.	-	0.688491	0.062095	0.431808	0.05138	1.19	Down	0.01896	XR_039142	ILMN_3224340	-	-	-
PREDICTED: misc_RNA (LOC729142), miscRNA.	-	1.15832	0.055193	0.912764	0.02189	1.19	Down	0.00611	XR_038876	ILMN_3299579	-	-	-
PREDICTED: misc_RNA (LOC729236), miscRNA.	-	4.355495	0.112445	4.02453	0.06888	1.26	Down	0.04591	XR_016041	ILMN_3304130	-	-	-
PREDICTED: misc_RNA (LOC729298), miscRNA.	-	0.775097	0.080687	0.512208	0.06453	1.2	Down	0.04381	XR_039124	ILMN_3225843	-	-	-
PREDICTED: misc_RNA (LOC729340), miscRNA.	-	3.016436	0.103982	2.729946	0.04459	1.22	Down	0.04455	XR_039650	ILMN_3298215	-	-	-
PREDICTED: misc_RNA (LOC729816), miscRNA.	-	1.673589	0.096622	1.32441	0.08358	1.27	Down	0.03404	XR_042352	ILMN_3232696	-	-	-
PREDICTED: misc_RNA (RPS6P1), miscRNA.	-	0.268083	0.04543	0.406287	0.02866	1.1	Up	0.04217	XR_039076	ILMN_3297410	-	-	-

PREDICTED: protein phosphatase 1, regulatory (inhibitor) subunit 3E (PPP1R3E), mRNA.	-	0.545879	0.024875	0.669202	0.038	1.09	Up	0.03485	XM_927029	ILMN_1735064	-	-	-
PREDICTED: protein tyrosine phosphatase type IVA, member 2, transcript variant 5 (PTP4A2),	-	0.988633	0.018405	1.210339	0.08321	1.17	Up	0.04057	XM_944907	ILMN_1795697	-	-	-
PREDICTED: radical fringe homolog (Drosophila)	-	0.566452	0.032514	0.66938	0.01336	1.07	Up	0.02636	XM_941367	ILMN_1661427	-	-	-
PREDICTED: similar to 23 kD highly basic protein, transcript variant 1 (LOC728658), mRNA.	-	6.698273	0.034708	6.453739	0.0701	1.18	Down	0.02042	XM_939484	ILMN_3220792	-	-	-
PREDICTED: similar to 3-phosphoinositide dependent protein kinase 1 (hPDK1), transcript	-	0.242839	0.028925	0.11361	0.03473	1.09	Down	0.02883	XM_935348	ILMN_1773174	-	-	-
PREDICTED: similar to 40S ribosomal protein S29	-	6.518659	0.105793	6.222933	0.0362	1.23	Down	0.0383	XM_928180	ILMN_1739263	-	-	-
PREDICTED: similar to 40S ribosomal protein S3a (V-fos transformation effector protein)	-	0.549625	0.03805	0.40127	0.02705	1.11	Down	0.01913	XM_935589	ILMN_1745900	-	-	-
PREDICTED: similar to 60S ribosomal protein L12	-	3.341924	0.07527	3.073868	0.04488	1.2	Down	0.02226	XR_017614	ILMN_1772888	-	-	-
PREDICTED: similar to 60S ribosomal protein L14 (CAG-ISL 7), transcript variant 1 (LOC649821),	-	4.408347	0.05915	3.909784	0.1339	1.41	Down	0.01439	XM_942212	ILMN_1786242	-	-	-
PREDICTED: similar to 60S ribosomal protein L29 (Cell surface heparin-binding protein HIP)	-	4.257597	0.038128	4.03339	0.02702	1.17	Down	0.00301	XM_938790	ILMN_1675460	-	-	-
PREDICTED: similar to anaphase promoting complex subunit 1 (LOC100134261), mRNA.	-	0.16113	0.031322	0.264422	0.02722	1.07	Up	0.04721	XM_001720725	ILMN_3238945	-	-	-
PREDICTED: similar to CG11064-PA	-	1.173502	0.013195	1.315548	0.02772	1.1	Up	0.00359	XM_940588	ILMN_1799699	-	-	-
PREDICTED: similar to CG13731-PA	-	0.42258	0.031151	0.610787	0.04295	1.14	Up	0.01211	XM_926898	ILMN_1767932	-	-	-
PREDICTED: similar to Diacylglycerol O-acyltransferase 1 (Diglyceride acyltransferase)	-	0.22607	0.040725	0.36313	0.0319	1.1	Up	0.03806	XM_001127807	ILMN_1665621	-	-	-
PREDICTED: similar to erythrocyte membrane protein band 4.1 like 4B isoform 2 (LOC644763),	-	0.252173	0.030267	0.41088	0.03214	1.12	Up	0.01144	XM_927860	ILMN_1667402	-	-	-
PREDICTED: similar to family with sequence similarity 36, member A (LOC653377), mRNA.	-	1.869108	0.094725	1.495485	0.04217	1.3	Down	0.01132	XM_929420	ILMN_1772207	-	-	-
PREDICTED: similar to family with sequence similarity 70, member B (LOC652211), mRNA.	-	0.250923	0.034178	0.146866	0.01254	1.07	Down	0.02887	XM_941589	ILMN_1739483	-	-	-
PREDICTED: similar to FUS interacting protein (serine-arginine rich) 1 (LOC653884), mRNA.	-	2.430043	0.092607	2.157465	0.06107	1.21	Down	0.0493	XM_936240	ILMN_1658460	-	-	-
PREDICTED: similar to glutathione S-transferase alpha 3 (LOC647169), mRNA.	-	0.258409	0.040312	0.048905	0.04903	1.16	Down	0.0164	XM_941789	ILMN_1782937	-	-	-
PREDICTED: similar to GSQS6193	-	0.472699	0.059711	0.268579	0.02588	1.15	Down	0.02016	XM_001716832	ILMN_3187429	-	-	-
PREDICTED: similar to hCG1642995	-	0.234619	0.009732	0.161088	0.02336	1.05	Down	0.02714	XM_001723699	ILMN_3294302	-	-	-
PREDICTED: similar to hCG1646049	-	0.261493	0.0438	0.421933	0.02023	1.12	Up	0.0159	XM_001715992	ILMN_3185263	-	-	-
PREDICTED: similar to hCG1742852	-	0.211218	0.047552	0.342404	0.02101	1.1	Up	0.04507	XM_001717755	ILMN_3245093	-	-	-
PREDICTED: similar to hCG1809904	-	0.500666	0.023788	0.622613	0.0343	1.09	Up	0.02658	XM_001131091	ILMN_3244749	-	-	-
PREDICTED: similar to hCG1812832	-	3.684163	0.077535	3.386401	0.04294	1.23	Down	0.01524	XM_001722353	ILMN_3225211	-	-	-
PREDICTED: similar to hCG1997137, transcript variant 2 (LOC730029), mRNA.	-	1.461148	0.057852	1.176002	0.0381	1.22	Down	0.00624	XM_001724847	ILMN_3302456	-	-	-
PREDICTED: similar to hCG2027326	-	4.421507	0.092807	4.117809	0.05892	1.23	Down	0.03274	XM_001726077	ILMN_3275345	-	-	-
PREDICTED: similar to Heterogeneous nuclear ribonucleoprotein A1 (Helix-destabilizing protein) (Single-strand binding protein) (hnRNP core	-	4.034212	0.122437	3.67017	0.02996	1.29	Down	0.02776	XM_928473	ILMN_1691611	-	-	-
PREDICTED: similar to heterogeneous nuclear ribonucleoprotein A1 (LOC645385), mRNA.	-	4.377674	0.049321	4.159679	0.0533	1.16	Down	0.02395	XM_928427	ILMN_1720745	-	-	-
PREDICTED: similar to heterogeneous nuclear ribonucleoprotein C-like 1 (LOC649330), mRNA.	-	0.539429	0.02194	0.377519	0.03382	1.12	Down	0.00699	XM_001723218	ILMN_3244579	-	-	-
PREDICTED: similar to high-mobility group (nonhistone chromosomal) protein 1-like 10,	-	1.778623	0.113446	1.493215	0.01864	1.22	Down	0.04764	XM_001723189	ILMN_3265797	-	-	-
PREDICTED: similar to HIStone family member (his-72) (LOC391769), mRNA.	-	0.40981	0.02566	0.227509	0.05002	1.13	Down	0.01763	XM_001713901	ILMN_3278627	-	-	-
PREDICTED: similar to LHPE306, transcript variant 2 (LOC100134140), mRNA.	-	0.193508	0.005769	0.32826	0.03992	1.1	Up	0.0156	XM_001724811	ILMN_3200136	-	-	-
PREDICTED: similar to MAPK-interacting and spindle-stabilizing protein (LOC653171), mRNA.	-	2.139592	0.024981	1.906406	0.08674	1.18	Down	0.04158	XM_926322	ILMN_1715702	-	-	-
PREDICTED: similar to matrilin 2 precursor, transcript variant 1 (LOC285929), mRNA.	-	0.326521	0.0344	0.470156	0.02705	1.1	Up	0.01677	XM_209824	ILMN_1790173	-	3E+05	-
PREDICTED: similar to mCG7602	-	2.658485	0.138572	2.293605	0.02585	1.29	Down	0.0413	XM_001725693	ILMN_3288268	-	-	-
PREDICTED: similar to nuclease sensitive element binding protein 1 (LOC646531), mRNA.	-	4.589226	0.119414	4.188113	0.02557	1.32	Down	0.01673	XR_018197	ILMN_1669424	-	-	-

PREDICTED: similar to peptidylprolyl isomerase A isoform 1 (LOC402644), mRNA.	-	2.411102	0.100368	1.783313	0.11017	1.55	Down	0.00561	XM_938297	ILMN_1686811	-	-	-
PREDICTED: similar to pleckstrin homology domain containing, family M (with RUN domain) member 1; adapter protein 162, transcript variant 7	-	0.170304	0.01751	0.288157	0.01573	1.09	Up	0.00244	XM_935189	ILMN_1700427	-	-	-
PREDICTED: similar to Ras-related protein Rab-13	-	0.470263	0.034553	0.236966	0.03763	1.18	Down	0.00382	XM_927730	ILMN_1784005	-	-	-
PREDICTED: similar to ribosomal protein L10	-	0.25162	0.028627	0.071559	0.02494	1.13	Down	0.00318	XM_937850	ILMN_1694327	-	-	-
PREDICTED: similar to ribosomal protein L18a	-	3.995353	0.115736	3.656183	0.02544	1.27	Down	0.02872	XM_938382	ILMN_1801795	-	-	-
PREDICTED: similar to ribosomal protein L21	-	3.394972	0.104714	3.072554	0.00856	1.25	Down	0.02198	XM_001128906	ILMN_3299187	-	-	-
PREDICTED: similar to ribosomal protein L5, transcript variant 1 (LOC647436), mRNA.	-	1.152778	0.045756	0.939864	0.05097	1.16	Down	0.02089	XM_937113	ILMN_1772998	-	-	-
PREDICTED: similar to ribosomal protein L9	-	5.379962	0.056029	5.145566	0.0227	1.18	Down	0.0082	XM_940587	ILMN_1769277	-	-	-
PREDICTED: similar to ribosomal protein S12	-	6.573223	0.065524	6.354047	0.03707	1.16	Down	0.02693	XM_941155	ILMN_1687805	-	-	-
PREDICTED: similar to ribosomal protein S2, transcript variant 3 (LOC440589), mRNA.	-	5.910652	0.117008	5.509477	0.09022	1.32	Down	0.03486	XM_942424	ILMN_1652768	-	-	-
PREDICTED: similar to ribosomal protein S3a	-	1.250813	0.118247	0.922217	0.02517	1.26	Down	0.03474	XM_935588	ILMN_1679025	-	-	-
PREDICTED: similar to ribosomal protein S3a, transcript variant 4 (LOC648659), mRNA.	-	0.361781	0.040447	0.167951	0.05507	1.14	Down	0.02969	XM_944469	ILMN_1695899	-	-	-
PREDICTED: similar to scotin (LOC643043),	-	0.258327	0.008323	0.371458	0.01391	1.08	Up	0.00043	XM_926412	ILMN_1717232	-	-	-
PREDICTED: similar to serine/threonine/tyrosine interacting protein, transcript variant 1	-	2.309545	0.114894	1.924695	0.10491	1.31	Down	0.04822	XM_001125680	ILMN_1697024	-	-	-
PREDICTED: similar to SMT3 suppressor of mif two 3 homolog 2 (LOC390466), mRNA.	-	2.304977	0.052093	1.958149	0.04017	1.27	Down	0.00188	XM_372521	ILMN_1665781	-	4E+05	-
PREDICTED: similar to Sorbitol dehydrogenase (L-iditol 2-dehydrogenase) (LOC653381), mRNA.	-	1.305588	0.183375	0.800568	0.04853	1.42	Down	0.0374	XR_017364	ILMN_1692517	-	-	-
PREDICTED: similar to TBC1 domain family, member 3B (LOC653498), mRNA.	-	0.324724	0.048327	0.562463	0.05802	1.18	Up	0.01986	XM_927734	ILMN_1782958	-	-	-
PREDICTED: similar to tissue plasminogen activator (LOC100128998), mRNA.	-	0.224506	0.07213	0.439837	0.03196	1.16	Up	0.03421	XM_001717752	ILMN_3234254	-	-	-
PREDICTED: similar to transmembrane protein 106A, transcript variant 1 (LOC728772), mRNA.	-	2.402546	0.133062	2.02654	0.06775	1.3	Down	0.0454	XM_001133059	ILMN_1751941	-	-	-
PREDICTED: similar to Tubulin alpha-2 chain (Alpha-tubulin 2), transcript variant 5	-	1.016874	0.125363	0.651633	0.06672	1.29	Down	0.04223	XM_934471	ILMN_1765701	-	-	-
PREDICTED: similar to Williams Beuren syndrome chromosome region 19, transcript variant 5	-	1.500572	0.025606	1.629156	0.02353	1.09	Up	0.01012	XM_945628	ILMN_1744623	-	-	-
PREDICTED: similar to Zinc finger protein 406 (Protein ZFAT) (LOC654252), mRNA.	-	0.129253	0.033596	0.262269	0.02155	1.1	Up	0.01576	XM_942253	ILMN_1684977	-	-	-
PREDICTED: transmembrane protein 14D	-	3.708389	0.078876	3.382587	0.05004	1.25	Down	0.01302	XM_928242	ILMN_3244248	-	-	-
PREDICTED: Williams Beuren syndrome chromosome region 19, transcript variant 4	-	0.525832	0.025667	0.696943	0.02916	1.13	Up	0.00455	XM_943679	ILMN_1718311	-	-	-
Prefoldin subunit 5	PFDN5	5.062765	0.066143	4.615558	0.03553	1.36	Down	0.001	NM_002624	ILMN_1755536	Hs.655327	5204	12
Prefoldin subunit 5	PFDN5	4.928758	0.063996	4.623154	0.08965	1.24	Down	0.03223	NM_145897	ILMN_2356284	Hs.655327	5204	12
Presenilin 1	PSEN1	0.095269	0.022721	0.276851	0.03148	1.13	Up	0.00341	NM_007318	ILMN_1808548	Hs.3260	5663	14
Proline-rich protein BstNI subfamily 2	PRB2	0.139224	0.013931	0.330259	0.04941	1.14	Up	0.00984	NM_006248	ILMN_2192595	Hs.654486	7E+05	12
Proline/serine-rich coiled-coil 1	PSRC1	0.157796	0.04051	0.299267	0.03295	1.1	Up	0.03515	NM_001032290	ILMN_1801152	Hs.405925	84722	1
Propionyl Coenzyme A carboxylase, beta	PCCB	3.560843	0.066914	3.268537	0.05446	1.22	Down	0.01471	NM_000532	ILMN_1761010	Hs.63788	5096	3
Proteasome (prosome, macropain) subunit, alpha	PSMA2	1.26828	0.076441	0.86937	0.11956	1.32	Down	0.03071	NM_002787	ILMN_2058512	Hs.333786	5683	7
Proteasome (prosome, macropain) subunit, beta	PSMB1	6.212184	0.020151	5.98632	0.08362	1.17	Down	0.03927	NM_002793	ILMN_1789176	Hs.352768	5689	6
Protein arginine methyltransferase 1	PRMT1	3.155543	0.043588	2.899421	0.04427	1.19	Down	0.0062	NM_198319	ILMN_1692473	Hs.20521	3276	19
Protein phosphatase 1, regulatory (inhibitor)	PPP1R12C	0.82786	0.030591	1.026284	0.0651	1.15	Up	0.03291	NM_017607	ILMN_1685286	Hs.631579	54776	19
Protein phosphatase 1A (formerly 2C), magnesium-dependent, alpha isoform	PPM1A	0.991052	0.09707	0.675966	0.05118	1.24	Down	0.02838	AK124299	ILMN_1903159	Hs.130036	5494	14
Protein tyrosine phosphatase-like (proline instead of catalytic arginine), member A	PTPLA	1.166037	0.095595	0.743777	0.13071	1.34	Down	0.04025	NM_014241	ILMN_1725791	Hs.114062	9200	10
Protein tyrosine phosphatase, receptor type, O	PTPRO	0.691677	0.124037	0.269489	0.08033	1.34	Down	0.02892	NM_030667	ILMN_1720113	Hs.160871	5800	12
Proteinase 3	PRTN3	0.235128	0.062641	0.430972	0.02281	1.15	Up	0.02602	NM_002777	ILMN_1668460	Hs.928	5657	19
Protocadherin 9	PCDH9	0.250938	0.048003	0.410577	0.03652	1.12	Up	0.03819	NM_203487	ILMN_1668147	Hs.719302	5101	13
Pyridine nucleotide-disulphide oxidoreductase	PYROXD1	0.613776	0.077196	0.351423	0.0293	1.2	Down	0.01914	NM_024854	ILMN_3247826	Hs.709545	79912	12
Pyridoxal (pyridoxine, vitamin B6) phosphatase	PDXP	0.934363	0.045924	0.742395	0.03727	1.14	Down	0.01756	NM_020315	ILMN_1736441	Hs.632762	57026	22
Quinoid dihydropteridine reductase	QDPR	2.302128	0.077826	1.846942	0.05648	1.37	Down	0.00321	NM_000320	ILMN_1672443	Hs.75438	5860	4
RAB GTPase activating protein 1-like	RABGAP1L	0.534061	0.053929	0.272709	0.01311	1.2	Down	0.0033	NM_001035230	ILMN_1708721	Hs.585378	9910	1
RAB, member of RAS oncogene family-like 2A	RABL2A	0.115743	0.031192	0.23699	0.0216	1.09	Up	0.0187	NM_007082	ILMN_1666624	Hs.446425	11159	2

RAB, member of RAS oncogene family-like 4	RABL4	1.031928	0.059969	0.799393	0.05823	1.17	Down	0.03191	NM_006860	ILMN_1746492	Hs.415172	11020	22
RAB32, member RAS oncogene family	RAB32	2.306181	0.154844	1.740691	0.09781	1.48	Down	0.02145	NM_006834	ILMN_2115434	Hs.287714	10981	6
RAP2A, member of RAS oncogene family	RAP2A	1.473862	0.112879	1.093561	0.09544	1.3	Down	0.04218	NM_021033	ILMN_1677404	Hs.508480	5911	13
Raptor	KIAA1303	0.437091	0.073261	0.000628	0.07196	1.35	Down	0.00538	NM_020761	ILMN_1797816	Hs.133044	57521	17
Ras association (RalGDS/AF-6) domain family	RASSF6	0.526523	0.018081	0.388722	0.04893	1.1	Down	0.03845	NM_201431	ILMN_1745820	Hs.590920	2E+05	4
RAS p21 protein activator (GTPase activating	RASA1	0.296213	0.025546	0.4122	0.03216	1.08	Up	0.03019	NM_022650	ILMN_2292863	Hs.664080	5921	5
RAS protein activator like 3	RASAL3	0.178735	0.049237	0.32086	0.01553	1.1	Up	0.03317	NM_022904	ILMN_3238803	Hs.136979	64926	19
RAS-like, family 10, member B	RASL10B	0.203338	0.031242	0.311415	0.02308	1.08	Up	0.03189	NM_033315	ILMN_1788813	Hs.437035	91608	17
RCD1 required for cell differentiation1 homolog (S.	RQCD1	1.792162	0.046804	1.555589	0.0503	1.18	Down	0.01374	NM_005444	ILMN_2044085	Hs.148767	9125	2
RCE1 homolog, prenyl protein peptidase (S.	RCE1	2.417887	0.039222	2.643385	0.03479	1.17	Up	0.00509	NM_001032279	ILMN_1685002	Hs.654972	9986	11
REC8 homolog (yeast)	REC8	0.456133	0.04167	0.786807	0.12377	1.26	Up	0.04456	NM_005132	ILMN_1751886	Hs.419259	9985	14
Reticulon 4	RTN4	4.649702	0.067259	4.271065	0.09523	1.3	Down	0.01752	NM_007008	ILMN_1730611	Hs.704007	57142	2
Retinoid X receptor, alpha	RXRA	3.405925	0.087625	3.094433	0.05262	1.24	Down	0.02258	NM_002957	ILMN_1687315	Hs.590886	6256	9
Retinol dehydrogenase 16 (all-trans)	RDH16	0.159284	0.043971	0.3141	0.01664	1.11	Up	0.01655	NM_003708	ILMN_1810000	Hs.134958	8608	12
Rho GTPase activating protein 30	ARHGAP30	0.718592	0.066996	0.439063	0.06775	1.21	Down	0.02616	NM_181720	ILMN_2408851	Hs.389374	3E+05	1
Rho guanine nucleotide exchange factor (GEF) 17	ARHGEF17	0.325633	0.031043	0.460744	0.0351	1.1	Up	0.02793	NM_014786	ILMN_1754562	Hs.533719	9828	11
Rho-associated, coiled-coil containing protein	ROCK2	6.894477	0.056741	7.0954	0.03634	1.15	Up	0.02457	NM_004850	ILMN_2058337	Hs.591600	9475	2
Ribose 5-phosphate isomerase A	RPIA	2.089016	0.068949	1.866625	0.05427	1.17	Down	0.0444	NM_144563	ILMN_1714809	Hs.469264	22934	2
Ribosomal L1 domain containing 1	RSL1D1	3.355785	0.067006	3.157952	0.03796	1.15	Down	0.0424	NM_015659	ILMN_1723729	Hs.401842	26156	16
Ribosomal protein L12	RPL12	4.429919	0.060375	4.197531	0.05547	1.17	Down	0.02979	NM_000976	ILMN_1653469	Hs.408054	6136	9
Ribosomal protein L17 pseudogene 7	RPL17P7	5.210093	0.111535	4.841854	0.05707	1.29	Down	0.02598	XM_001717161	ILMN_3279306	Hs.485081	1E+08	1
Ribosomal protein L17 pseudogene 7	RPL17P7	5.209029	0.118318	4.862026	0.03498	1.27	Down	0.03066	XM_001717156	ILMN_3202734	Hs.485081	1E+08	1
Ribosomal protein L18a	RPL18A	6.458175	0.07223	6.215582	0.01918	1.18	Down	0.01755	NM_000980	ILMN_2141444	Hs.337766	6142	19
Ribosomal protein L21	RPL21	4.673684	0.103403	4.343536	0.03361	1.26	Down	0.02291	NM_000982	ILMN_2290808	Hs.381123	6144	13
Ribosomal protein L22	RPL22	3.391003	0.095087	3.043956	0.01725	1.27	Down	0.01149	NM_000983	ILMN_2079386	Hs.515329	6146	1
Ribosomal protein L23	RPL23	4.295062	0.139668	3.926672	0.03853	1.29	Down	0.04392	NM_000978	ILMN_1755115	Hs.406300	9349	17
Ribosomal protein L23a	RPL23A	2.311799	0.089299	2.020536	0.05162	1.22	Down	0.0302	NM_000984	ILMN_1788607	Hs.419463	6147	17
ribosomal protein L23a pseudogene 7	-	1.5533	0.052885	1.333523	0.06083	1.16	Down	0.03434	NR_000029	ILMN_2222750	-	1E+05	-
Ribosomal protein L26	RPL26	4.385169	0.088429	4.104642	0.05961	1.21	Down	0.03903	NM_000987	ILMN_1731546	Hs.644794	6154	17
Ribosomal protein L26-like 1	RPL26L1	3.211314	0.154343	2.736094	0.05964	1.39	Down	0.02835	NM_016093	ILMN_2110532	Hs.546390	51121	5
Ribosomal protein L32	RPL32	0.14088	0.031581	0.25769	0.01485	1.08	Up	0.01548	NM_001007074	ILMN_1782167	Hs.265174	6161	3
ribosomal protein L35 pseudogene	-	0.343287	0.030916	0.489228	0.03964	1.11	Up	0.02722	XM_372704	ILMN_1666477	-	4E+05	-
ribosomal protein L36a pseudogene 49	-	5.819063	0.099962	5.413136	0.01442	1.32	Down	0.00697	XM_208185	ILMN_3274904	-	3E+05	-
Ribosomal protein L36a-like	RPL36AL	5.414143	0.065527	5.219956	0.04229	1.14	Down	0.04717	NM_001001	ILMN_2189936	Hs.444749	6166	14
ribosomal protein L5 pseudogene 7	-	1.70441	0.126212	1.260869	0.10657	1.36	Down	0.03629	XM_292963	ILMN_1679280	-	3E+05	-
Ribosomal protein S10	RPS10	6.358938	0.065671	6.153163	0.02561	1.15	Down	0.02666	NM_001014	ILMN_1686954	Hs.645317	6204	6
Ribosomal protein S10 pseudogene 13	RPS10P13	5.038033	0.13224	4.687126	0.04484	1.28	Down	0.04572	XR_017607	ILMN_3210538	Hs.573256	6E+05	6
Ribosomal protein S12	RPS12	6.973998	0.024871	6.774096	0.03466	1.15	Down	0.00337	NM_001016	ILMN_1782621	Hs.546289	6206	6
ribosomal protein S12 pseudogene 4	-	5.330086	0.034311	5.050833	0.06728	1.21	Down	0.01012	XM_372926	ILMN_3293367	-	4E+05	-
Ribosomal protein S15a	RPS15A	5.388678	0.077546	5.164165	0.04134	1.17	Down	0.04321	NM_001019	ILMN_2337241	Hs.370504	6210	16
Ribosomal protein S15a pseudogene 11	RPS15AP11	3.145019	0.076228	2.861824	0.07843	1.22	Down	0.04125	XM_927887	ILMN_3290353	Hs.512342	6E+05	1
Ribosomal protein S20	RPS20	6.070436	0.066473	5.697874	0.0584	1.29	Down	0.00562	NM_001023	ILMN_1701596	Hs.8102	6224	8
Ribosomal protein S27	RPS27	7.03007	0.067152	6.804607	0.05962	1.17	Down	0.04586	NM_001030	ILMN_1660498	Hs.546291	6232	1
Ribosomal protein S28	RPS28	5.042302	0.071137	4.773786	0.05291	1.2	Down	0.02313	NM_001031	ILMN_1651228	Hs.719103	6234	19
Ribosomal protein S6	RPS6	5.772181	0.066173	5.444406	0.04476	1.26	Down	0.00634	NM_001010	ILMN_1656791	Hs.408073	6194	9
Ribosomal protein S9	RPS9	3.979637	0.10734	3.636455	0.05779	1.27	Down	0.03055	NM_001013	ILMN_2038772	Hs.546288	6203	19
Ribosome binding protein 1 homolog 180kDa	RRBP1	5.017629	0.00627	4.829008	0.05829	1.14	Down	0.0182	NM_001042576	ILMN_2360784	Hs.472213	6238	20
Ring finger and WD repeat domain 3	RFWD3	1.613783	0.032287	1.767982	0.05086	1.11	Up	0.04293	NM_018124	ILMN_1687107	Hs.567525	55159	16
Ring-box 1	RBX1	5.144692	0.106507	4.82615	0.0254	1.25	Down	0.02701	NM_014248	ILMN_1666670	Hs.474949	9978	22
RNA binding motif protein 12	RBM12	0.429004	0.057448	0.214936	0.05838	1.16	Down	0.03993	NM_006047	ILMN_1797698	Hs.246413	10137	20
RNA binding motif protein, X-linked pseudogene 3	-	0.471931	0.049716	0.294676	0.04728	1.13	Down	0.04157	NR_002197	ILMN_2119421	-	1E+05	-
ROD1 regulator of differentiation 1 (S. pombe)	ROD1	3.405639	0.109566	2.911022	0.09185	1.41	Down	0.01347	NM_005156	ILMN_2117223	Hs.269988	9991	9
S-phase kinase-associated protein 1	SKP1	3.485485	0.093178	3.198755	0.03874	1.22	Down	0.02951	NM_006930	ILMN_1711766	Hs.171626	6500	5
S100 calcium binding protein A4	S100A4	4.01894	0.318687	2.916436	0.29265	2.15	Down	0.0436	NM_019554	ILMN_1684306	Hs.654444	6275	1
S100 calcium binding protein A4	S100A4	4.415591	0.260482	3.405492	0.26863	2.01	Down	0.0356	NM_019554	ILMN_1688780	Hs.654444	6275	1
SEC13 homolog (S. cerevisiae)	SEC13	2.762064	0.047543	2.602393	0.01555	1.12	Down	0.01879	NM_001136232	ILMN_3223181	Hs.166924	6396	3
SEC62 homolog (S. cerevisiae)	SEC62	0.707792	0.09706	0.40599	0.01334	1.23	Down	0.02165	NM_003262	ILMN_1762003	Hs.622596	7095	3
Secreted phosphoprotein 1	SPP1	6.345564	0.121342	5.32701	0.26381	2.03	Down	0.01271	NM_000582	ILMN_1651354	Hs.313	6696	4
Secreted phosphoprotein 1	SPP1	5.979136	0.159881	5.061874	0.27991	1.89	Down	0.02935	NM_001040058	ILMN_2374449	Hs.313	6696	4
Secretory carrier membrane protein 1	SCAMP1	0.398741	0.037897	0.254188	0.02413	1.11	Down	0.0182	NM_004866	ILMN_1729058	Hs.482587	9522	5
Selenoprotein N, 1	SEPN1	1.795628	0.087982	1.49693	0.07129	1.23	Down	0.03866	NM_206926	ILMN_1760890	Hs.568578	57190	1
Serine incorporator 3	SERINC3	1.342451	0.148757	0.884313	0.10423	1.37	Down	0.04515	NM_006811	ILMN_1713752	Hs.272168	10955	20

Serine peptidase inhibitor, Kazal type 9	SPINK9	0.142831	0.040452	0.347704	0.01477	1.15	Up	0.00314	NM_001040433	ILMN_2045351	Hs.631798	6E+05	5
Serine/threonine kinase 17a	STK17A	0.629668	0.032335	0.833415	0.05384	1.15	Up	0.0176	NM_004760	ILMN_1776428	Hs.709489	9263	7
SH2B adaptor protein 1	SH2B1	0.875331	0.018835	1.008306	0.03588	1.1	Up	0.01679	NM_015503	ILMN_2061185	Hs.15744	25970	16
Sialophorin	SPN	1.007011	0.055811	1.197557	0.03463	1.14	Up	0.0273	NM_001030288	ILMN_1801040	Hs.632188	6693	16
sideroflexin 4	-	1.708129	0.054767	1.427179	0.07936	1.21	Down	0.02685	NM_213650	ILMN_2363361	-	1E+05	-
Signal recognition particle 19kDa	SRP19	3.23381	0.143818	2.816397	0.07339	1.34	Down	0.04148	NM_003135	ILMN_2192032	Hs.637001	6728	5
Signal recognition particle 9-like 1	SRP9L1	4.586916	0.104406	4.281682	0.05812	1.24	Down	0.04323	XM_927451	ILMN_1763404	Hs.632087	7E+05	10
Similar to CEACAM5 protein	FLJ41856	0.278425	0.035085	0.385537	0.02613	1.08	Up	0.04989	XM_371176	ILMN_1749266	Hs.446909	4E+05	19
Similar to CG32736-PA	LOC440957	2.012179	0.097233	1.674378	0.03994	1.26	Down	0.01829	NM_001124767	ILMN_3245869	Hs.660577	4E+05	3
Similar to hCG2029803	LOC1001295	0.270258	0.047985	0.47769	0.04297	1.15	Up	0.01813	XM_001717800	ILMN_3182735	Hs.712708	1E+08	6
Similar to hCG2045487	LOC283440	0.299722	0.051034	0.437966	0.01937	1.1	Up	0.04452	XM_211040	ILMN_1659959	Hs.658536	3E+05	12
Similar to hCG40442	LOC339760	0.348271	0.032042	0.458063	0.02653	1.08	Up	0.03859	XM_295058	ILMN_1651288	Hs.98984	3E+05	2
Similar to LOC339047 protein	LOC1001326	0.356087	0.056002	0.549765	0.0413	1.14	Up	0.03185	XM_001724221	ILMN_3246585	Hs.718878	1E+08	16
Similar to Lsm3 protein	LOC647302	4.129646	0.075884	3.889692	0.04597	1.18	Down	0.03536	XR_019555	ILMN_3275590	Hs.646813	6E+05	2
similar to PRO2738	-	0.285783	0.02182	0.386902	0.02757	1.07	Up	0.0282	XM_376822	ILMN_1767980	-	4E+05	-
Similar to spermine synthase	LOC646347	4.622985	0.064861	4.347514	0.05776	1.21	Down	0.01927	XR_017680	ILMN_3198367	Hs.634016	6E+05	1
Sirtuin (silent mating type information regulation 2 homolog) 2 (S. cerevisiae)	SIRT2	0.622735	0.065249	0.424309	0.04613	1.15	Down	0.0476	NM_012237	ILMN_1723494	Hs.466693	22933	19
SLAIN motif family, member 2	SLAIN2	0.143697	0.033255	0.295566	0.03991	1.11	Up	0.02652	NM_020846	ILMN_3234775	Hs.479677	57606	4
Small EDRK-rich factor 1A (telomeric)	SERF1A	0.236455	0.015016	0.323247	0.03031	1.06	Up	0.04256	NM_021967	ILMN_2280630	Hs.559428	8293	5
Small EDRK-rich factor 2	SERF2	5.521318	0.019509	5.394648	0.04584	1.09	Down	0.04392	NM_001018108	ILMN_1789136	Hs.424126	10169	15
Sodium channel, voltage-gated, type I, beta	SCN1B	0.222586	0.023868	0.347382	0.0177	1.09	Up	0.00569	NM_199037	ILMN_1767666	Hs.436646	6324	19
Sodium channel, voltage-gated, type III, beta	SCN3B	0.72551	0.040504	0.85505	0.01067	1.09	Up	0.02131	NM_018400	ILMN_2412822	Hs.4865	55800	11
Solute carrier family 13 (sodium/sulfate	SLC13A1	0.265788	0.019683	0.489576	0.04291	1.17	Up	0.00319	NM_022444	ILMN_2204430	Hs.489849	6561	7
Solute carrier family 25, member 42	SLC25A42	1.15595	0.017716	1.356035	0.06989	1.15	Up	0.03221	NM_178526	ILMN_2222880	Hs.303669	3E+05	19
Solute carrier family 29 (nucleoside transporters),	SLC29A4	0.44198	0.036656	0.577474	0.01868	1.1	Up	0.01654	NM_153247	ILMN_1801377	Hs.4302	2E+05	7
Solute carrier family 30 (zinc transporter), member	SLC30A5	2.296084	0.128116	1.933278	0.03862	1.29	Down	0.03504	NM_022902	ILMN_1709728	Hs.631975	64924	5
Solute carrier family 38, member 6	SLC38A6	1.84733	0.270342	1.054783	0.03498	1.73	Down	0.02707	NM_153811	ILMN_1696622	Hs.200738	1E+05	14
Solute carrier family 44, member 4	SLC44A4	0.271346	0.016189	0.348328	0.02404	1.05	Up	0.03773	NM_025257	ILMN_1730977	Hs.335355	80736	6
Solute carrier family 5 (iodide transporter), member	SLC5A8	1.833749	0.038643	2.027835	0.03555	1.14	Up	0.01013	NM_145913	ILMN_1811221	Hs.444536	2E+05	12
Solute carrier family 5 (sodium-dependent vitamin	SLC5A6	5.659733	0.027849	5.442973	0.04538	1.16	Down	0.00657	NM_021095	ILMN_1741054	Hs.435735	8884	2
Solute carrier family 5 (sodium/glucose	SLC5A2	0.483391	0.01473	0.611525	0.04888	1.09	Up	0.0459	NM_003041	ILMN_1666972	Hs.709195	6524	16
Solute carrier family 6, member 18	SLC6A18	0.017762	0.054709	0.270308	0.03022	1.19	Up	0.0068	NM_182632	ILMN_1813964	Hs.213284	3E+05	5
Solute carrier family 7, (neutral amino acid transporter, y+ system) member 10	SLC7A10	0.42843	0.049692	0.666255	0.06507	1.18	Up	0.02717	NM_019849	ILMN_1681087	Hs.58679	56301	19
Sorting nexin 5	SNX5	1.467499	0.070408	1.159972	0.07701	1.24	Down	0.02571	NM_014426	ILMN_1709772	Hs.316890	27131	20
Sp7 transcription factor	SP7	0.140829	0.027365	0.276881	0.03575	1.1	Up	0.02334	NM_152860	ILMN_1689461	Hs.209402	1E+05	12
Spectrin, beta, non-erythrocytic 5	SPTBN5	0.775597	0.05538	0.952799	0.02523	1.13	Up	0.02691	NM_016642	ILMN_1807609	Hs.709819	51332	15
Spermine oxidase	SMOX	0.132981	0.036269	0.25325	0.02274	1.09	Up	0.03078	NM_175842	ILMN_2280707	Hs.433337	54498	20
Spermine synthase	SMS	5.247495	0.056161	4.942984	0.06334	1.23	Down	0.0114	NM_004595	ILMN_1694305	Hs.715555	6611	X
Sphingomyelin phosphodiesterase 1, acid	SMPD1	1.831087	0.148084	1.266947	0.13722	1.48	Down	0.0314	NM_001007593	ILMN_1757370	Hs.498173	6609	11
Sphingomyelin phosphodiesterase 1, acid	SMPD1	1.484894	0.107255	1.12739	0.06652	1.28	Down	0.02986	NM_000543	ILMN_1741684	Hs.498173	6609	11
Sphingosine kinase 1	SPHK1	0.530078	0.047628	0.36821	0.03924	1.12	Down	0.03942	NM_021972	ILMN_2357134	Hs.68061	8877	17
Spindlin family, member 3	SPIN3	0.382191	0.05072	0.173143	0.05214	1.16	Down	0.02828	NM_001010862	ILMN_1801045	Hs.522672	2E+05	X
Sprouty homolog 4 (Drosophila)	SPRY4	0.393605	0.020029	0.618358	0.02563	1.17	Up	0.00045	NM_030964	ILMN_1688811	Hs.323308	81848	5
ST3 beta-galactoside alpha-2,3-sialyltransferase 5	ST3GAL5	0.428763	0.085664	0.141346	0.01853	1.22	Down	0.01684	NM_001042437	ILMN_2388701	Hs.415117	8869	2
ST6 (alpha-N-acetyl-neuraminyl-2,3-beta-galactosyl-1,3)-N-acetylgalactosaminide alpha-2,6-	ST6GALNAC	0.418732	0.056231	0.229271	0.04491	1.14	Down	0.03891	NM_175039	ILMN_1687857	Hs.3972	27090	9
Sterile alpha motif domain containing 10	SAMD10	0.407041	0.01153	0.518485	0.01209	1.08	Up	0.00055	NM_080621	ILMN_1767801	Hs.27189	1E+05	20
Sterile alpha motif domain containing 4A	SAMD4A	0.287051	0.04686	0.015063	0.03103	1.21	Down	0.00288	NM_015589	ILMN_2119297	Hs.98259	23034	14
Succinate dehydrogenase complex, subunit C, integral membrane protein, 15kDa	SDHC	2.556756	0.061175	2.151539	0.08486	1.32	Down	0.00823	NM_003001	ILMN_1746241	Hs.444472	6391	1
Succinate dehydrogenase complex, subunit C, integral membrane protein, 15kDa	SDHC	2.500823	0.029609	2.288989	0.04455	1.16	Down	0.00745	XM_001713656	ILMN_3248403	Hs.444472	6391	1
Sulfotransferase family, cytosolic, 2B, member 1	SULT2B1	0.920878	0.047688	0.683652	0.02311	1.18	Down	0.00421	NM_004605	ILMN_1705674	Hs.369331	6820	19
Superoxide dismutase 1, soluble	SOD1	6.669206	0.036615	6.392893	0.08753	1.21	Down	0.0269	NM_000454	ILMN_1662438	Hs.443914	6647	21
T-complex 1	TCP1	3.531388	0.139546	3.082157	0.05085	1.37	Down	0.02326	NM_030752	ILMN_1776347	Hs.363137	6950	6
T-SNARE domain containing 1	TSNARE1	0.108072	0.015883	0.283737	0.0253	1.13	Up	0.00107	NM_145003	ILMN_1781027	Hs.370931	2E+05	8
Tandem C2 domains, nuclear	TC2N	0.5459	0.093269	0.287528	0.02171	1.2	Down	0.03567	NM_152332	ILMN_1734596	Hs.510262	1E+05	14
TBC1 domain family, member 16	TBC1D16	0.251884	0.026447	0.070757	0.05482	1.13	Down	0.02477	NM_019020	ILMN_1758457	Hs.369819	1E+05	17
TBC1 domain family, member 3C	TBC1D3C	0.49109	0.04057	0.638239	0.04157	1.11	Up	0.04447	NM_001001418	ILMN_1693802	Hs.617010	4E+05	17
Tectonic family member 2	TCTN2	0.120265	0.045546	0.267687	0.0186	1.11	Up	0.02411	NM_024809	ILMN_1706345	Hs.167165	79867	12

Tetraspanin 31	TSPAN31	2.590919	0.077969	2.331434	0.0271	1.2	Down	0.01998	NM_005981	ILMN_1725079	Hs.632708	6302	12
Tetratricopeptide repeat domain 15	TTC15	1.584439	0.03691	1.442629	0.04424	1.1	Down	0.04904	NM_016030	ILMN_1693317	Hs.252713	51112	2
THAP domain containing 6	THAP6	0.885854	0.036456	0.703231	0.01764	1.13	Down	0.00406	NM_144721	ILMN_1661424	Hs.479971	2E+05	4
Thiopurine S-methyltransferase	TPMT	1.025479	0.049854	0.772076	0.08261	1.19	Down	0.03925	NM_000367	ILMN_1740185	Hs.444319	7172	6
Thioredoxin	TXN	4.855333	0.092629	4.522541	0.09741	1.26	Down	0.04808	NM_003329	ILMN_1680314	Hs.435136	7295	9
Thymidine phosphorylase	TYMP	4.594135	0.151493	4.00615	0.16927	1.5	Down	0.0413	NM_001953	ILMN_2109708	Hs.592212	1890	22
Thymidine phosphorylase	TYMP	1.097137	0.129416	0.712192	0.06721	1.31	Down	0.03855	NM_001113756	ILMN_3223126	Hs.592212	1890	22
Thymidine phosphorylase	TYMP	0.862472	0.098576	0.546424	0.0639	1.24	Down	0.03603	NM_001113755	ILMN_3297126	Hs.592212	1890	22
Thymidine phosphorylase	TYMP	0.66049	0.08946	0.426967	0.03308	1.18	Down	0.0499	NM_001953	ILMN_1690939	Hs.592212	1890	22
Thyroid hormone receptor interactor 11	TRIP11	2.989989	0.043239	2.835954	0.03132	1.11	Down	0.02788	NM_004239	ILMN_1658144	Hs.632339	9321	14
Topoisomerase (DNA) II alpha 170kDa	TOP2A	0.445084	0.031345	0.26271	0.03675	1.13	Down	0.00922	NM_001067	ILMN_1686097	Hs.156346	7153	17
TRAF and TNF receptor associated protein	TTRAP	0.56981	0.078894	0.346625	0.02951	1.17	Down	0.03805	NM_016614	ILMN_2183216	Hs.716397	51567	6
Transcribed locus	-	0.97726	0.043613	0.640613	0.06268	1.26	Down	0.00453	W25998	ILMN_1835017	Hs.633892	-	1
Transcribed locus	-	0.369718	0.070729	0.092206	0.05487	1.21	Down	0.02111	BG207842	ILMN_1903524	Hs.581828	-	4
Transcribed locus	-	0.310538	0.051283	0.077316	0.04429	1.18	Down	0.01377	BE504445	ILMN_1911361	Hs.436679	-	2
Transcribed locus	-	0.049436	0.031462	0.278307	0.03599	1.17	Up	0.00304	AW237220	ILMN_1912822	Hs.649593	-	5
Transcribed locus	-	0.545845	0.053888	0.773991	0.03375	1.17	Up	0.01153	BF515737	ILMN_1862105	Hs.649222	-	7
Transcribed locus	-	0.127643	0.013507	0.348848	0.08058	1.17	Up	0.03523	CD244435	ILMN_1832949	Hs.283139	-	1
Transcribed locus	-	0.502798	0.044416	0.718632	0.06589	1.16	Up	0.03482	DB296040	ILMN_1837739	Hs.575812	-	1
Transcribed locus	-	0.649558	0.077326	0.852443	0.0144	1.15	Up	0.0418	AW073896	ILMN_1912288	Hs.537603	-	1
Transcribed locus	-	0.301363	0.047682	0.100189	0.01599	1.15	Down	0.00712	BM673749	ILMN_1879135	Hs.555208	-	4
Transcribed locus	-	0.216105	0.025469	0.414273	0.04358	1.15	Up	0.00775	DB311601	ILMN_1890421	Hs.578394	-	15
Transcribed locus	-	0.42552	0.047723	0.235915	0.03674	1.14	Down	0.01986	BX115952	ILMN_1825773	Hs.129160	-	1
Transcribed locus	-	0.185424	0.035302	0.362036	0.03961	1.13	Up	0.01583	BX100285	ILMN_1848906	Hs.208715	-	9
Transcribed locus	-	0.183996	0.040714	0.356227	0.04284	1.13	Up	0.02684	DB038929	ILMN_1877025	Hs.578895	-	16
Transcribed locus	-	0.314306	0.048017	0.151526	0.02595	1.12	Down	0.02456	BM668469	ILMN_1893026	Hs.566514	-	6
Transcribed locus	-	0.218861	0.029419	0.362543	0.01965	1.1	Up	0.00664	AA972966	ILMN_1861090	Hs.541273	-	19
Transcribed locus	-	0.364496	0.051846	0.504717	0.01896	1.1	Up	0.04408	BX102164	ILMN_1917089	Hs.443067	-	15
Transcribed locus	-	0.163102	0.04559	0.29678	0.01039	1.1	Up	0.02884	AL832704	ILMN_1850594	Hs.353387	-	21
Transcribed locus	-	0.293225	0.039813	0.172599	0.02088	1.09	Down	0.03638	BX119561	ILMN_1902604	Hs.571207	-	6
Transcribed locus, weakly similar to XP_609073.4 PREDICTED: similar to laminin, beta 2 [Bos taurus]	-	0.035821	0.03762	0.261992	0.04105	1.17	Up	0.00664	CB305782	ILMN_1827287	Hs.633202	-	3
Transcription elongation factor A (SII)-like 8	TCEAL8	2.144026	0.070846	1.86647	0.06544	1.21	Down	0.02814	NM_001006684	ILMN_2402272	Hs.389734	90843	X
Transformer 2 beta homolog (Drosophila)	TRA2B	3.446757	0.062623	3.130463	0.08645	1.25	Down	0.02518	NM_004593	ILMN_1742798	Hs.533122	6434	3
Transient receptor potential cation channel,	TRPM3	0.066902	0.057259	0.253209	0.03517	1.14	Up	0.03232	NM_001007470	ILMN_2302983	Hs.47288	80036	9
Translocator protein (18kDa)	TSPO	4.024208	0.213852	3.433236	0.0984	1.51	Down	0.04588	NM_000714	ILMN_1681679	Hs.202	706	22
Translocator protein (18kDa)	TSPO	1.941029	0.149536	1.549509	0.02126	1.31	Down	0.04109	NM_000714	ILMN_2260991	Hs.202	706	22
Transmembrane protein 132E	TMEM132E	0.451228	0.010262	0.602566	0.04062	1.11	Up	0.0112	NM_207313	ILMN_1772514	Hs.310482	1E+05	17
Transmembrane protein 141	TMEM141	3.22903	0.049115	2.938638	0.09349	1.22	Down	0.0333	NM_032928	ILMN_1774066	Hs.356744	85014	9
Transmembrane protein 156	TMEM156	1.041061	0.021423	1.120875	0.01472	1.06	Up	0.02193	NM_024943	ILMN_2095660	Hs.374147	80008	4
Transmembrane protein 167A	TMEM167A	2.50974	0.052737	2.228808	0.10018	1.21	Down	0.04772	NM_174909	ILMN_3251560	Hs.355606	2E+05	5
Transmembrane protein 208	TMEM208	2.957165	0.017021	2.698867	0.06963	1.2	Down	0.01132	NM_014187	ILMN_3239055	Hs.433203	29100	16
Transmembrane protein 219	TMEM219	2.042676	0.069406	1.775064	0.07763	1.2	Down	0.04234	NM_001083613	ILMN_1737644	Hs.460574	1E+05	16
Transmembrane protein 223	TMEM223	0.399153	0.067867	0.140667	0.05405	1.2	Down	0.02466	NM_001080501	ILMN_2263144	Hs.530463	79064	11
Transmembrane protein 68	TMEM68	0.467736	0.091543	0.731647	0.0136	1.2	Up	0.02912	NM_152417	ILMN_1702244	Hs.420076	1E+05	8
Transmembrane protein 77	TMEM77	1.034673	0.023719	0.779205	0.10096	1.19	Down	0.0489	NM_178454	ILMN_1808634	Hs.485606	1E+05	1
Transmembrane protein 85	TMEM85	4.7907	0.105873	4.459294	0.07962	1.26	Down	0.04642	NM_016454	ILMN_1798874	Hs.250905	51234	15
Triggering receptor expressed on myeloid cells 2	TREM2	1.615603	0.208982	0.805926	0.0459	1.75	Down	0.00914	NM_018965	ILMN_1701248	Hs.435295	54209	6
Trimethyllysine hydroxylase, epsilon	TMLHE	0.235272	0.043543	0.076074	0.01954	1.12	Down	0.0157	NM_018196	ILMN_1683575	Hs.133321	55217	X
Tripartite motif-containing 26	TRIM26	2.372978	0.084504	2.667343	0.05874	1.23	Up	0.02879	NM_003449	ILMN_1738704	Hs.485041	7726	6
TRNA splicing endonuclease 15 homolog (S.	TSEN15	0.733303	0.05299	0.443911	0.07344	1.22	Down	0.01871	NM_001127394	ILMN_3225244	Hs.548197	1E+05	1
TRNA splicing endonuclease 2 homolog (S.	TSEN2	0.483585	0.027682	0.341778	0.04107	1.1	Down	0.02869	NM_025265	ILMN_1746393	Hs.335550	80746	3
TROVE domain family, member 2	TROVE2	3.203743	0.049073	2.954117	0.04831	1.19	Down	0.01103	NM_001042369	ILMN_2311518	Hs.288178	6738	1
Tryptophan rich basic protein	WRB	1.34676	0.061408	0.957039	0.05446	1.31	Down	0.00316	NM_004627	ILMN_1695092	Hs.198308	7485	21
TSPY-like 1	TSPYL1	2.291676	0.048476	2.011495	0.06408	1.21	Down	0.01303	NM_003309	ILMN_1779014	Hs.458358	7259	6
Tubulin, alpha 1a	TUBA1A	2.937322	0.180639	2.383032	0.04406	1.47	Down	0.0246	NM_006009	ILMN_1742981	Hs.654422	7846	12
Tubulin, alpha 1b	TUBA1B	5.922971	0.187538	5.38529	0.10134	1.45	Down	0.04514	NM_006082	ILMN_1800261	Hs.524390	10376	12
Tumor protein p53 inducible protein 3	TP53I3	1.644954	0.078899	1.320194	0.08246	1.25	Down	0.02935	NM_147184	ILMN_2358919	Hs.50649	9540	2
Ubiquilin-like	UBQLNL	0.313652	0.031142	0.462065	0.04118	1.11	Up	0.02826	NM_145053	ILMN_1748907	Hs.10688	1E+05	11
Ubiquitin A-52 residue ribosomal protein fusion	UBA52	6.607006	0.040838	6.37756	0.03705	1.17	Down	0.00594	NM_001033930	ILMN_1782977	Hs.5308	7311	19
Ubiquitin specific peptidase 28	USP28	0.47016	0.020434	0.588885	0.02155	1.09	Up	0.00713	NM_020886	ILMN_1794306	Hs.503891	57646	11
Ubiquitin-conjugating enzyme E2 variant 1	UBE2V1	0.58597	0.043933	0.729067	0.02454	1.1	Up	0.02942	NM_021988	ILMN_1665862	Hs.420529	7335	20

Ubiquitin-like modifier activating enzyme 3	UBA3	2.602307	0.067432	2.299042	0.05553	1.23	Down	0.01328	NM_198195	ILMN_2324157	Hs.154320	9039	3
UDP-glucose dehydrogenase	UGDH	1.055514	0.077715	0.785581	0.05373	1.21	Down	0.02891	NM_003359	ILMN_1729563	Hs.572518	7358	4
UFM1-specific peptidase 2	UFSP2	2.363836	0.059086	2.110014	0.01591	1.19	Down	0.00602	NM_018359	ILMN_1756311	Hs.713548	55325	4
Vacuolar protein sorting 41 homolog (S.	VPS41	0.614279	0.049992	0.419122	0.03829	1.14	Down	0.02114	NM_014396	ILMN_1796075	Hs.592184	27072	7
Vanin 3	VNN3	1.694791	0.017877	2.31945	0.15777	1.54	Up	0.00768	NM_001024460	ILMN_1804935	Hs.183656	55350	6
Vesicle amine transport protein 1 homolog (T.	VAT1	2.372377	0.119246	1.917664	0.1019	1.37	Down	0.02737	NM_006373	ILMN_1700690	Hs.514199	10493	17
Vitamin K epoxide reductase complex, subunit 1	VKORC1	3.795966	0.107118	3.343811	0.08478	1.37	Down	0.01621	NM_024006	ILMN_1739946	Hs.324844	79001	16
Vitamin K epoxide reductase complex, subunit 1	VKORC1	0.842951	0.105596	0.518579	0.05564	1.25	Down	0.03475	NM_024006	ILMN_1786139	Hs.324844	79001	16
Voltage-dependent anion channel 1	VDAC1	4.232829	0.102318	3.786563	0.09001	1.36	Down	0.01693	NM_003374	ILMN_2175601	Hs.519320	7416	5
Von Hippel-Lindau binding protein 1	VBP1	3.256458	0.124665	2.904861	0.05402	1.28	Down	0.04133	NM_003372	ILMN_2223010	Hs.436803	7411	X
Von Willebrand factor A domain containing 5A	VWA5A	1.244456	0.071928	0.965123	0.07916	1.21	Down	0.04003	NM_198315	ILMN_1682996	Hs.152944	4013	11
WAS protein family homolog 3 pseudogene	-	0.260022	0.018317	0.388919	0.03932	1.09	Up	0.02491	NR_003659	ILMN_3242416	-	-	-
WD repeat domain 54	WDR54	0.482992	0.058112	0.305868	0.03276	1.13	Down	0.03777	NM_032118	ILMN_1658289	Hs.643480	84058	2
WD repeat domain 85	WDR85	0.132888	0.035147	0.237635	0.0098	1.08	Up	0.0284	NM_138778	ILMN_1689932	Hs.292570	92715	9
WDYHV motif containing 1	WDYHV1	0.529565	0.080655	0.278173	0.05643	1.19	Down	0.04326	NM_018024	ILMN_3235472	Hs.18029	55093	8
Y box binding protein 1	YBX1	4.027473	0.07741	3.66243	0.07143	1.29	Down	0.01337	NM_004559	ILMN_2124769	Hs.473583	4904	1
Zinc finger CCCH-type containing 14	ZC3H14	0.445904	0.041703	0.22904	0.0613	1.16	Down	0.02646	NM_024824	ILMN_1714805	Hs.325846	79882	14
Zinc finger protein 16	ZNF16	0.470203	0.036638	0.576362	0.01793	1.08	Up	0.04052	NM_006958	ILMN_1669275	Hs.493225	7564	8
Zinc finger protein 238	ZNF238	0.916804	0.029722	1.105904	0.05255	1.14	Up	0.02026	NM_205768	ILMN_2399686	Hs.69997	10472	1
Zinc finger protein 549	ZNF549	0.619852	0.011035	0.501838	0.02277	1.09	Down	0.00345	NM_153263	ILMN_1718042	Hs.564295	3E+05	19
Zinc finger protein 692	ZNF692	0.739572	0.028326	0.845621	0.02971	1.08	Up	0.04159	NM_017865	ILMN_1800750	Hs.377705	55657	1
Zinc finger protein 765	ZNF765	0.27282	0.041417	0.410106	0.0346	1.1	Up	0.04386	NM_001040185	ILMN_3251460	Hs.433293	91661	19
Zinc ribbon domain containing 1	ZNRD1	1.00584	0.049599	0.740295	0.0818	1.2	Down	0.03217	NM_014596	ILMN_2398587	Hs.57813	30834	6
Zonadhesin	ZAN	0.249104	0.054622	0.411533	0.01061	1.12	Up	0.02666	NM_003386	ILMN_2273902	Hs.307004	7455	7
		Up Regulated:	252										
		Down Regulat	487										
